# Social Media–Delivered Patient Education to Enhance Self-management and Attitudes of Patients with Type 2 Diabetes During the COVID-19 Pandemic: Randomized Controlled Trial

**DOI:** 10.2196/31449

**Published:** 2022-03-23

**Authors:** Cheng Man Leong, Ting-I Lee, Yu-Mei Chien, Li-Na Kuo, Yu-Feng Kuo, Hsiang-Yin Chen

**Affiliations:** 1 Department of Clinical Pharmacy School of Pharmacy Taipei Medical University Taipei Taiwan; 2 Division of Endocrinology and Metabolism Department of Internal Medicine Wan Fang Hospital, Taipei Medical University Taipei Taiwan; 3 Division of Endocrinology and Metabolism, Department of Internal Medicine School of Medicine, College of Medicine Taipei Medical University Taipei Taiwan; 4 Department of Pharmacy Wan Fang Hospital, Taipei Medical University Taipei Taiwan

**Keywords:** diabetes, COVID-19, education, video, social media, health literacy, self-care activity, type 2 diabetes, attitude, mHealth

## Abstract

**Background:**

The use of mobile health technologies has been necessary to deliver patient education to patients with diabetes during the COVID-19 pandemic.

**Objective:**

This open-label randomized controlled trial evaluated the effects of a diabetes educational platform—Taipei Medical University–LINE Oriented Video Education—delivered through a social media app.

**Methods:**

Patients with type 2 diabetes were recruited from a clinic through physician referral. The social media–based program included 51 videos: 10 about understanding diabetes, 10 about daily care, 6 about nutrition care, 21 about diabetes drugs, and 4 containing quizzes. The intervention group received two or three videos every week and care messages every 2 weeks through the social media platform for 3 months, in addition to usual care. The control group only received usual care. Outcomes were measured at clinical visits through self-reported face-to-face questionnaires at baseline and at 3 months after the intervention, including the Simplified Diabetes Knowledge Scale (true/false version), the Diabetes Care Profile–Attitudes Toward Diabetes Scales, the Summary of Diabetes Self-Care Activities, and glycated hemoglobin (HbA_1c_) levels. Health literacy was measured at baseline using the Newest Vital Sign tool. Differences in HbA_1c_ levels and questionnaire scores before and after the intervention were compared between groups. The associations of knowledge, attitudes, and self-care activities with health literacy were assessed.

**Results:**

Patients with type 2 diabetes completed the 3-month study, with 91 out of 181 (50.3%) patients in the intervention group and 90 (49.7%) in the control group. The change in HbA_1c_ did not significantly differ between groups (intervention group: mean 6.9%, SD 0.8% to mean 7.0%, SD 0.9%, *P*=.34; control group: mean 6.7%, SD 0.6% to mean 6.7%, SD 0.7%, *P*=.91). Both groups showed increased mean knowledge scores at 12 weeks, increasing from 68.3% (SD 16.4%) to 76.7% (SD 11.7%; *P*<.001) in the intervention group and from 64.8% (SD 18.2%) to 73.2% (SD 12.6%; *P*<.001) in the control group. Positive improvements in attitudes and self-care activities were only observed in the intervention group (attitudes: mean difference 0.2, SD 0.5, *P*=.001; self-care activities: mean difference 0.3, SD 1.2, *P*=.03). A 100% utility rate was achieved for 8 out of 21 (38%) medication-related videos. Low health literacy was a significant risk factor for baseline knowledge scores in the intervention group, with an odds ratio of 2.80 (95% CI 1.28-6.12; *P*=.01); this became insignificant after 3 months.

**Conclusions:**

The social media–based program was effective at enhancing the knowledge, attitudes, and self-care activities of patients with diabetes. This intervention was also helpful for patients with low health literacy in diabetes knowledge. The program represents a potentially useful tool for delivering diabetes education to patients through social media, especially during the COVID-19 pandemic.

**Trial Registration:**

ClinicalTrials.gov NCT04876274; https://clinicaltrials.gov/ct2/show/results/NCT04876274

## Introduction

### Background

The development of effective diabetes education programs for patient self-management has been a challenge for health care professionals. The sudden outbreak of COVID-19 leading to the pandemic has created further difficulty by limiting face-to-face diabetes education. The complicated pathology of diabetes requires not only pharmacotherapy, but also firm patient engagement with daily self-care. Studies have demonstrated that patients with better disease-related knowledge, attitudes, practice, and self-efficacy have better glycemic control [1]. However, it is extremely difficult for patients with diabetes to maintain a healthy lifestyle and effective self-management without professional assistance. The excessive workload in health care settings during the COVID-19 pandemic has limited the time available to provide sufficient patient education. The increasing prevalence rates of diabetes worldwide reflects the unmet need for health education, which calls for innovative and effective mobile health (mHealth) educational programs.

The content of diabetes education is very complicated, as demonstrated by Diabetes Self-Management Education and Support services [2] and the American Association of Diabetes Educators 7 Self-Care Behaviors (AADE7) [3]. To cover all aspects required for day-to-day living with diabetes, it is recommended that education include healthy eating, regular physical activities, self-monitoring of blood glucose, compliance with medications, problem-solving skills, healthy coping skills, and other risk-reducing behaviors [3]. These aspects have been theoretically proven to effectively enhance health outcomes [4-8]. However, educating patients on all of these topics might not be practical in busy clinical settings. The time needed to teach diabetes self-care is reported to be around 4 hours per patient, which has been difficult to achieve in clinical practice during the COVID-19 pandemic [9]. Remote learning using advanced technologies may potentially provide a key solution to address the pressures created by the burden of face-to-face education.

mHealth technology has increasingly been integrated into health care to meet the demands of diabetic care during the COVID-19 pandemic. It has been shown to be effective at enhancing health outcomes, such as medication adherence, glycated hemoglobin (HbA_1c_) levels, and self-management [10-18]. Studies have shown that multimedia education using videos is more effective than written information in terms of engagement and information uptake [19,20]. Videos can deliver information through visual and audio elements and require less cognitive effort for information processing [19,20]. However, no previous study has attempted to develop a video program for diabetes education based on social media with a two-way communication component.

### Objectives

In this study, we developed an educational program based on the AADE7 [3]: Taipei Medical University–LINE Oriented Video Education (TMU-LOVE). The social media platform LINE (LINE Corporation) was employed in this study. The platform is one of the most popular and user-friendly social media platforms in Taiwan, with a high acceptance rate of up to 88% among the population between the ages of 16 and 64 years [21]. This randomized controlled trial (RCT) was conducted during the COVID-19 pandemic. The first aim was to evaluate the effectiveness of the social media–based education program in regard to changes in patients’ HbA_1c_ levels, knowledge, attitudes, and self-care activities before and after the intervention. Another aim was to study the effects of this social media–based education program on changes in knowledge by diabetes patients with high and low levels of health literacy.

## Methods

### Study Design

This study was an open-label RCT and was conducted between July 2020 and January 2021. The study was registered at ClinicalTrials.gov (NCT04876274).

### Ethics Approval

This study was approved by the Taipei Medical University (TMU)–Joint Institutional Review Board (No. N201905088). The study followed the CONSORT-EHEALTH (Consolidated Standards of Reporting Trials of Electronic and Mobile Health Applications and Online Telehealth) guidelines [22].

### Participants

Participants were recruited by physicians at the Endocrinology and Metabolism Clinic of Wan-Fang Hospital, Taipei, Taiwan. Invitations to join the study were extended to all patients with diabetes, aged 20 years or older, who had at least one HbA_1c_ measurement that was equal to or greater than 6% in the past 6 months and possessed a smartphone with the LINE app installed. Patients who had gestational diabetes, had cognitive impairment, or were controlling their diabetes through dietary control without medication were excluded. Based on a power of 80% and a 2-sided α level of .05, the required sample size was estimated to be at least 128 participants using G*Power (version 3.1; Heinrich-Heine-Universität Düsseldorf) [23]. To adjust for an estimated nonresponse rate of 20%, we aimed for a final sample size of at least 160 participants, with 80 patients in each group.

Eligible participants were asked to complete a written consent form before joining the study. Demographic information (ie, age, sex, educational level, income, medications, and personal history) and health status (ie, diagnosis time and BMI) were obtained using electronic medical records. Pharmacists allocated participants to either the control group or the intervention group at a 1:1 ratio according to a random sequence, which was generated by a digital program before launching the study. The patients in both groups were scheduled to return to the clinic at the end of the 3-month study period, where their chronic prescription medications were re-evaluated. Diabetes health education for the control group comprised provision of the usual diabetes health care, including patient consultations with physicians, access to nurses in the outpatient services, as well as medication consultations with pharmacists upon receiving prescriptions. Physicians also referred patients for consultations with certified diabetes educators (CDEs) when necessary.

### Intervention

**Figure 1:**
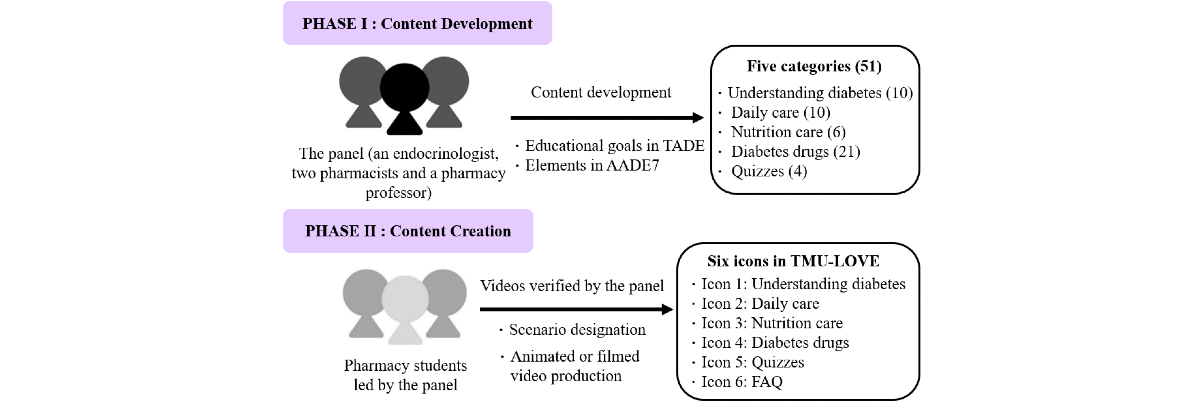
Program development. The number of videos in each category are reported within parentheses. AADE7: American Association of Diabetes Educators 7 Self-Care Behaviors; FAQ: frequently asked questions; TADE: Taiwanese Association of Diabetes Educators; TMU-LOVE: Taipei Medical University–LINE Oriented Video Education.

The patients allocated to the intervention group were granted access to the TMU-LOVE program through a QR code (Figure S1 in Multimedia Appendix 1), in addition to receiving the usual health care. This program was developed by the TMU School of Pharmacy and is illustrated in Figure 1. In the first phase of development, a panel consisting of an endocrinologist, two pharmacists, and a pharmacy professor developed an outline of the program according to the guidelines of the Taiwanese Association of Diabetes Educators and seven key points in the AADE7 [3]. The outline contained five categories: understanding diabetes, daily care, nutrition care, diabetes drugs, and quizzes. The elements of each section were also determined.

A group of pharmacy students joined the second phase to create the videos. The students started to write scenarios and discussed each video with the pharmacy professor and pharmacists. After checking the evidence for the scenarios, the students produced animated or filmed videos. The videos were reviewed and revised by the panel until they met the learning goals in the outline. A total of 51 videos were produced: 10 videos were about understanding diabetes, 10 were about daily care, 6 were about nutrition care, 21 were about diabetes drugs, and 4 were about diabetes knowledge–related quizzes (Figure S2 in Multimedia Appendix 1). The medications described in the program were based on the formulary at Wan-Fang Hospital, including metformin, acarbose, dipeptidyl peptidase-IV inhibitors, meglitinide, sulfonylurea, thiazolidinedione, sodium-glucose cotransporter-2 inhibitors, and insulin. All videos are listed in Multimedia Appendix 2 and are available at the TMU School of Pharmacy’s website [24].

The menu for the educational platform was composed of six icons; these represented the five video categories and a FAQ (frequently asked questions) section (Figure S3 in Multimedia Appendix 1). The program was also equipped with a one-on-one chat room, graphic messages, and the ability to direct patients to certain websites [25]. The study employed the messaging feature of the LINE social media platform to send educational videos and communicate with patients by text message, voice, or video call. Patients could also click on the six icons in the menu to connect to the website of the program. The patients could use LINE’s messaging feature to ask questions, and pharmacists could answer them through text messages or voice calls via the social media platform.

**Figure 2:**
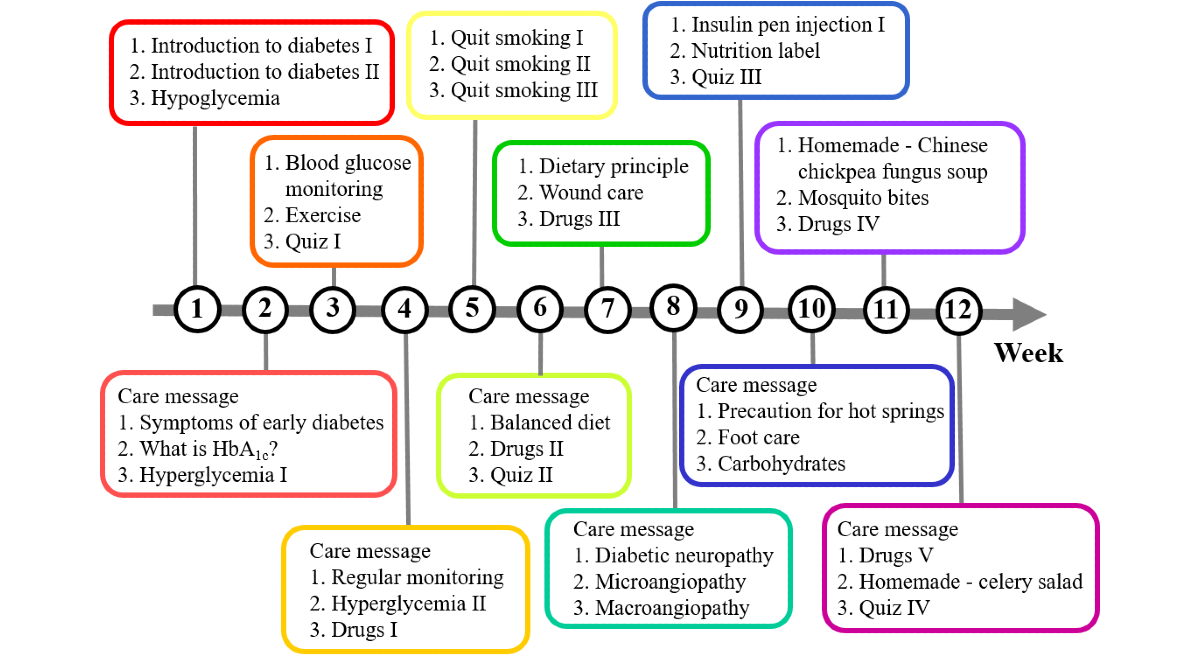
Delivery schedule of videos. HbA_1c_: glycated hemoglobin.

The program was designed to last for a study duration of 3 months (ie, 12 weeks), with two or three videos sent every week and a care message sent every 2 weeks to the patients in the intervention group. All patients received the same videos except for the videos on medications. Videos regarding the basic understanding of diabetes were delivered in weeks 1 to 4. Videos on daily care were first delivered in week 5, and six nutrition care videos were scheduled over six different weeks. Each patient received the videos for their individual medications in weeks 4, 6, 7, 11, and 12. Four quizzes were scheduled to be delivered in weeks 3, 6, 9, and 12 (Figure 2). Patients could also access all of the videos through the social media platform whenever they desired.

### Outcomes and Measures

#### Measures for Blood Sugar Control

The outcome for blood sugar control was the change of HbA_1c_ level from baseline to month 3. HbA_1c_ level results were obtained from the electronic medical records for all potential participants before the allocation and determination of eligibility. The participants were requested to take an HbA_1c_ test at 3 months, and all HbA_1c_ level results during the 3-month study period were examined for the analysis.

#### Knowledge, Attitudes, and Self-care Activities Toward Diabetes

The Chinese version of the Simplified Diabetes Knowledge Scale (SDKS; true/false version) [26] was used to measure diabetes patients’ knowledge. This version of the scale contains 24 items and was validated in previous studies [26,27].

Patients’ attitudes toward diabetes were assessed using the Chinese version of the Diabetes Care Profile–Attitudes Toward Diabetes Scales (DCP-ATDS) [26]. This questionnaire was previously validated and translated [26,28]. It has 17 items that are divided into the following sections: positive attitudes, negative attitudes, self-care ability, self-care adherence, and importance of care. Each item is graded on a 5-point Likert scale using the following response options: “strongly agree,” “agree,” “neutral,” “disagree,” or “strongly disagree.”

Self-care activities were measured using the Chinese version of the Summary of Diabetes Self-Care Activities (SDSCA) [29], which consists of 10 items and has been validated [29,30]. In the SDSCA, patients were asked how many days per week they had performed the correct behaviors regarding medication adherence, diet, exercise, self-monitoring of blood glucose, and foot care.

#### Health Literacy

To determine whether the social media–based program could be applied to patients with different levels of health literacy, the effects of health literacy on SDKS, SDSCA, and DCP-ATDS outcomes were compared in the intervention and control groups. We assessed health literacy at baseline using the Newest Vital Sign (NVS) tool, which was developed and translated in previous studies [31,32]. This scale consists of six questions based on the nutrition label of an ice cream product in order to evaluate both the reading and numeracy of the patients. The patients were categorized as having high or low health literacy based on a cutoff point of 2 for the sum of the NVS score.

#### Utility Rate of Educational Videos

The utility rate was used to represent the frequency of watching videos in each of the five categories. The utility rate for accessing educational videos was measured using the number of views of each video divided by the number of target participants. Utility rates of videos with a calculated rate above 100% were counted as 100%.

### Statistical Analysis

Statistical analyses were conducted using SPSS Statistics for Windows (version 28.0; IBM Corp). The study was performed using intention-to-treat analysis. Multiple imputation was employed to manage missing values [33-35]. All tests were 2-tailed, with a significance level of .05. Differences in baseline characteristics were examined using descriptive analysis. The Kolmogorov-Smirnov test was used to verify whether the variables had normal distributions. According to the results of the Kolmogorov-Smirnov test, a Wilcoxon signed-rank test or paired *t* tests were used to examine the differences in the pretest and posttest scores within a group, while a Mann-Whitney *U* test or unpaired *t* tests were performed to compare the differences between groups. An ordinal logistic regression model was applied to evaluate the association of health literacy with knowledge, attitudes, and self-care activities.

## Results

### Participant Characteristics

**Figure 3:**
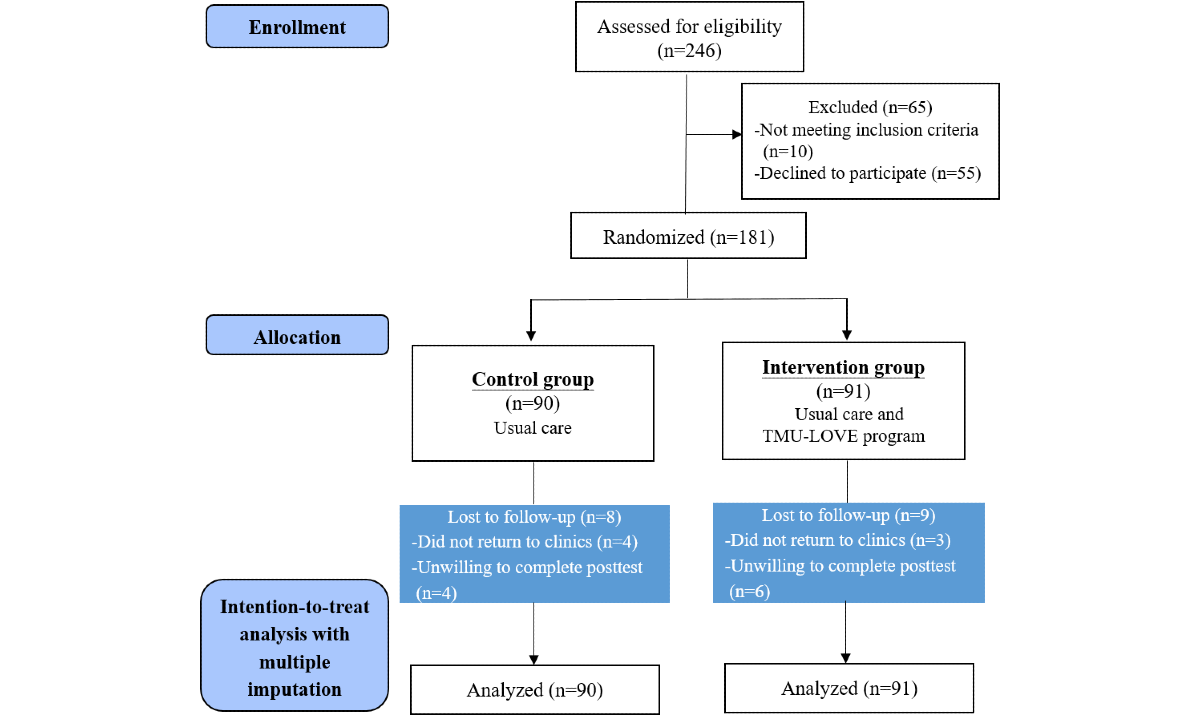
The CONSORT flow diagram. CONSORT: Consolidated Standards of Reporting Trials; TMU-LOVE: Taipei Medical University–LINE Oriented Video Education.

A total of 246 patients from the Endocrinology and Metabolism Clinic of Wan-Fang Hospital were screened for this study. Patients who were excluded from the study included 10 patients with HbA_1c_ levels less than 6%, 3 patients who did not complete the informed consent form, and 52 patients who declined to participate. In total, 181 patients were randomized equally into the control group (n=90, 49.7%) or the intervention group (n=91, 50.3%), as shown in Figure 3.

Table 1 shows the demographic characteristics of the patients, which indicate that there were no significant differences observed between groups. The percentage of patients receiving CDE care in the two groups differed insignificantly. The mean age of the participants was 58.6 (SD 11.6) years, and there were 57 males out of 90 participants (63.3%) in the control group and 67 males out of 91 participants (73.6%) in the intervention group. More than half of the participants were highly educated. The mean HbA_1c_ level was 6.8% (SD 0.7%), and the mean health literacy level was 1.7 (SD 1.9).

**Table 1:** Baseline characteristics of the participants.

Variable		All participants (N=181)	Control group (n=90)	Intervention group (n=91)	*P* value
Age (years), mean (SD)		58.6 (11.6)	58.1 (11.9)	59.0 (11.4)	.60
Male, n (%)		124 (68.5)	57 (63.3)	67 (73.6)	.15
BMI^a^, mean (SD)		26.4 (4.6)	26.7 (5.0)	26.1 (4.2)	.39
**Educational level, n (%)**					
	Junior high school or below	27 (14.9)	13 (14.4)	14 (15.4)	.93^b^
	Senior high school	50 (27.6)	24 (26.7)	26 (28.6)	
	College or higher	104 (57.5)	53 (58.9)	51 (56.0)	
Unemployed, n (%)		76 (42.0)	40 (44.4)	36 (39.6)	.55
Living alone, n (%)		6 (3.3)	3 (3.3)	3 (3.3)	.60
**Annual income (NT$^c^), n (%)**					
	Unwaged	58 (32.0)	30 (33.3)	28 (30.8)	.48
	<500,000	43 (23.8)	25 (27.8)	18 (19.8)	
	500,000-1,000,000	48 (26.5)	21 (23.3)	27 (29.7)	
	>1,000,000	32 (17.7)	14 (15.6)	18 (19.8)	
**Medication type, n (%)**					
	Oral medication only	140 (77.3)	72 (80)	68 (74.7)	.06
	Insulin only	9 (5.0)	1 (1.1)	8 (8.8)	
	Oral medication and insulin	32 (17.7)	17 (18.9)	15 (16.5)	
Diagnosis time (years), mean (SD)		10.3 (8.5)	10.3 (8.1)	10.4 (9.0)	.91
HbA_1c_^d^ level (%), mean (SD)		6.8 (0.7)	6.7 (0.6)	7.0 (0.8)	.07
Smoking, n (%)		25 (13.8)	15 (16.7)	10 (11.0)	.23
Alcohol consumption, n (%)		65 (35.9)	29 (32.2)	36 (39.6)	.78
SMBG^e^, n (%)		79 (43.6)	42 (46.7)	37 (40.7)	.46
Physical exercise, n (%)		133 (73.5)	63 (70)	70 (76.9)	.77
Receiving CDE^f^ education within 3 months, n (%)		43 (23.8)	20 (22.2)	23 (25.3)	.73
NVS^g^ questionnaire total score, mean (SD)		1.7 (1.9)	1.8 (2.0)	1.7 (1.8)	.71

^a^BMI is calculated as weight in kilograms divided by height in meters squared.

^b^*P* values for a group are reported in the top row of that group.

^c^$1 NT = $0.035 USD.

^d^HbA_1c_: glycated hemoglobin.

^e^SMBG: self-monitoring blood glucose.

^f^CDE: certified diabetes educator.

^g^NVS: Newest Vital Sign; questionnaire total scores range from 0 to 6, with a higher score indicating a favorable state.

### Outcomes

#### Measures for Blood Sugar Control

After 3 months of the intervention, the mean HbA_1c_ level changed insignificantly from 6.9% (SD 0.8%) to 7% (SD 0.9%; *P*=.34) in the intervention group and from 6.7% (SD 0.6%) to 6.7% (SD 0.7%; *P*=.91) in the control group (Table 2).

**Table 2:** Clinical and nonclinical outcomes after the intervention.

Variable		Within groups^a^					Between groups^b^		
		Baseline, mean (SD)	3 months, mean (SD)	*P* value	Mean difference (SD)	Mean difference (SD)		95% CI	*P* value
**HbA_1c_^c^ (%)**									
	Intervention group (n=91)	6.9 (0.8)	7.0 (0.9)	.34	0.07 (0.7)	0.06 (0.1)^d^		–0.25 to 0.14^d^	.57^d^
	Control group (n=90)	6.7 (0.6)	6.7 (0.7)	.91	0.01 (0.7)				
**SDKS^e^ (% correctness)**									
	Intervention group (n=91)	68.3 (16.4)	76.7 (11.7)	<.001	8.4 (14.7)	0.01 (2.3)		–4.52 to 4.53	.99
	Control group (n=90)	64.8 (18.2)	73.2 (12.6)	<.001	8.4 (16.1)				
**DCP-ATDS^f^**									
	Intervention group (n=91)	3.6 (0.4)	3.8 (0.5)	.001	0.2 (0.5)	0.2 (0.06)		–0.32 to –0.07	.003
	Control group (n=90)	3.7 (0.4)	3.7 (0.5)	.58	–0.02 (0.4)				
**SDSCA^g^**									
	Intervention group (n=91)	3.7 (1.3)	4.0 (1.2)	.03	0.3 (1.2)	0.3 (0.2)		–0.65 to –0.02	.04
	Control group (n=90)	3.9 (1.4)	3.9 (1.5)	.52	–0.07 (1.2)				

^a^Paired *t* tests (2-tailed) were performed for HbA_1c_ levels, DCP-ATDS scores, and SDSCA scores; a Wilcoxon signed-rank test was performed for percentage of correct SDKS questions.

^b^Unpaired *t* tests (2-tailed) were performed for HbA_1c_ levels, DCP-ATDS scores, and SDSCA scores; a Mann-Whitney *U* test was performed for percentage of correct SDKS questions.

^c^HbA_1c_: glycated hemoglobin.

^d^Values for a group are reported in the top row of that group.

^e^SDKS: Simplified Diabetes Knowledge Scale (true/false version). Percent correctness ranges from 0% to 100%, and a higher score indicates a favorable state.

^f^DCP-ATDS: Diabetes Care Profile–Attitudes Toward Diabetes Scales. Mean scores range from 1 to 5, and a higher score indicates a favorable state; pretest scores were normally distributed, based on the Kolmogorov-Smirnov test.

^g^SDSCA: Summary of Diabetes Self-Care Activities. Mean scores range from 0 to 7, and a higher score indicates a favorable state; pretest scores were normally distributed, based on the Kolmogorov-Smirnov test.

#### Knowledge, Attitudes, and Self-care Activities Toward Diabetes

Both groups experienced significant improvements in SDKS mean scores, from 68.3% (SD 16.4%) to 76.7% (SD 11.7%; *P*<.001) in the intervention group and from 64.8% (SD 18.2%) to 73.2% (SD 12.6%; *P*<.001) in the control group. However, the difference in knowledge scores between the two groups was not significant (intervention, 8.4% vs control, 8.4%; *P*=.85). The scores for each item of the SDKS are shown in Multimedia Appendix 3.

The intervention group exhibited significant growth in the overall attitude mean score, from 3.6 (SD 0.4) to 3.8 (SD 0.5; *P*=.001), whereas no significant change was observed in the control group, from 3.7 (SD 0.4) to 3.7 (SD 0.5; *P*=.58). The difference in the change of DCP-ATDS scores between baseline and follow-up was significant between groups (mean difference 0.2, 95% CI –0.32 to –0.07; *P*=.003). Detailed data for the DCP-ATDS are presented in Multimedia Appendix 4.

Regarding self-care activities measured by the SDSCA, significant improvement was observed in the intervention group only, from a mean score of 3.7 (SD 1.3) to 4.0 (SD 1.2; *P*=.03); the improvement in scores was significantly higher in comparison to the control group (mean 0.3, SD 0.2; *P*=.04). Tables S1 and S2 in Multimedia Appendix 5 show the SDSCA scores and the top five reasons mentioned by patients for finding it difficult to engage in self-care activities. In further analyses, patients with and without improvement in SDSCA scores after 3 months had no significant differences in any of DCP-ATDS subscales, including the subscales of positive attitudes and negative attitudes (Multimedia Appendix 6).

#### Impact of Health Literacy

Table 3 shows the effects of health literacy on knowledge, attitudes, and self-care activities associated with the SDKS. At baseline, the odd ratios (ORs) of health literacy were 2.8 (95% CI 1.28-6.12; *P*=.01) for the intervention group and 5.43 (95% CI 2.41-12.25; *P*<.001) for the control group. The social media–based education intervention eased the influence of health literacy on SDKS results, which disappeared in the intervention group after 3 months, with a *P* value of .75. The OR remained significant in the control group after 3 months (OR 2.43, 95% CI 1.14-5.19; *P*=.02).

**Table 3:** Effects of health literacy on knowledge, attitudes, and self-care activities.

Variables	Intervention group (n=91)						Control group (n=90)				
	Baseline		3 months			Baseline				3 months	
	OR^a^ (95% CI)	*P* value	OR (95% CI)	*P* value	OR (95% CI)			*P* value	OR (95% CI)		*P* value
SDKS^b^	2.80 (1.28-6.12)	.01	0.88 (0.41-1.91)	.75	5.43 (2.41-12.25)			<.001	2.43 (1.14-5.19)		.02
DCP-ATDS^c^	1.60 (0.75-3.41)	.23	1.45 (0.66-3.21)	.36	0.77 (0.37-1.62)			.49	0.90 (0.42-1.91)		.64
SDSCA^d^	0.44 (0.21-0.95)	.04	0.76 (0.36-1.62)	.48	0.95 (0.45-2.00)			.89	0.92 (0.43-1.95)		.82

^a^OR: odds ratio.

^b^SDKS: Simplified Diabetes Knowledge Scale (true/false version).

^c^DCP-ATDS: Diabetes Care Profile–Attitudes Toward Diabetes Scales.

^d^SDSCA: Summary of Diabetes Self-Care Activities.

#### Utility Rate of Educational Videos

**Figure 4:**
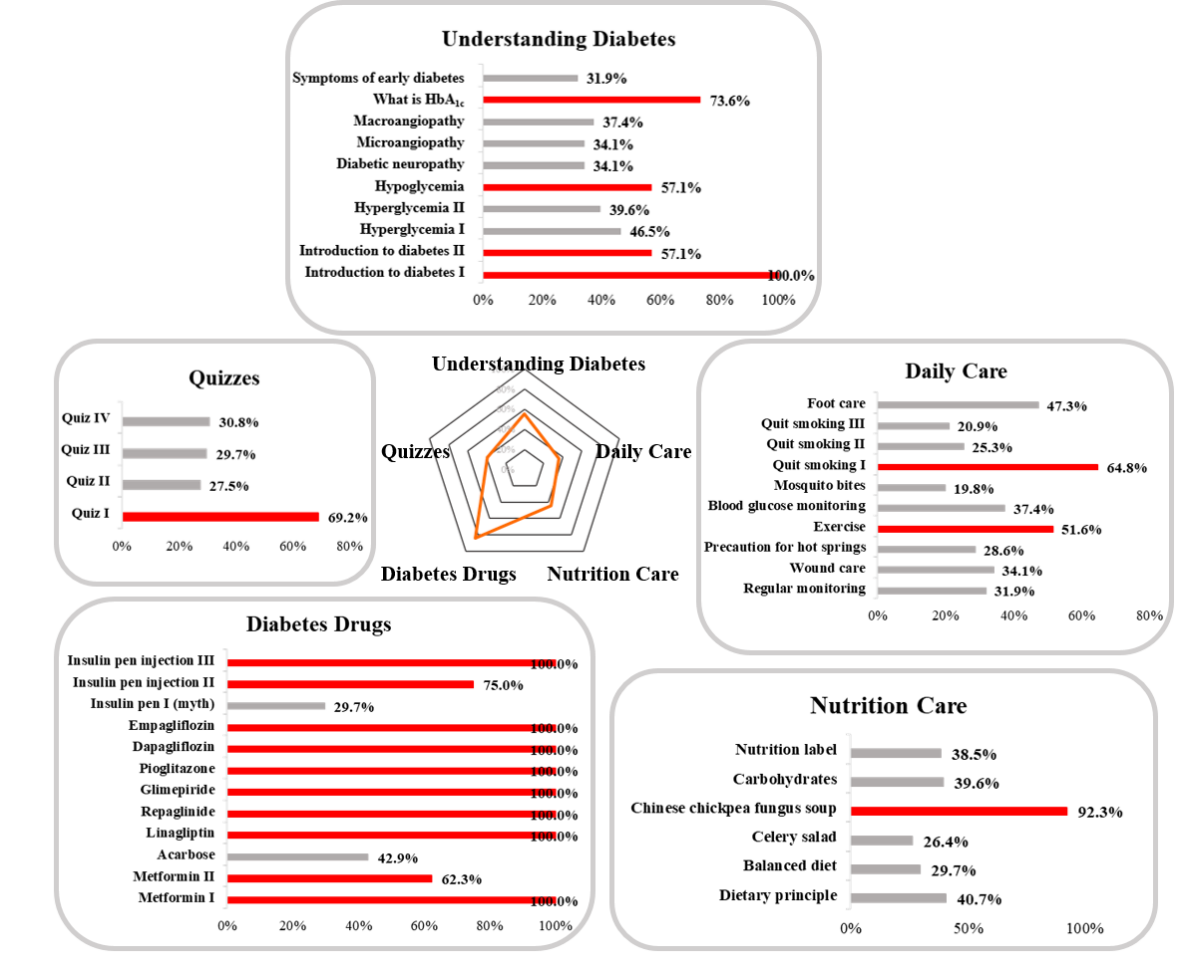
Utility rate for each video in each of the five categories. Rates are shown on each bar. HbA_1c_: glycated hemoglobin.

The utility rates for drug-related videos were the highest, with 8 out of 21 (38%) videos achieving a 100% utility rate. For the category of understanding diabetes, only 4 out of 10 (40%) videos had a utility rate above 50%. Among the 10 videos related to daily care, only 2 (20%) had a utility rate above 50%. Only 1 nutrition care video out of 6 (17%) and 1 quiz video out of 4 (25%) was highly used (Figure 4). Interestingly, the utility rate of the first quiz reached 69.2%, but the utility rates were significantly lower for the following three quizzes, ranging from 27.5% to 30.8%.

## Discussion

### Principal Findings

This study demonstrated that the delivery of a social media–based education program during the COVID-19 pandemic improved the knowledge, attitudes, and self-care activities of patients with type 2 diabetes. Among the 51 videos in the social media intervention, the utility rates of the drug-related videos were the highest, which implies a demand for medication information among patients. Interestingly, the results also revealed that the program overcame the negative effects of low health literacy, as it was no longer a significant factor in knowledge improvement after the intervention. However, the significant influence of low health literacy remained in the control group after 3 months. The program demonstrated the usefulness of social media–based diabetes education, even among those who have limited health literacy.

The attitude and practice improvement observed in this study, which was not always present with knowledge enhancement, requires stronger motivation and patient engagement. The videos in this study covered all elements of the AADE7 and drug information, resulting in reasonable knowledge improvement. Previous mHealth studies showed conflicting results regarding attitudes. One study showed that the use of mobile text messaging did not significantly benefit diabetes patients’ attitudes [36], whereas another study showed that the use of mobile texts, voice messages, and animations improved patient attitudes [37]. All videos in this study were presented with a mindset of fostering growth and positive attitudes to help patients fight against diabetes. The positive encouragement was fundamentally important for changing the mindset of patients, as the chronic nature of disease commonly decreases patients’ perseverance.

Changes in self-care activities were even more difficult to achieve, requiring a more individualized, patient-centered approach. Patient interviews in this study revealed that a group characterized by having a high workload experienced difficulties in improving their self-care activities. They ate high-fat meals and engaged in little to no exercise. Interestingly, this group had the same attitude level as patients who showed improvements in self-care activities. This finding was consistent with previous studies [38,39], indicating that many patients with diabetes have good attitudes but poor practice. Four videos with easy-to-make recipes in this study might have motivated patients to take the initiative toward self-care. Individualized discussion is essential for assisting patients with high workloads to overcome difficulties in changing their lifestyle [40].

This study identified a key lesson for operating a successful mHealth education program for patients with diabetes during the COVID-19 pandemic. Worldwide health care and patient education shifted quickly from in-person to remote online approaches due to physical distancing and social isolation restrictions [41-43]. Online telemedicine outpatient services have become a popular trend in health care settings [44,45]. Revision of the content of the social media program by adding the facility to have more frequent online discussions could be one solution to optimize diabetes health education during the COVID-19 pandemic.

This study demonstrated that patient-oriented language and animated videos were effective at being accepted by patients with inadequate health literacy. Health literacy decreases with age due to degeneration of cognitive and intellectual functions, and is positively related to a patient’s level of education [46-48]. Consistent with other studies [49,50], the level of health literacy is generally low in Taiwan. Even among our highly educated populations, the mean NVS score was still less than 2, indicating a high likelihood of limited literacy. Possible reasons might be that health education is not being emphasized in the high school curriculum or adopted as a university entrance requirement in Taiwan. The need to strengthen basic health education in formal educational settings should be considered by policy makers [50]. This study indicates that video approaches may provide an effective solution to address the generally low health literacy levels among the population and an aging society in overloaded health care settings.

Drug-related education in diabetes is an unmet clinical need. A previous study revealed that medication-related features represented four of the top 10 most demanded features by patients in an mHealth program [51]. This finding is consistent with our observation that the video utility rates were the highest for the medication category (Figure 4). According to global statistics from the Certified Diabetes Care and Education Specialist organization, pharmacists only represented 7% of all CDEs in 2020 [52]. Most CDEs in clinical settings are not pharmacists. Current mHealth education programs developed for diabetes have mainly focused on basic information, patient self-care skills, and nutrition. Development of mHealth applications with more sophisticated medication education and opportunities for patients to communicate with pharmacists could potentially help to fulfill this patient need.

### Clinical Implications

The social media–based education program in this study was effective at assisting all health care professionals who provide diabetes medication education in clinical settings. When diabetes education is provided by a nonpharmacist CDE, the program could create a link to provide medication information and a channel for consultations with pharmacists. It could save pharmacists time by delivering basic medication knowledge in videos and resolving patients’ general concerns. Remote delivery of videos through a social media platform provides an easy solution to help patients understand information in situations when face-to-face health education is not possible, such as during the COVID-19 pandemic. The intervention employed in this study could also be adapted to provide patient education for other chronic diseases in order to enhance telemedicine communication. Furthermore, it could be sustainably integrated into diabetes education programs run by nonpharmacist CDEs as well as pharmacists during and beyond COVID-19.

### Limitations

Our study has several limitations. It was a single-center study, which may have led to population bias and, thus, restricted its generalizability. The intervention only lasted 12 weeks, so we were unable to assess long-term outcomes. The patients’ attitude, knowledge, and self-care activity scores were collected by self-report, which may have created recall bias. Moreover, we could only determine that patients clicked on the videos, but we could not confirm how long and how attentively they watched them. Finally, the economic value of the social media–based program was not evaluated. Future studies covering populations that are more diverse with a longer study duration and careful analysis of digital learning metrics are needed to validate the effects of the social media–based program among patients with type 2 diabetes.

### Conclusions

This study has demonstrated the potential value of a social media–oriented video education program as a useful tool for diabetes care during the COVID-19 pandemic. It was found to have been effective in improving patients’ knowledge, attitudes, and self-care activities. The video program also overcame the issue related to inadequate health literacy, which is a time-consuming challenge in traditional face-to-face education [53]. Due to the integration of medication information and two-way communication, the social media–based program represents an innovative and efficient strategy for enhancing patients’ attitudes, knowledge, and self-care during the COVID-19 pandemic and in the future.

## Appendix

Multimedia Appendix 1 The content of the Taipei Medical University–LINE Oriented Video Education (TMU-LOVE) program.

Multimedia Appendix 2 List of all videos.

Multimedia Appendix 3 Correctness of the Simplified Diabetes Knowledge Scale (SDKS; true/false version).

Multimedia Appendix 4 Scores for the Diabetes Care Profile–Attitudes Toward Diabetes Scales (DCP-ATDS).

Multimedia Appendix 5 Summary of Diabetes Self-Care Activities (SDSCA) scores and reasons for finding it difficult to engage in self-care activities.

Multimedia Appendix 6 Comparison in subscale scores for the Diabetes Care Profile–Attitudes Toward Diabetes Scales (DCP-ATDS).

Multimedia Appendix 7 CONSORT-EHEALTH checklist (V 1.6.1).

## Abbreviations

AADE7: American Association of Diabetes Educators 7 Self-Care Behaviors
CDE: certified diabetes educator
CONSORT-EHEALTH: Consolidated Standards of Reporting Trials of Electronic and Mobile Health Applications and Online Telehealth
DCP-ATDS: Diabetes Care Profile–Attitudes Toward Diabetes Scales
FAQ: frequently asked questions
HbA_1c_: glycated hemoglobin
mHealth: mobile health
NVS: Newest Vital Sign
OR: odds ratio
RCT: randomized controlled trial
SDKS: Simplified Diabetes Knowledge Scale
SDSCA: Summary of Diabetes Self-Care Activities
TMU: Taipei Medical University
TMU-LOVE: Taipei Medical University–LINE Oriented Video Education
