# Supplementary material for: Social Media–Delivered Patient Education to Enhance Self-management and Attitudes of Patients with Type 2 Diabetes During the COVID-19 Pandemic: Randomized Controlled Trial
Source: J Med Internet Res. 2022 Mar 23;24(3):e31449. doi: 10.2196/31449 (PMC8987969; doi:10.2196/31449)

Multimedia Appendix 1. The content of TMU-LOVE program.

Figure S1. QR response code


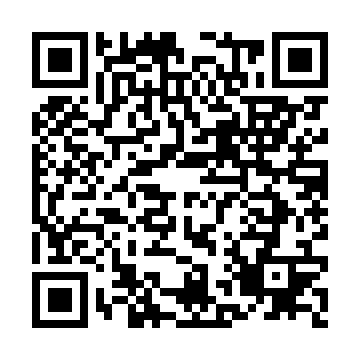


Figure S2. Video categories in the TMU-LOVE program


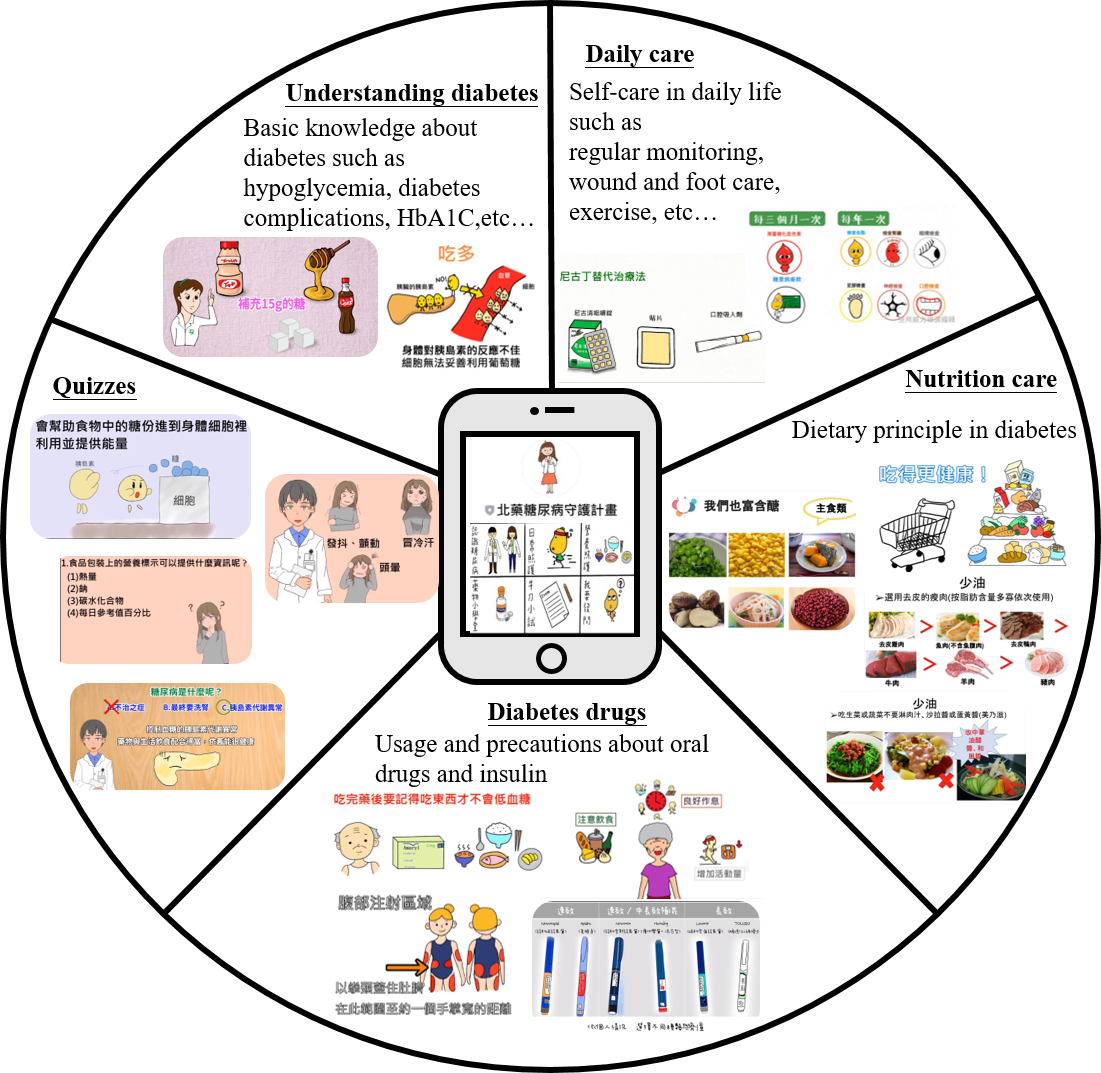


Figure S3. The menu of TMU-LOVE platform. Front layout design with six icons (left), one-on-one chat room and graphic messages (middle), and educational videos (right).


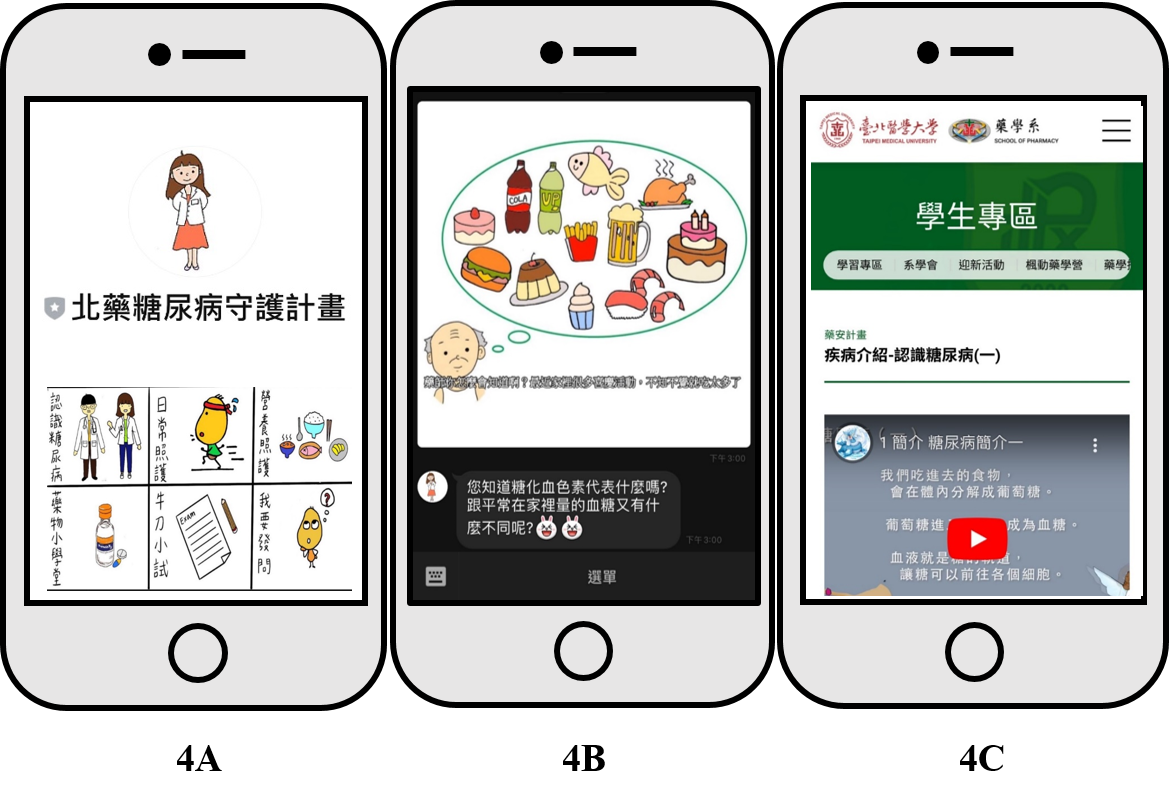

Supplement: Multimedia Appendix 1 [file jmir_v24i3e31449_app1.docx]
