# Supplementary material for: Social Media–Delivered Patient Education to Enhance Self-management and Attitudes of Patients with Type 2 Diabetes During the COVID-19 Pandemic: Randomized Controlled Trial
Source: J Med Internet Res. 2022 Mar 23;24(3):e31449. doi: 10.2196/31449 (PMC8987969; doi:10.2196/31449)
Supplement: Multimedia Appendix 2 [file jmir_v24i3e31449_app2.docx]

Multimedia Appendix 2. List of all videos.

| **Understanding diabetes** | | |
| --- | --- | --- |
| Introduction to diabetes I  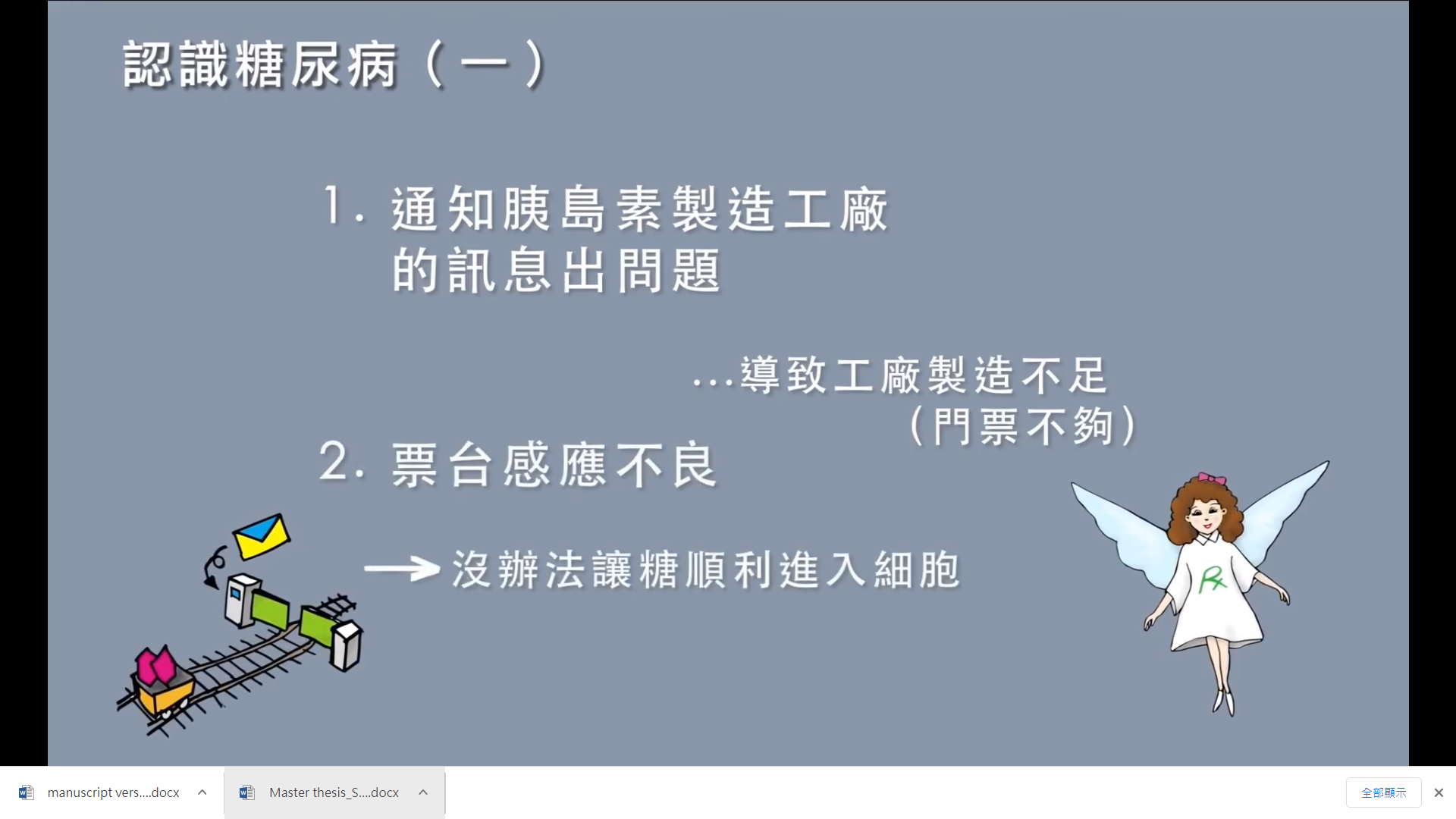 | Introduction to diabetes II  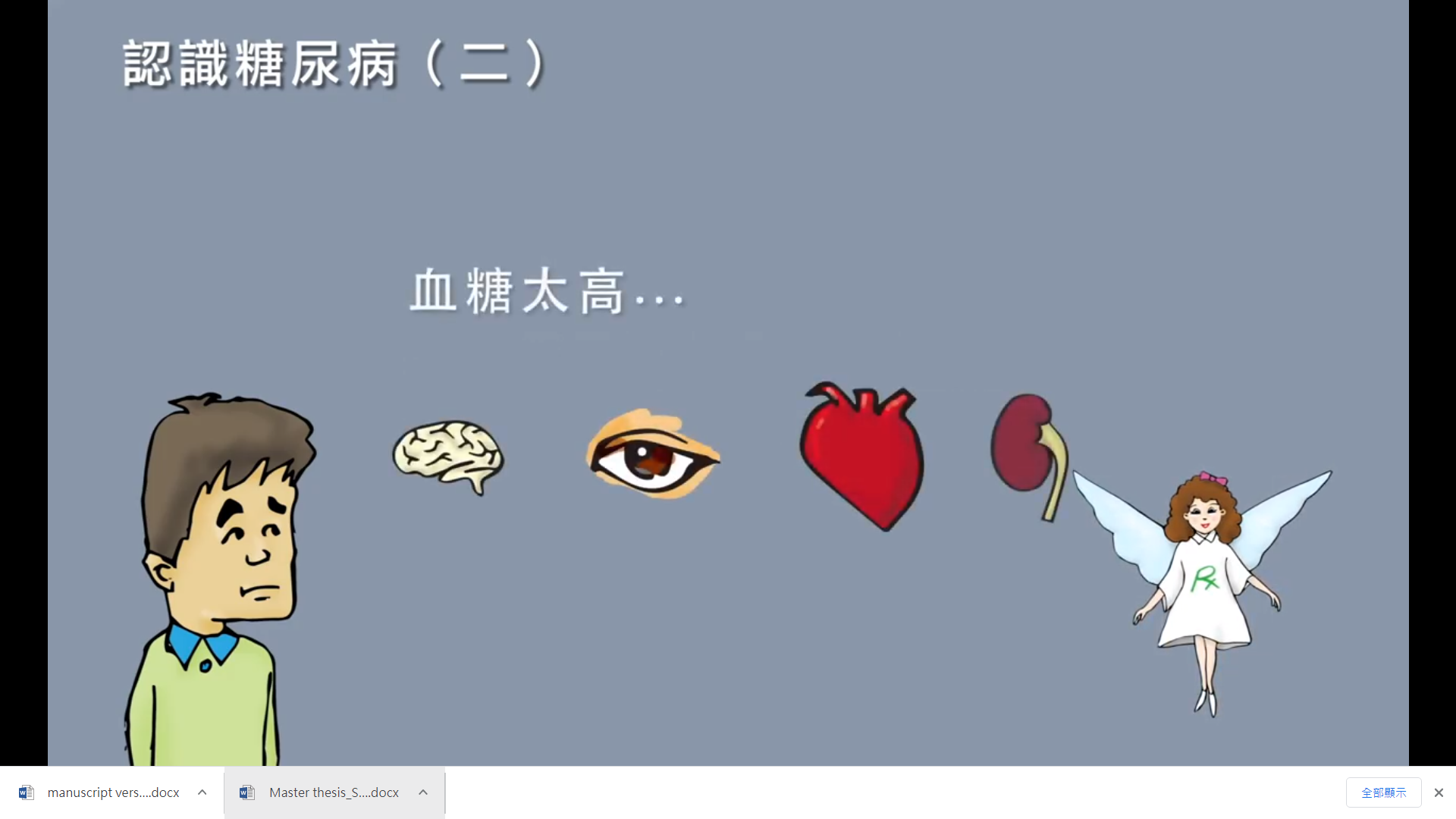 | |
| Hyperglycemia I  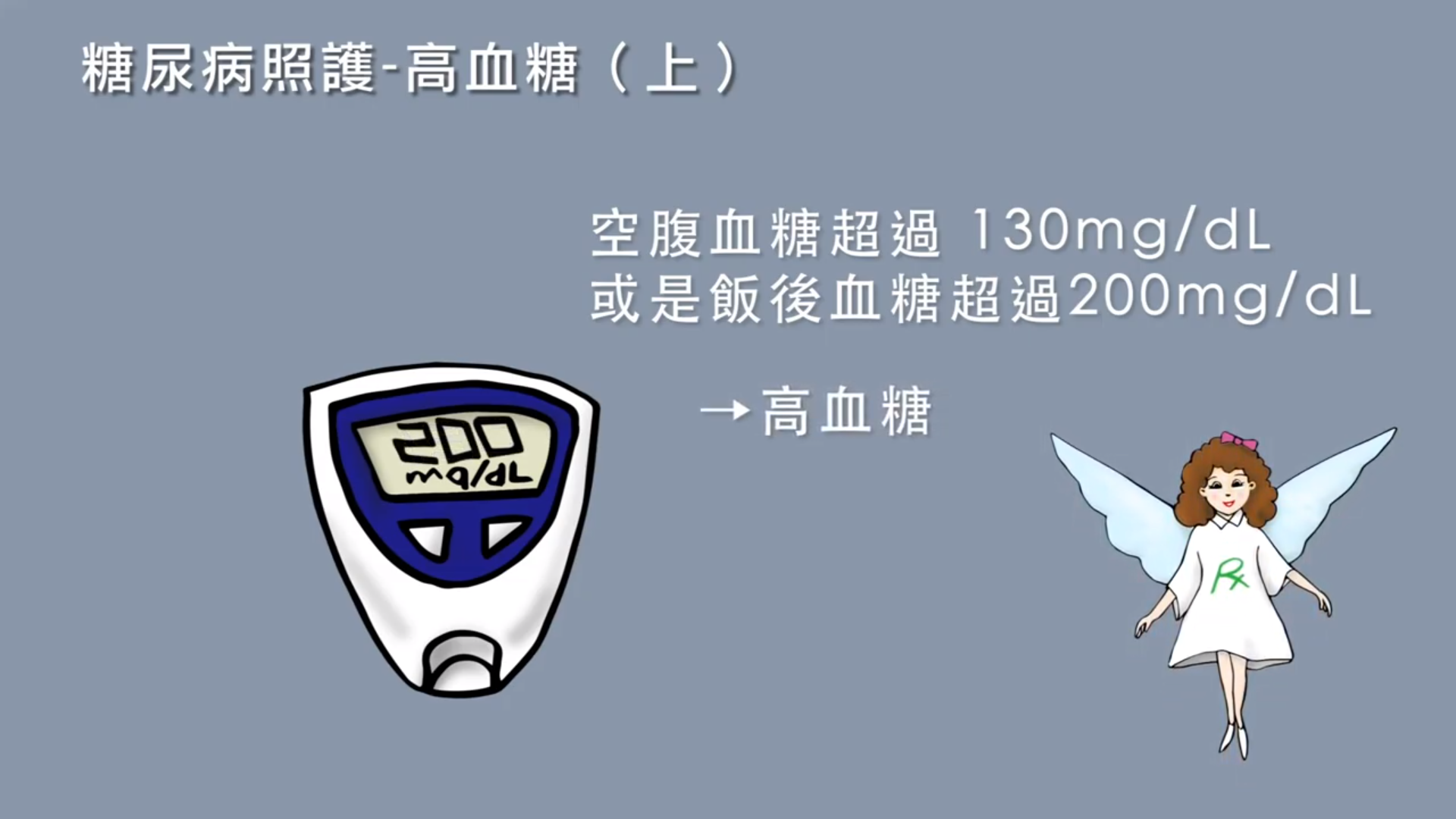 | Hyperglycemia II  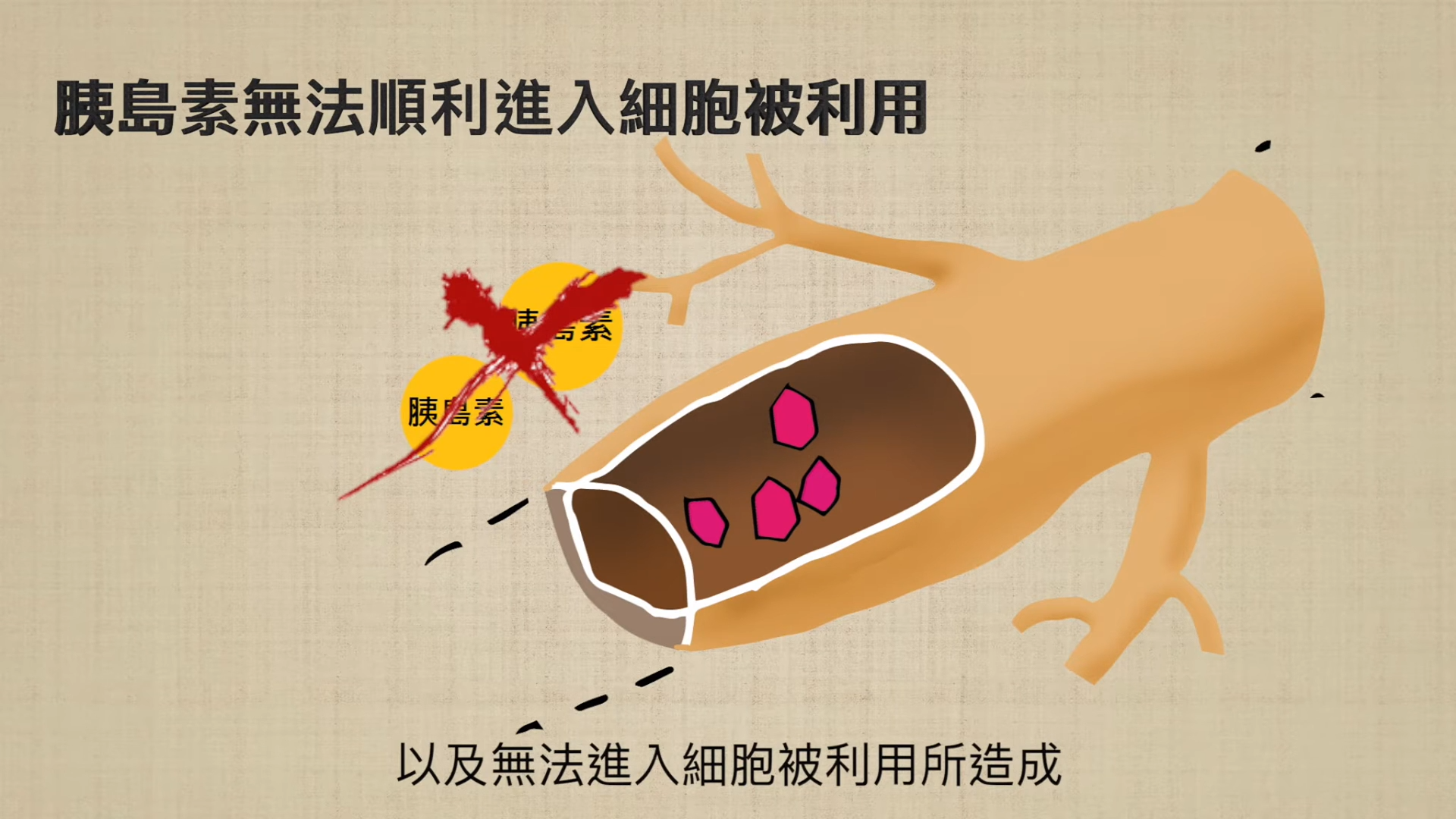 | |
| Hypoglycemia  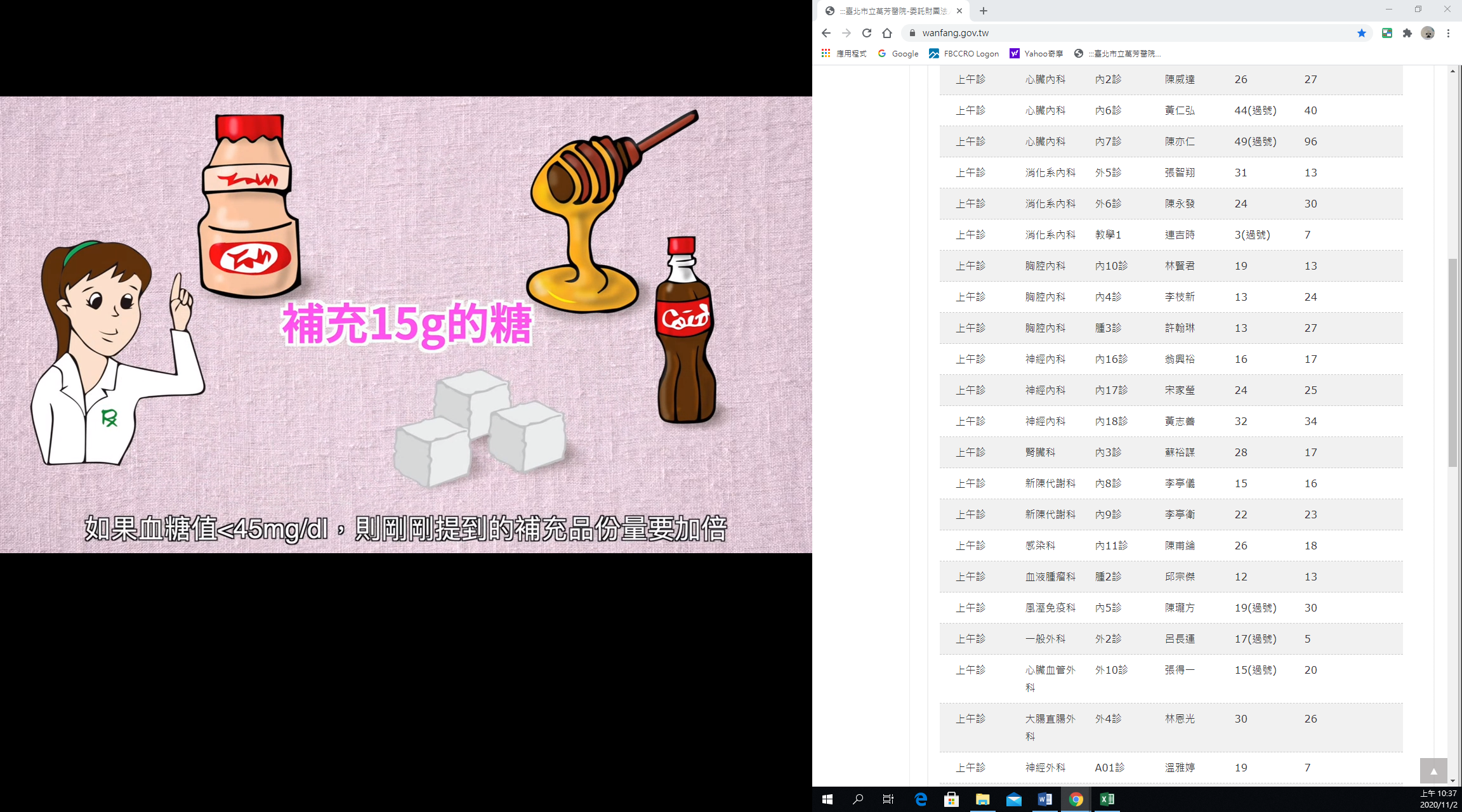 | Neuropathy  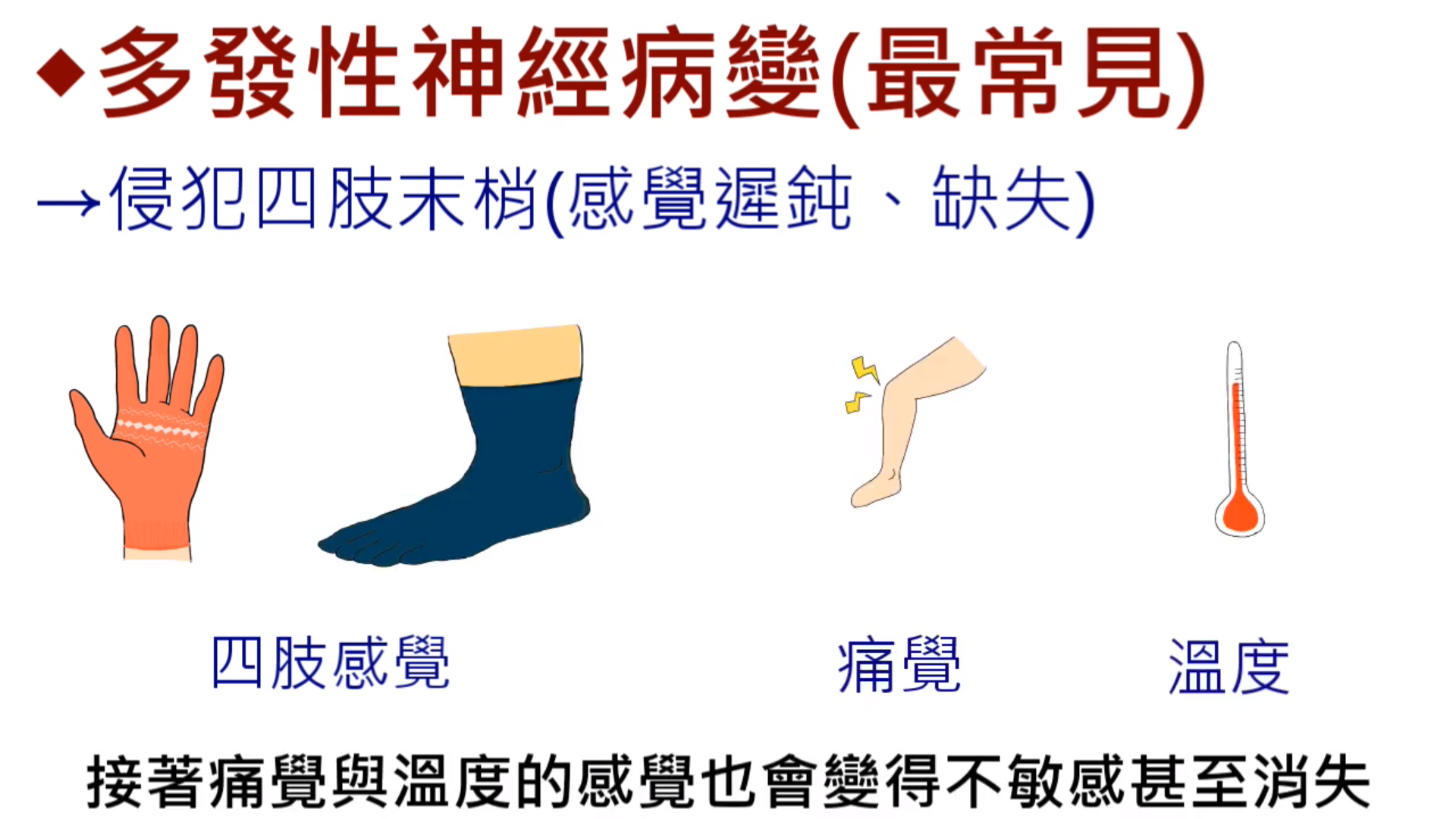 | |
| Microangiopathy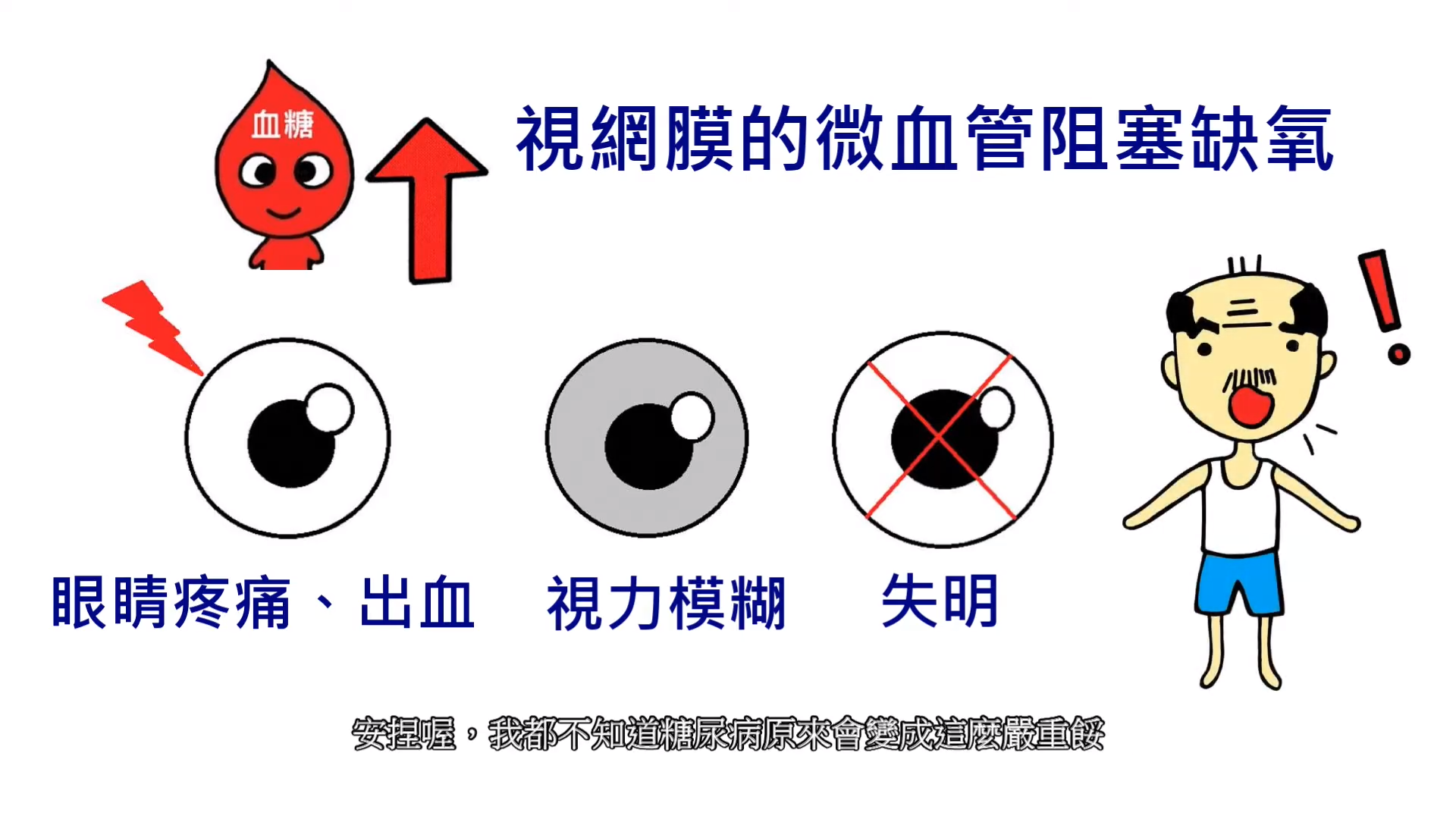 | Macroangiopathy 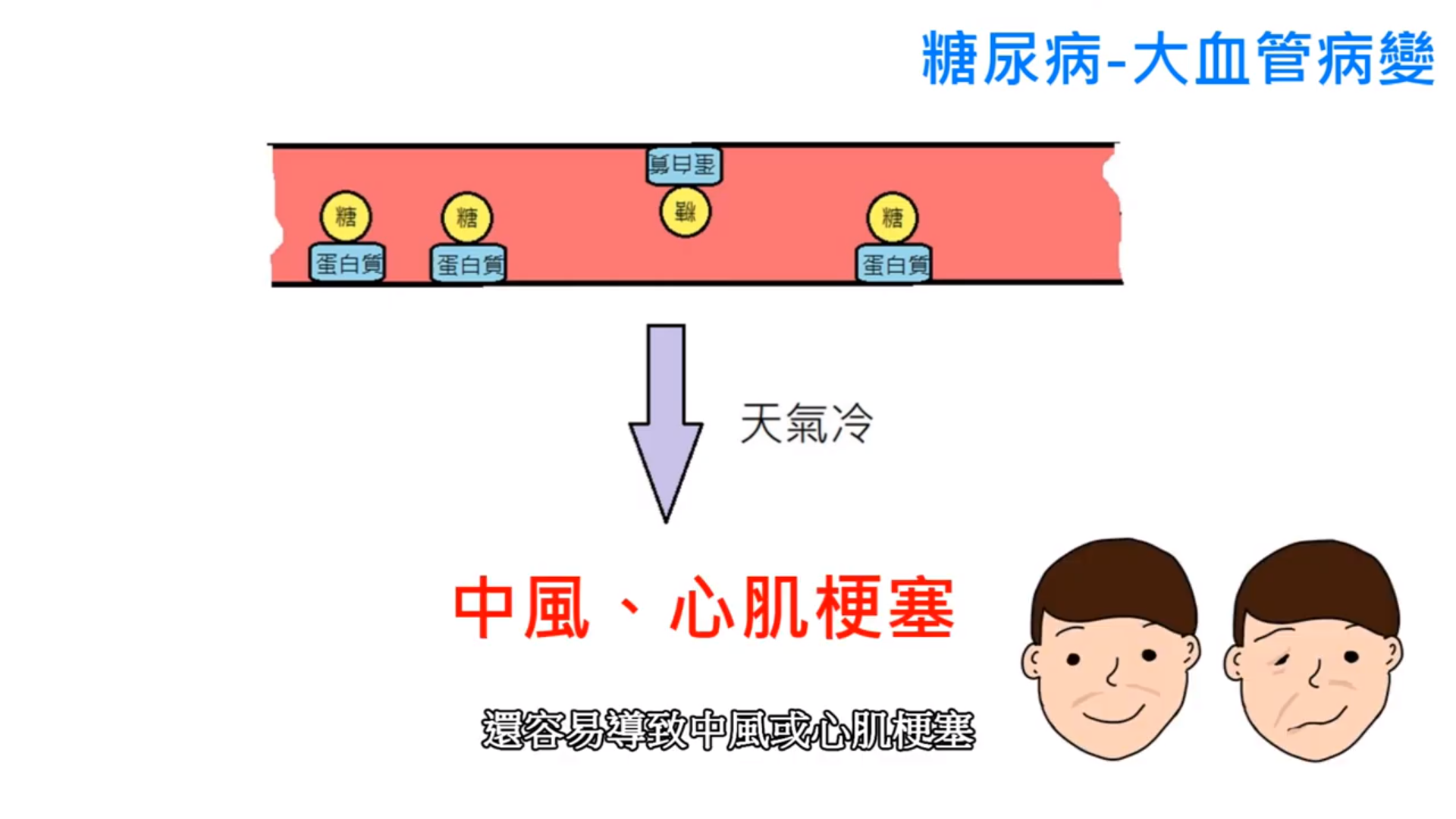 | |
| HbA1C  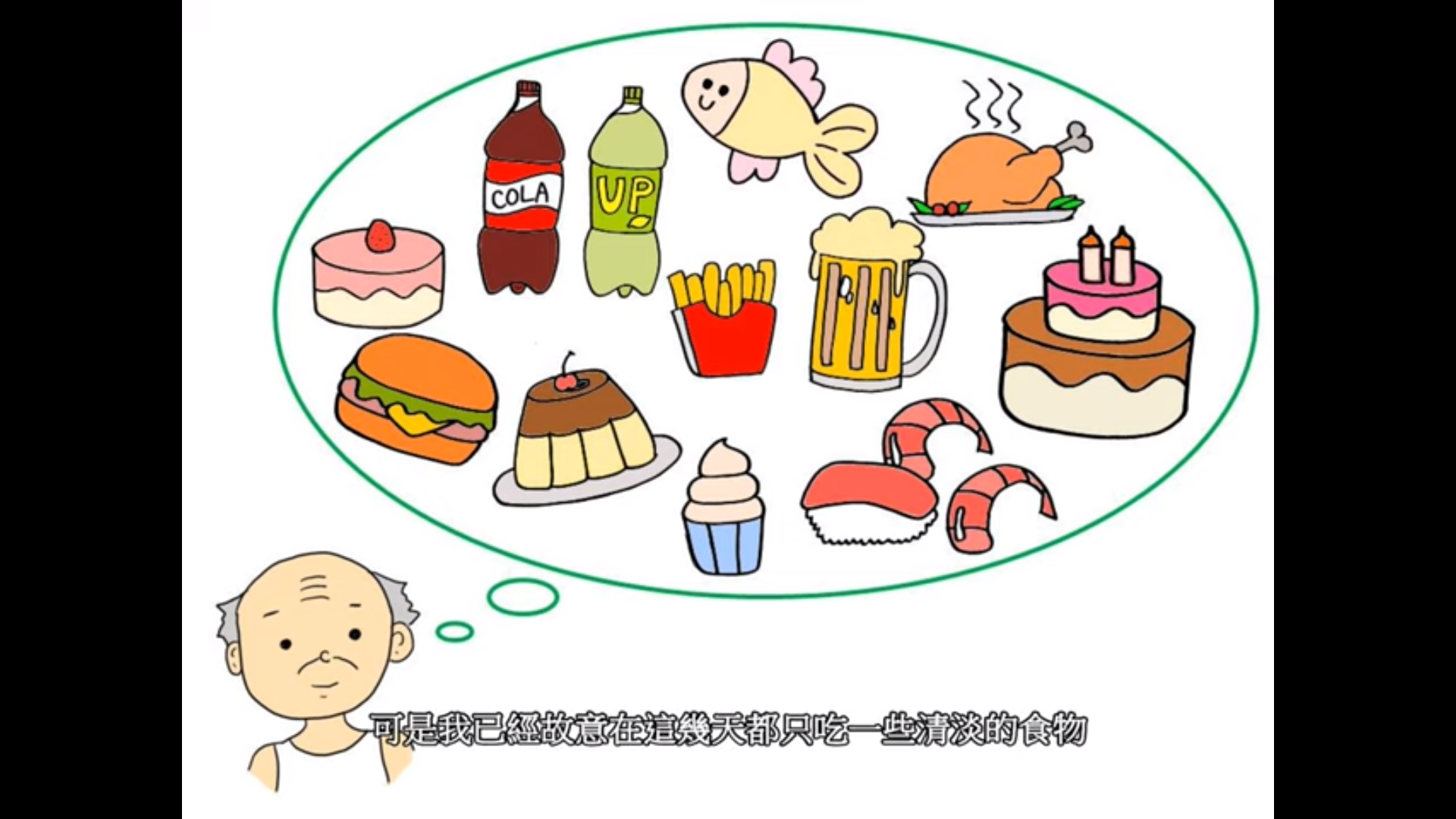 | Symptoms of early diabetes  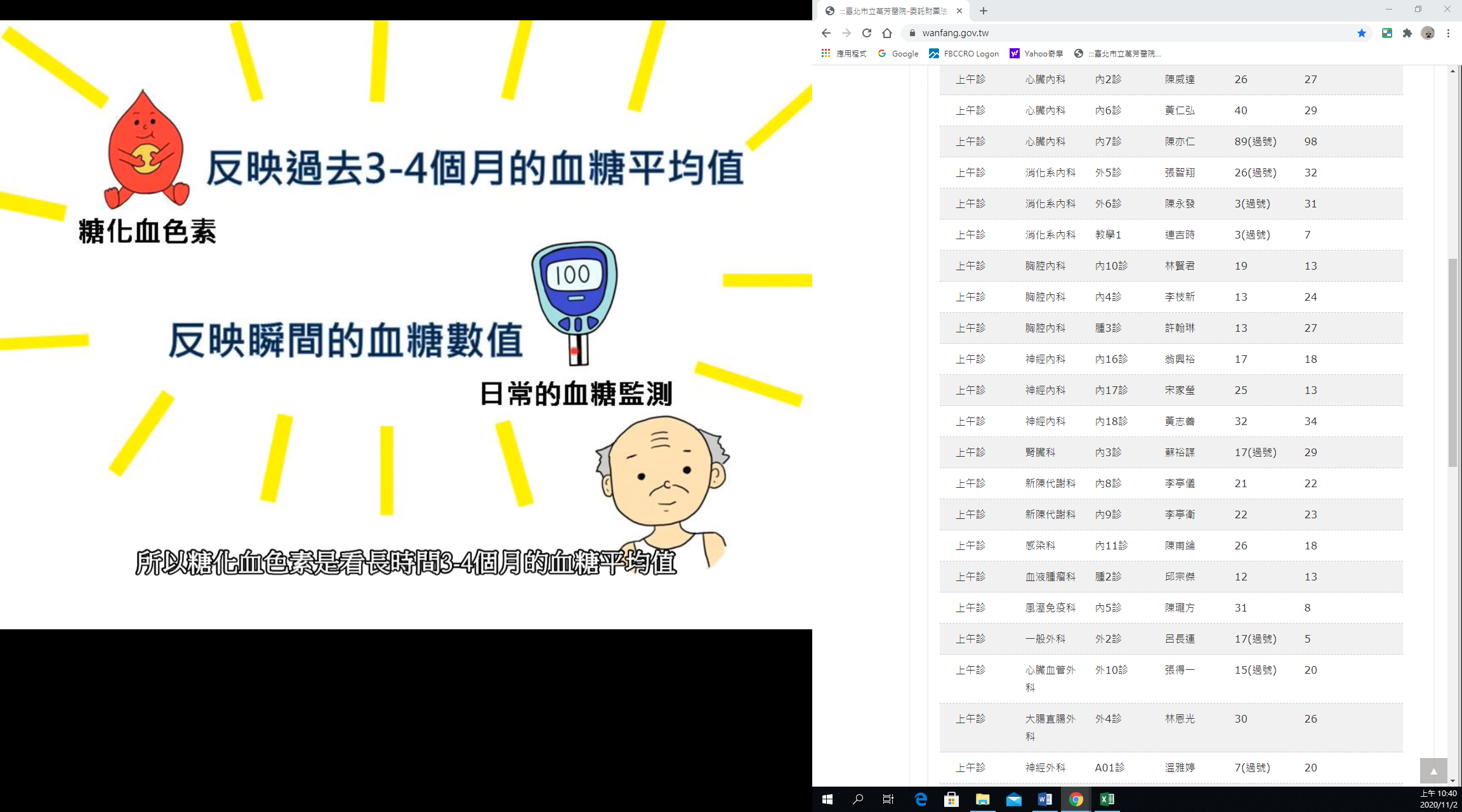 | |
| **Daily care** | | |
| Regular monitoring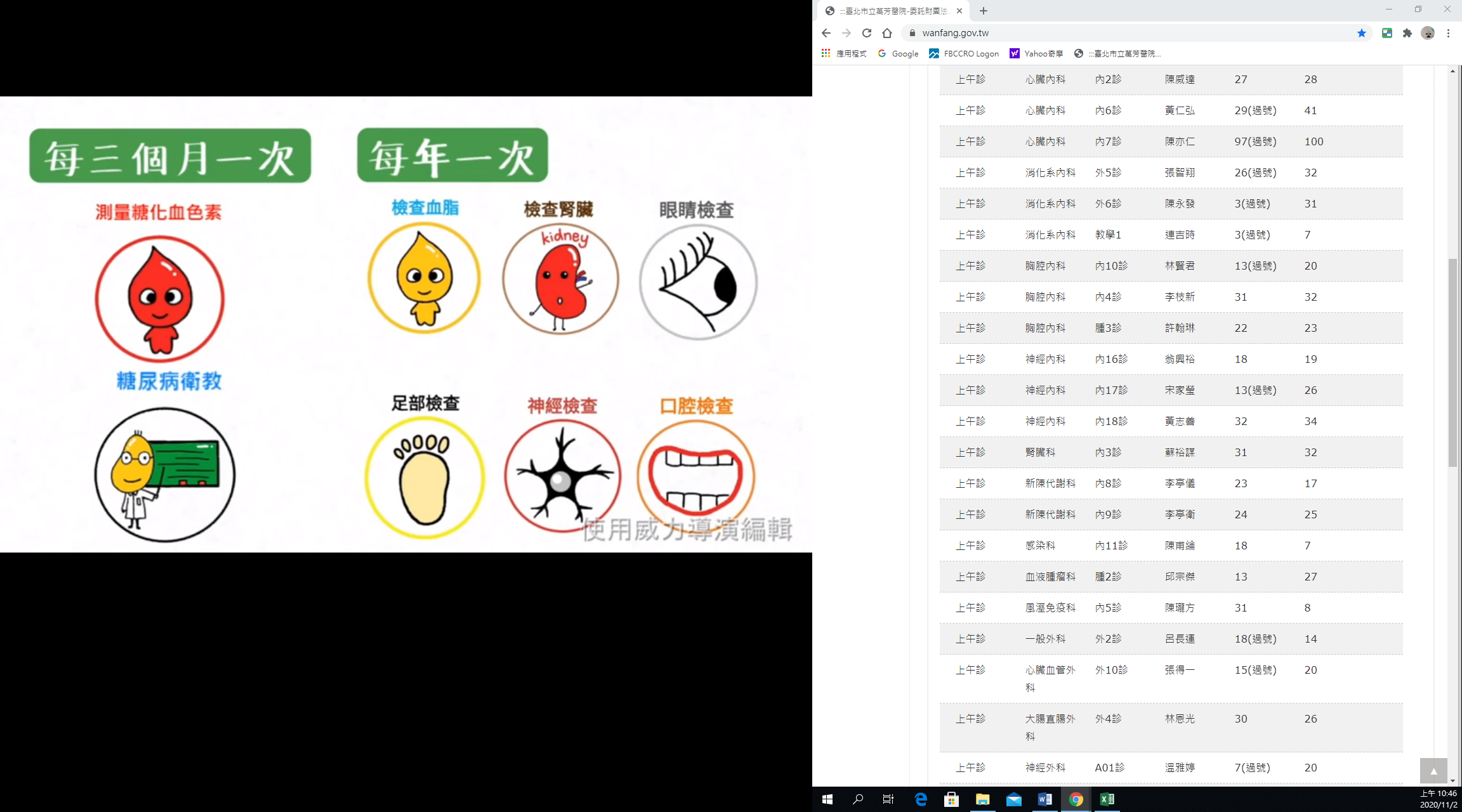 | Wound care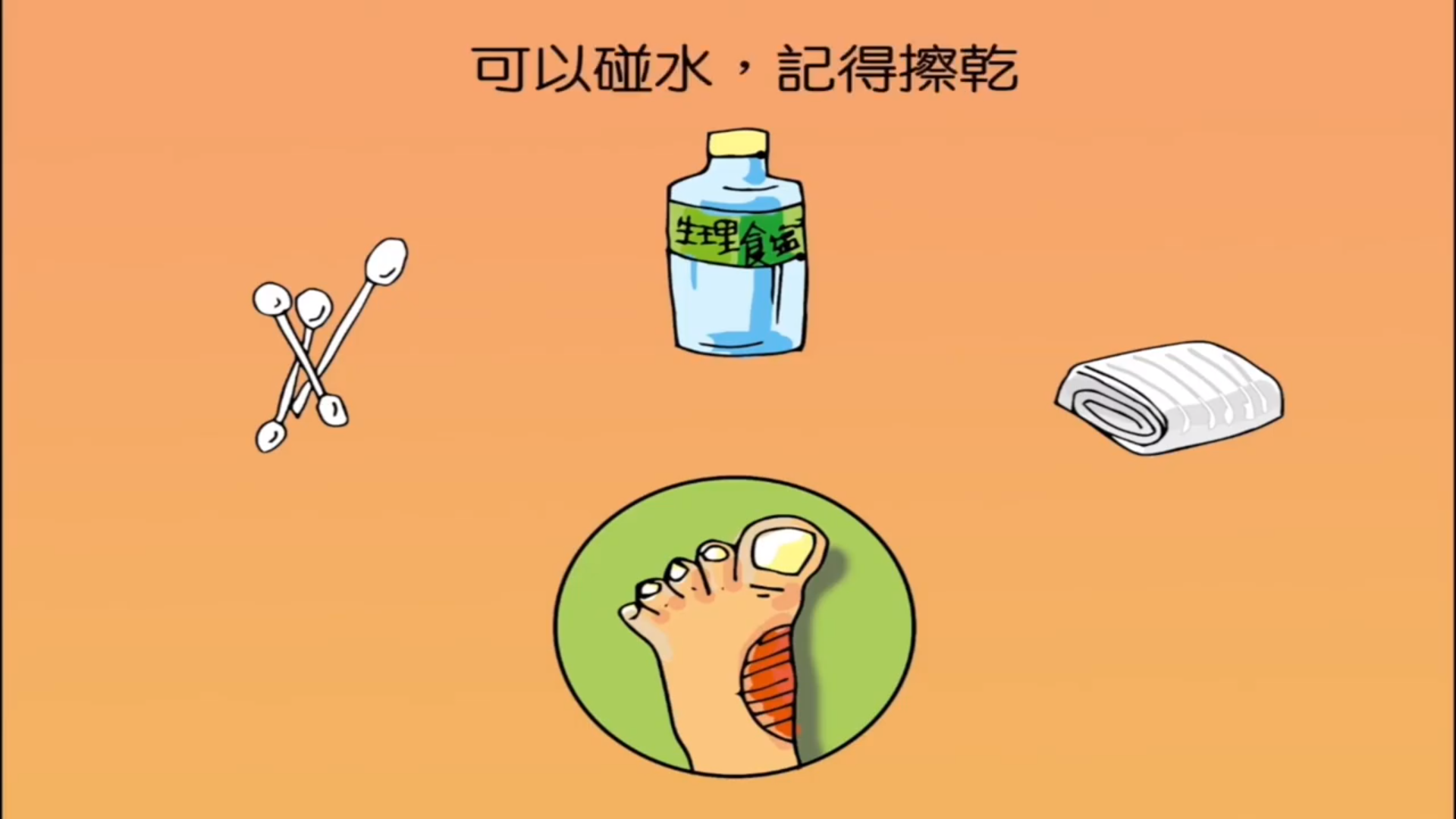 | |
| Precaution for hot springs  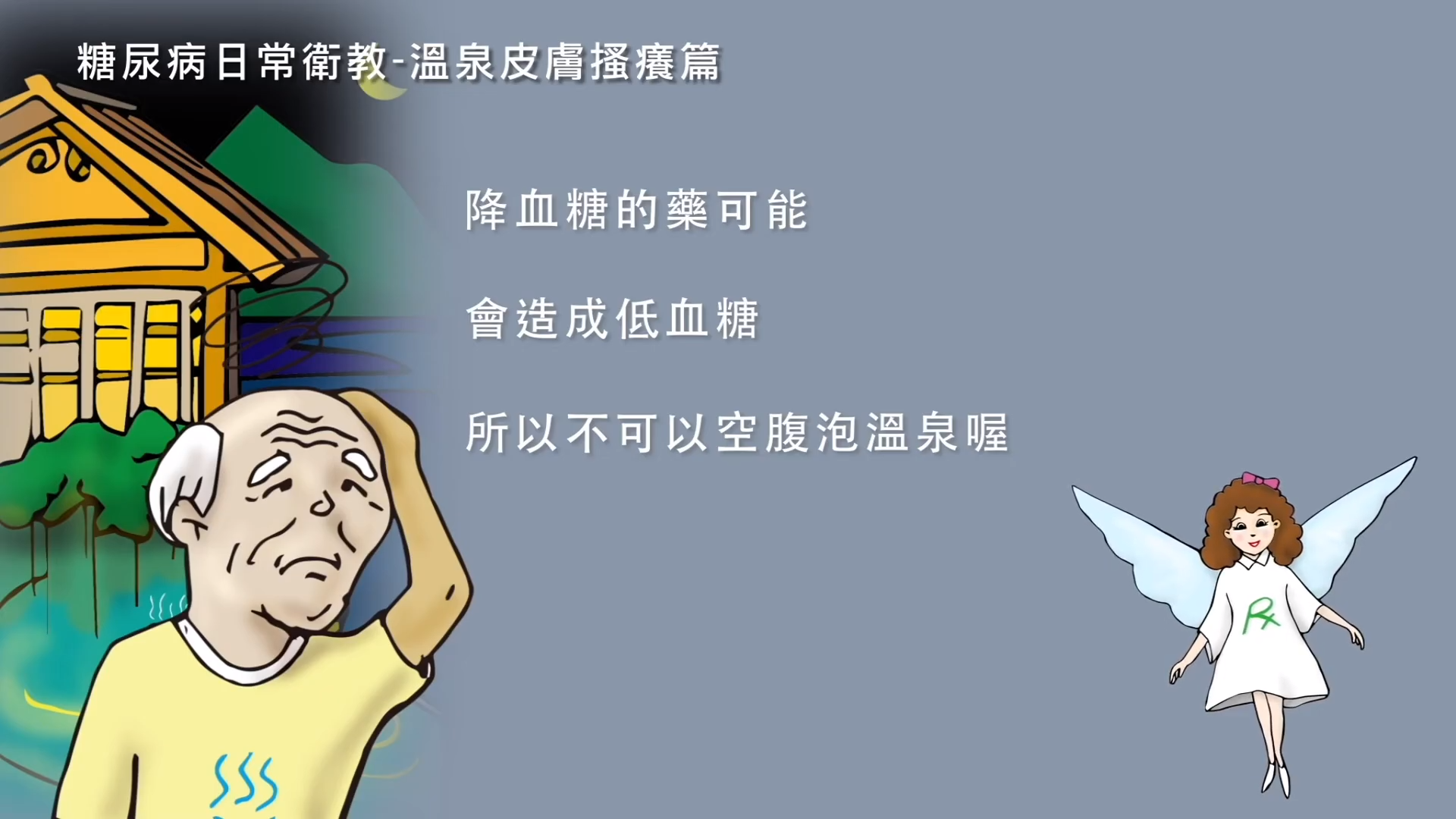 | Exercise  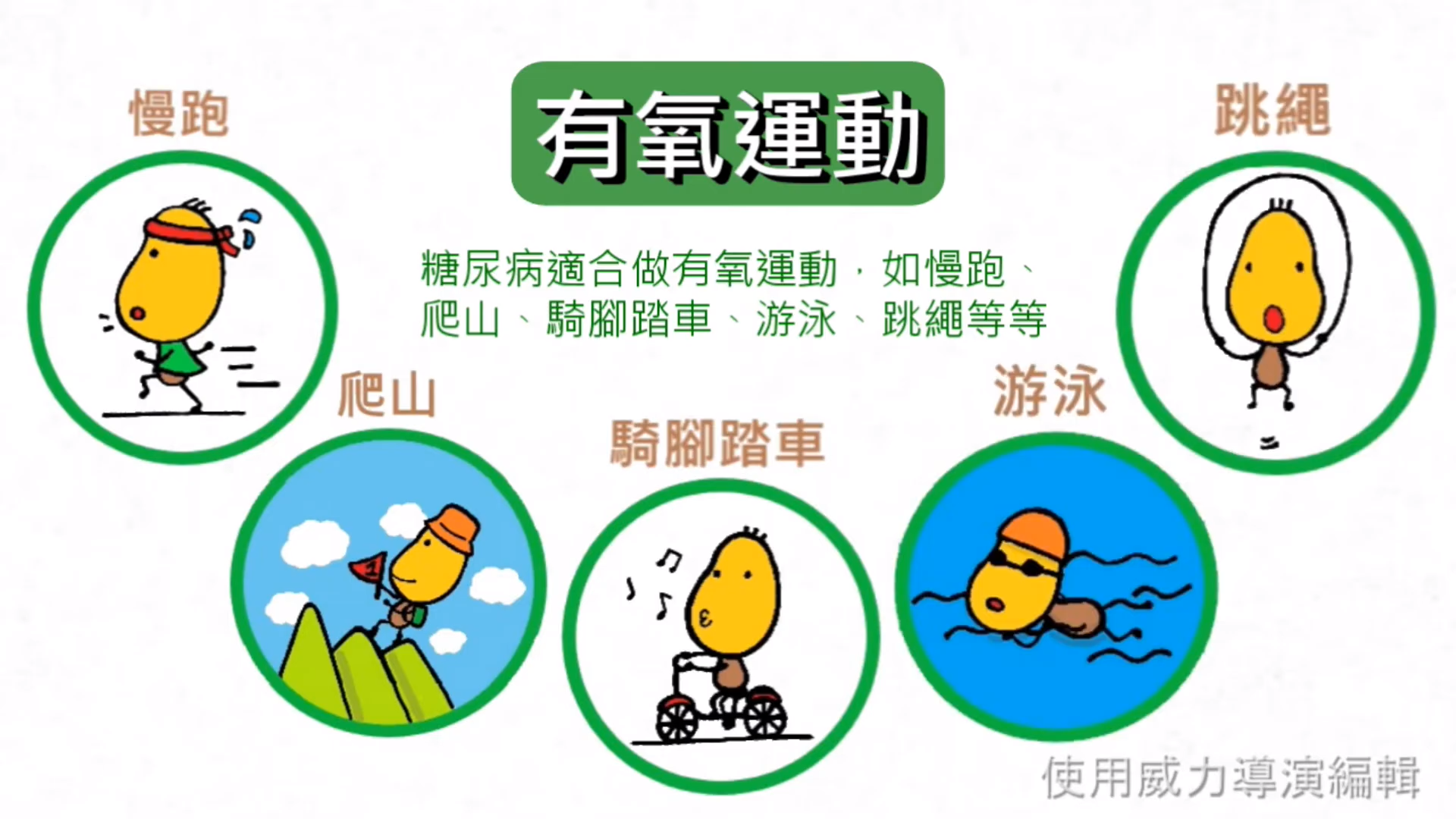 | |
| Blood glucose monitoring 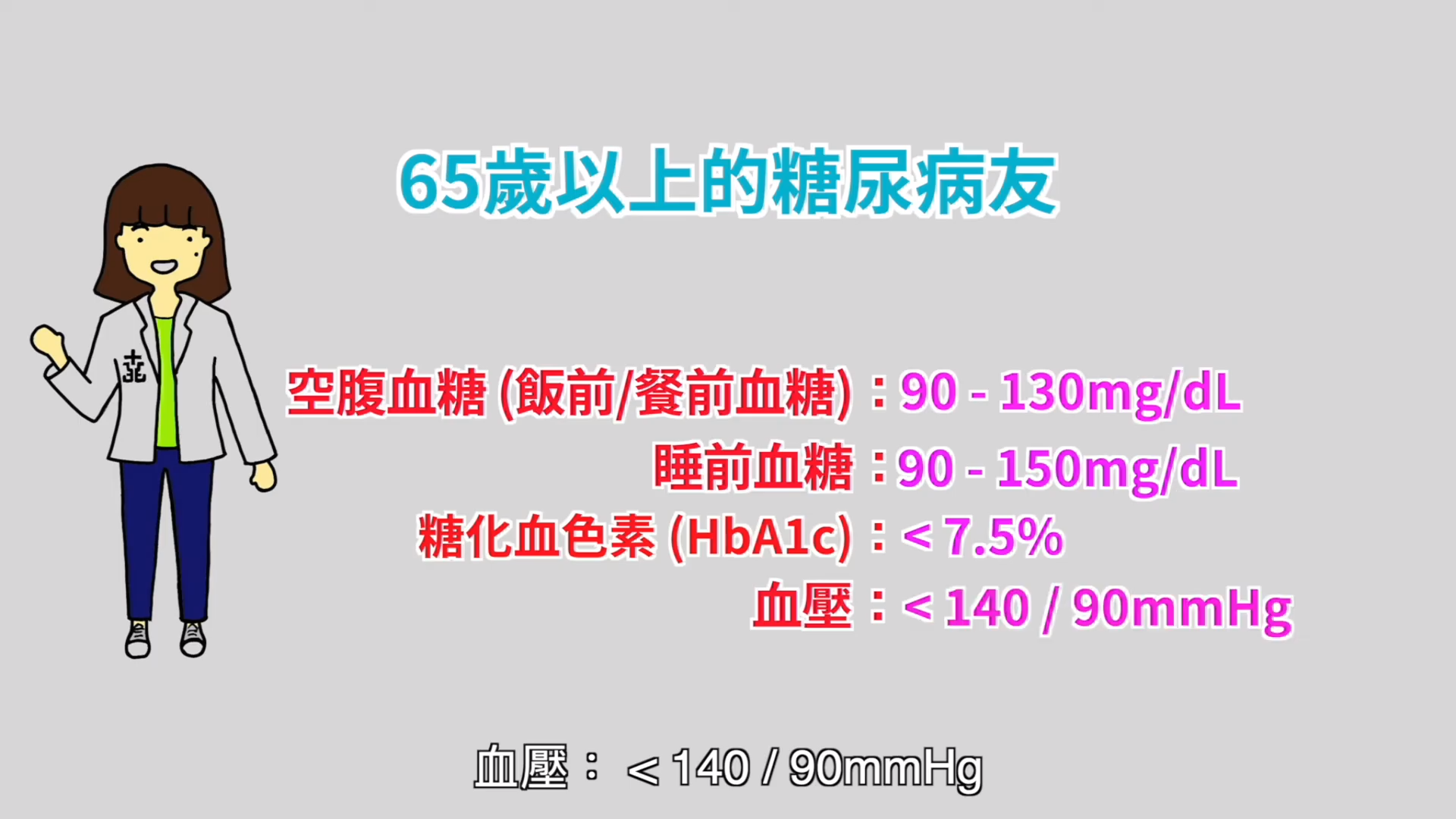 | Mosquito bites 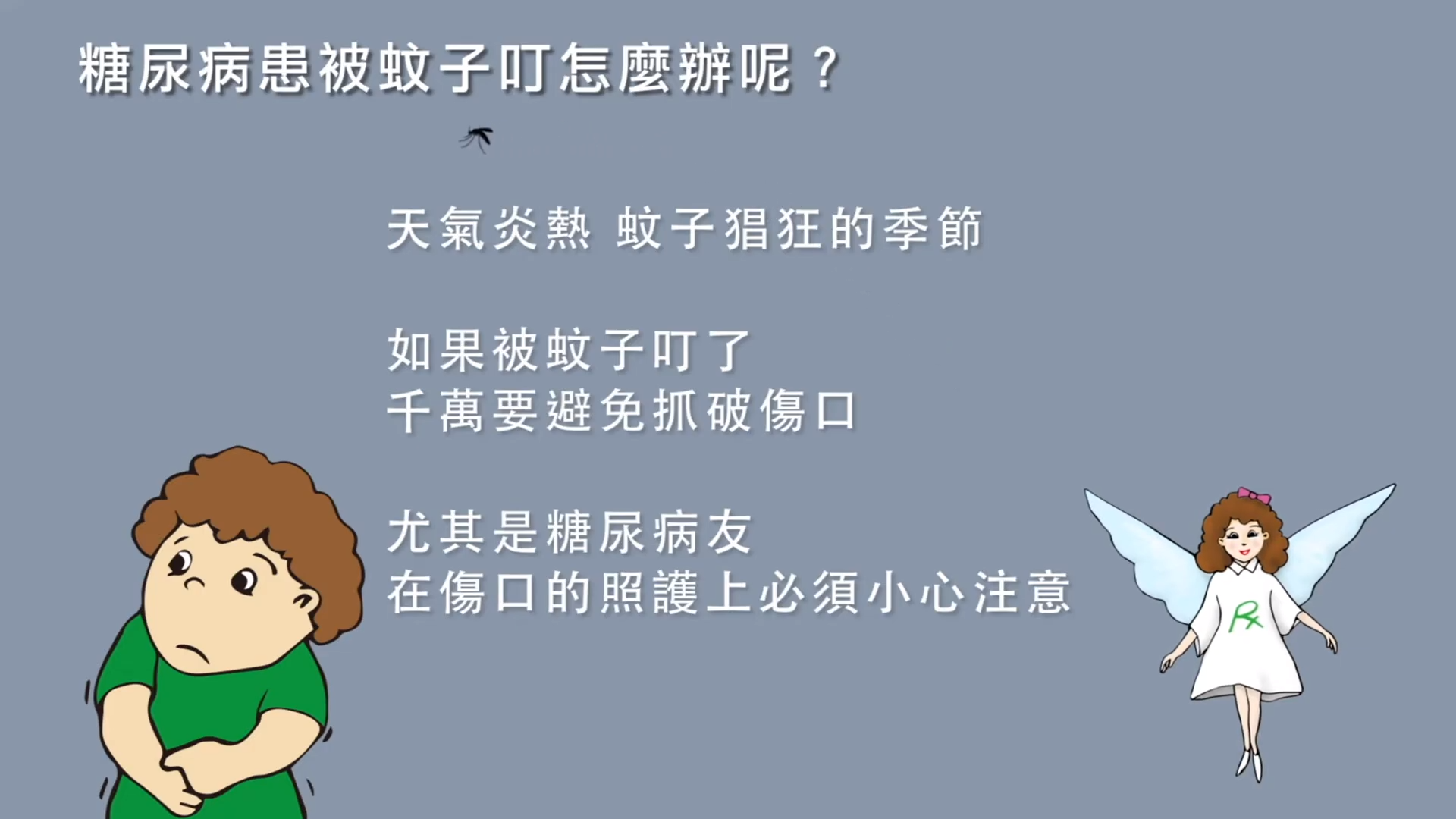 | |
| Quit smoking I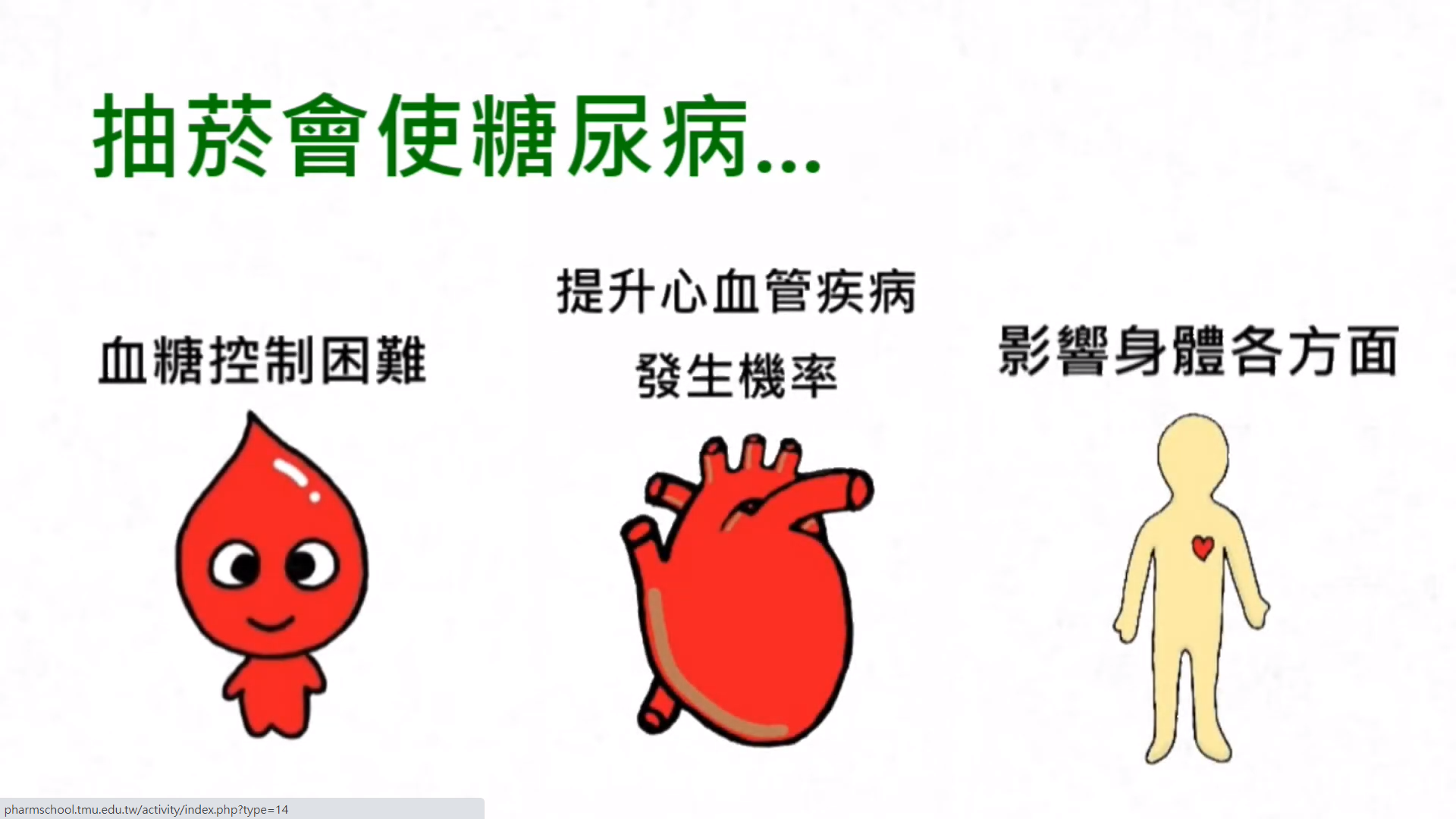 | Quit smoking II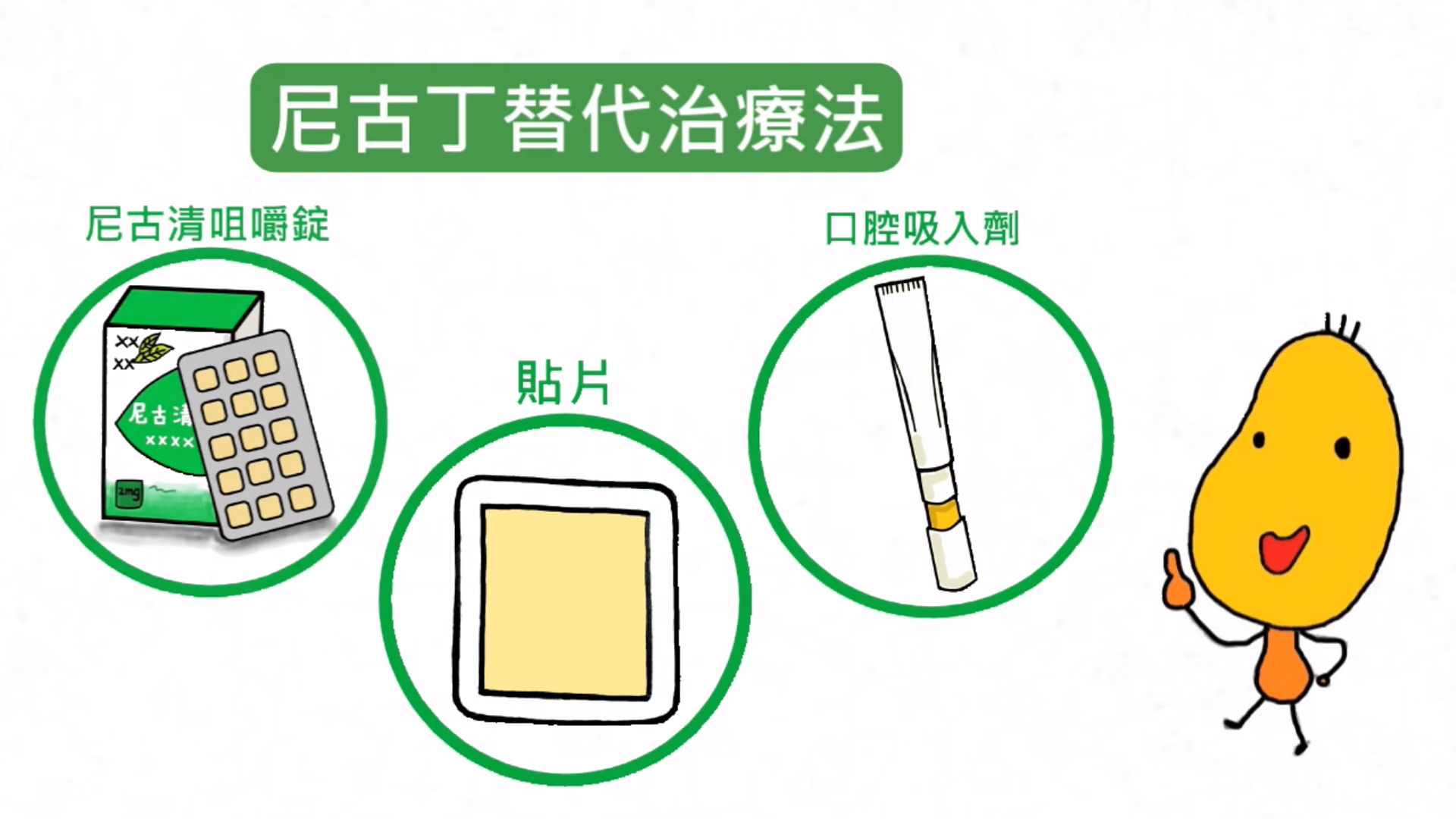 | |
| Quit smoking III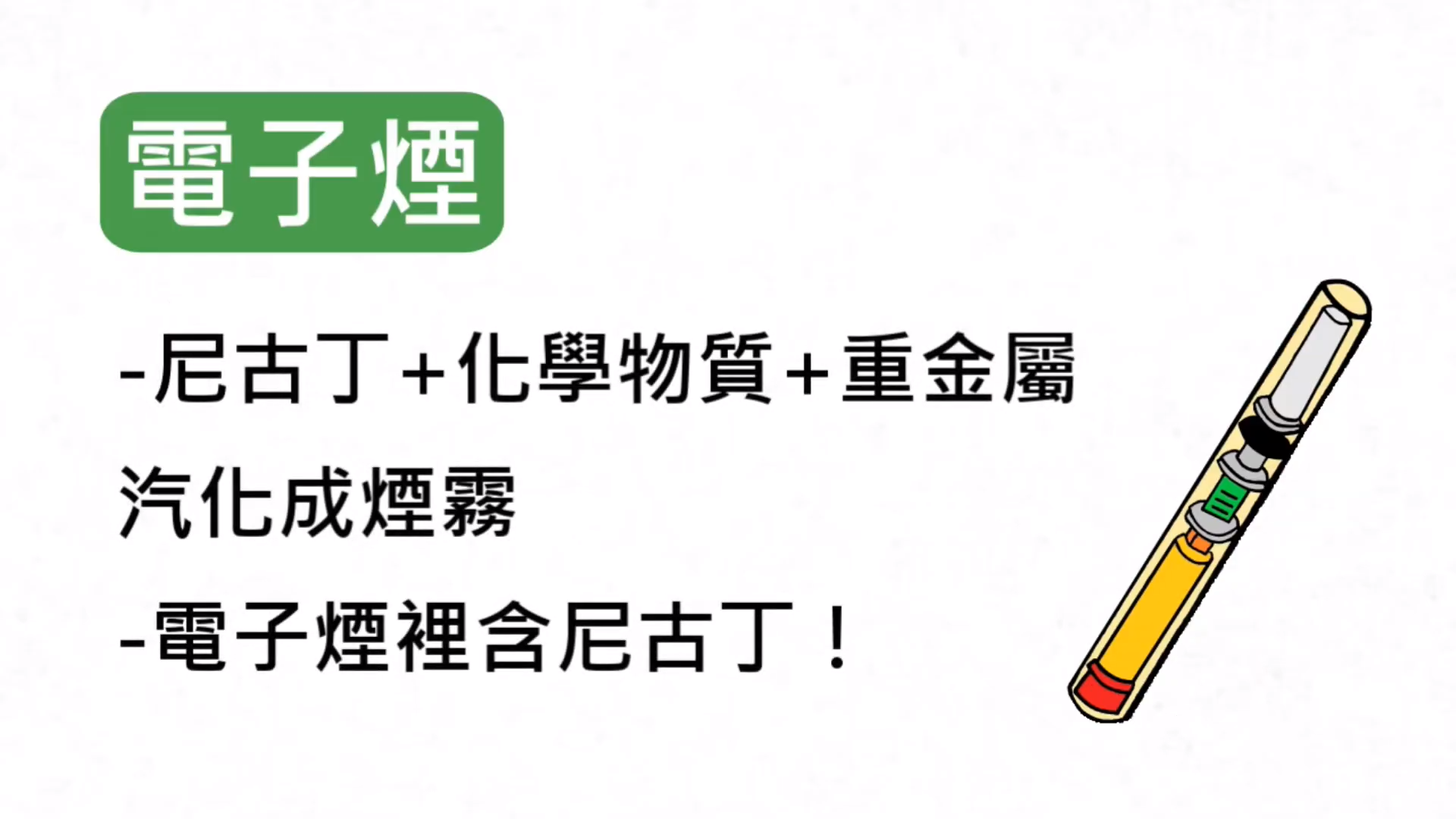 | Foot care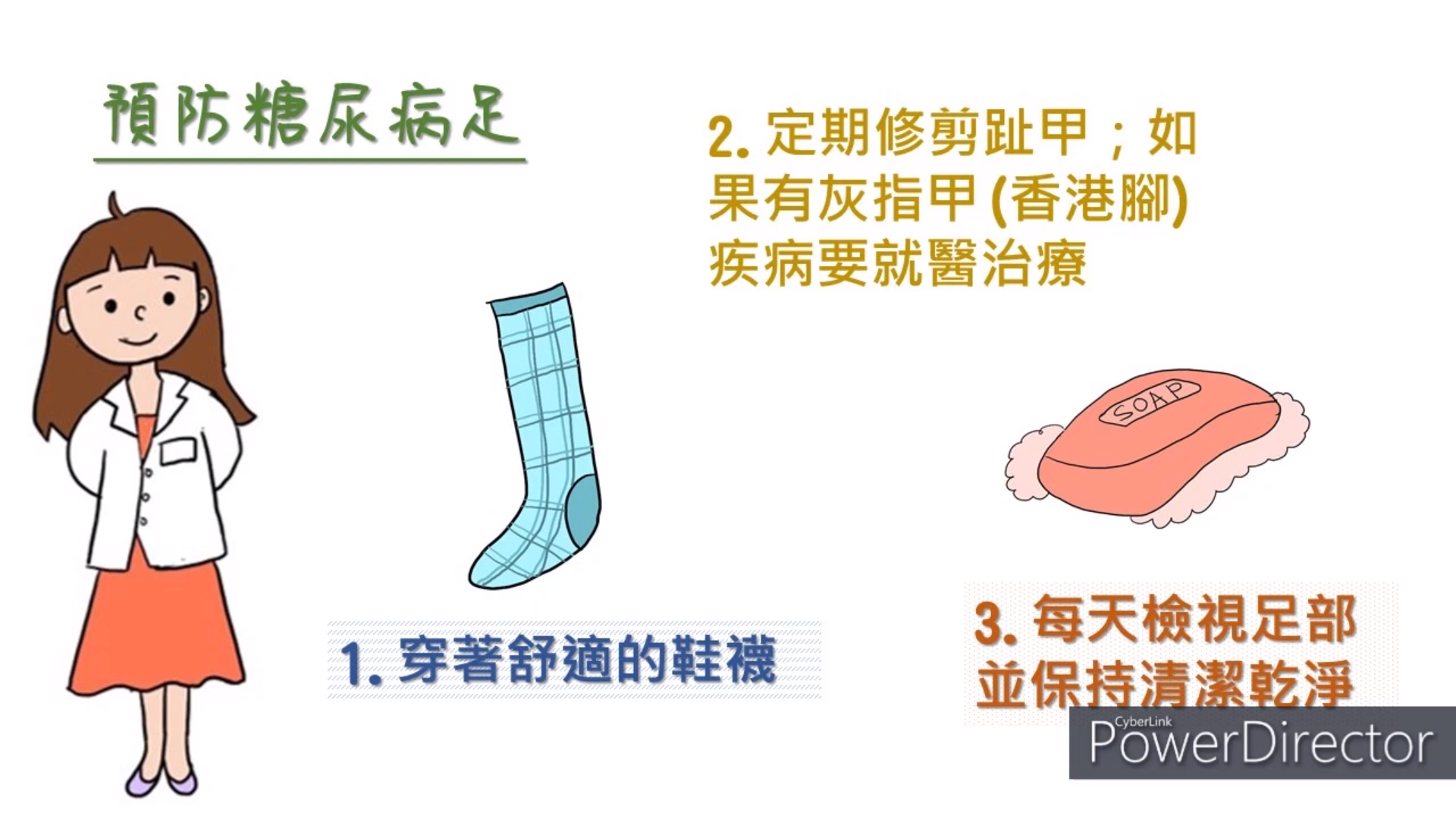 | |
| **Nutrition care** | | |
| Dietary principle 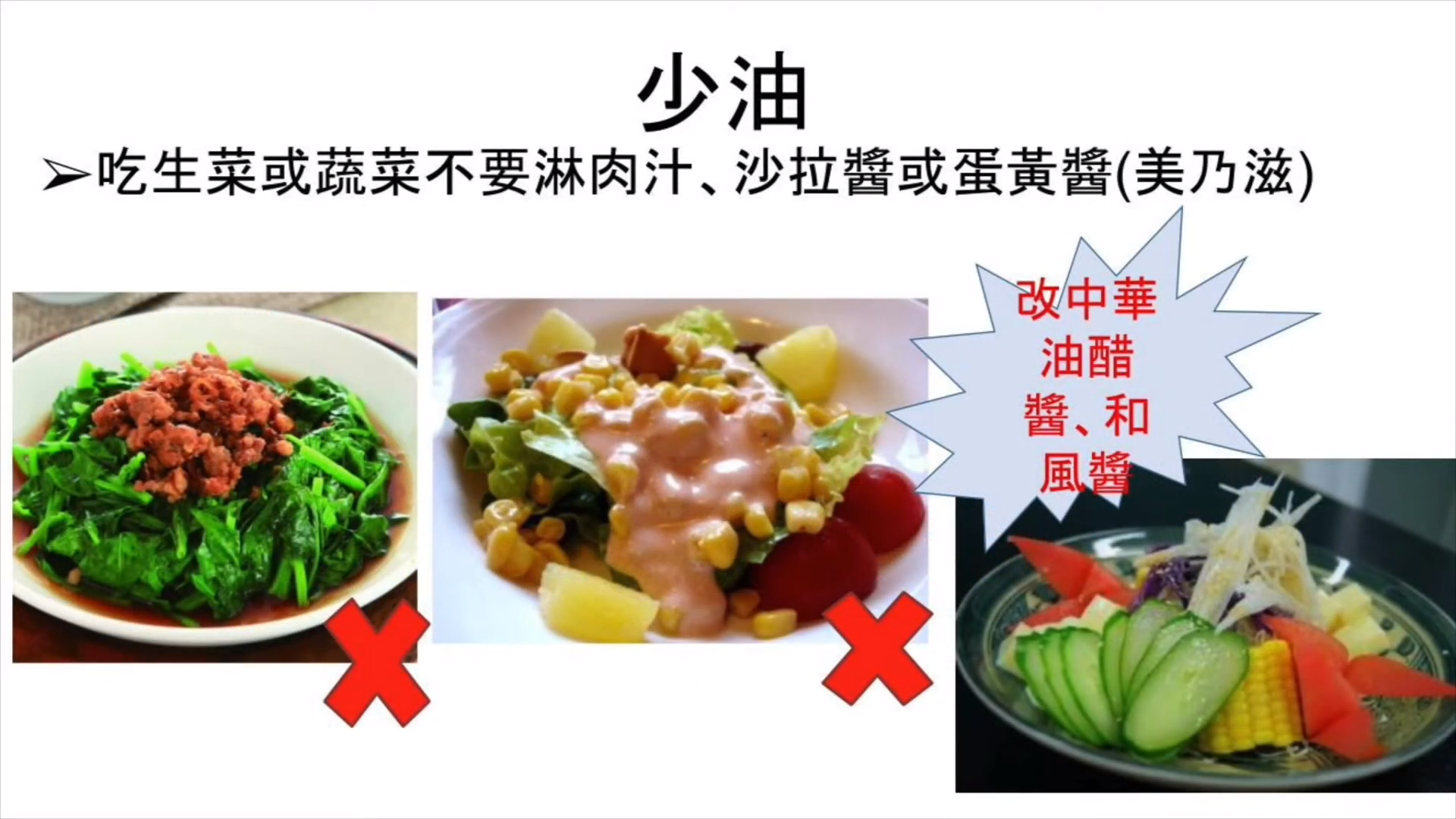 | Balanced diet 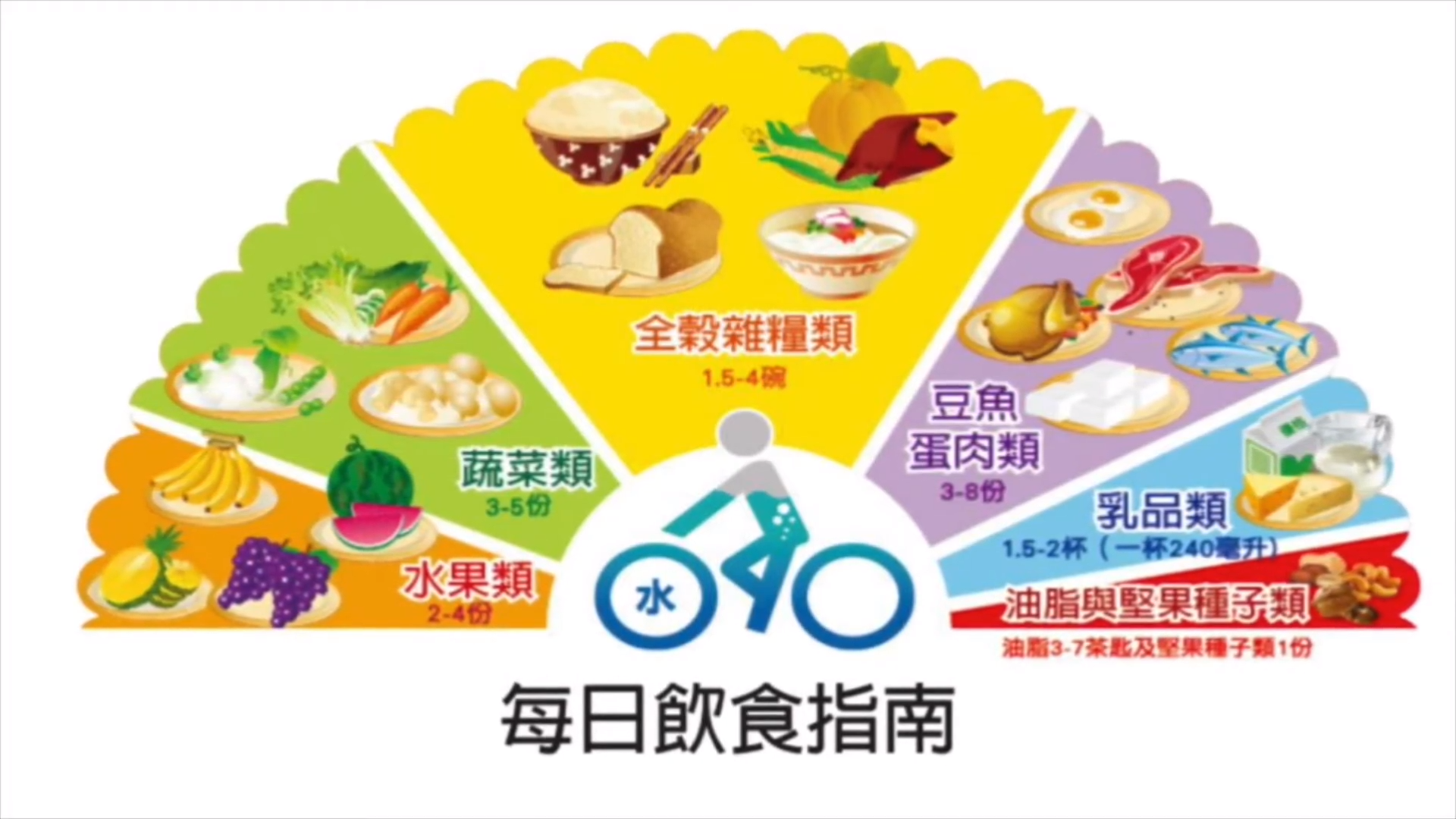 | |
| Homemade - celery salad \|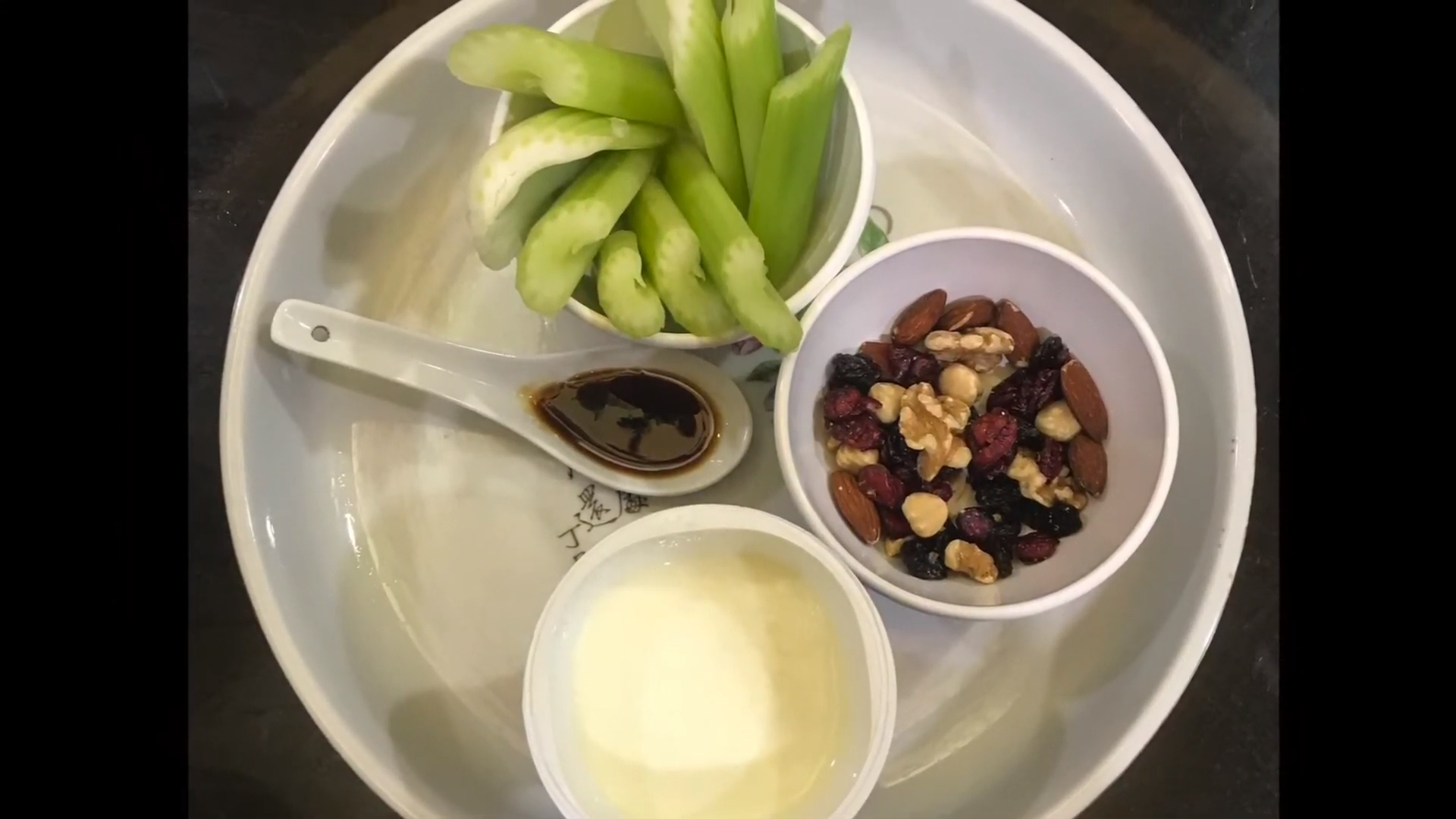 | Homemade - Chinese chickpea fungus soup 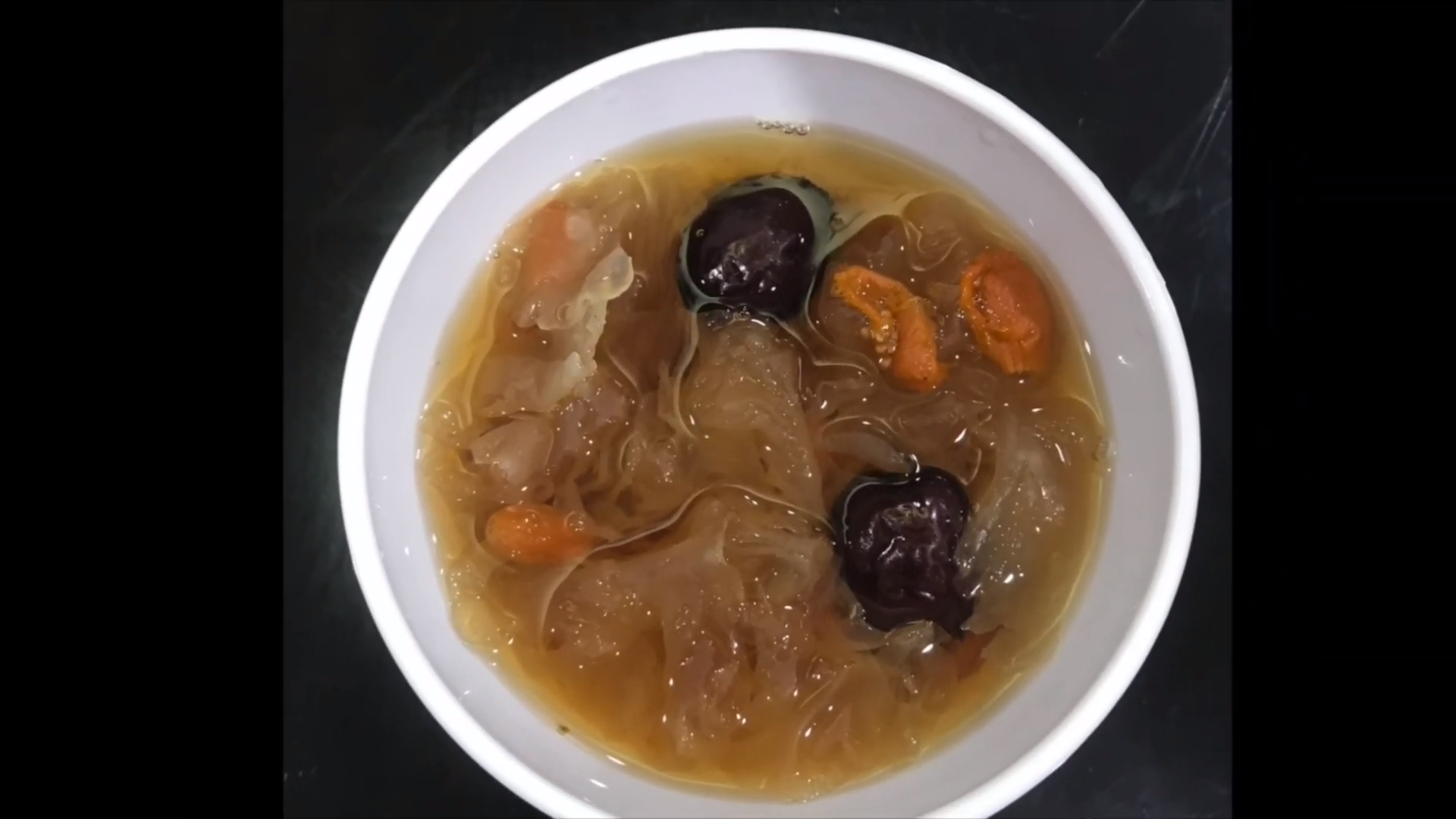 | |
| Carbohydrates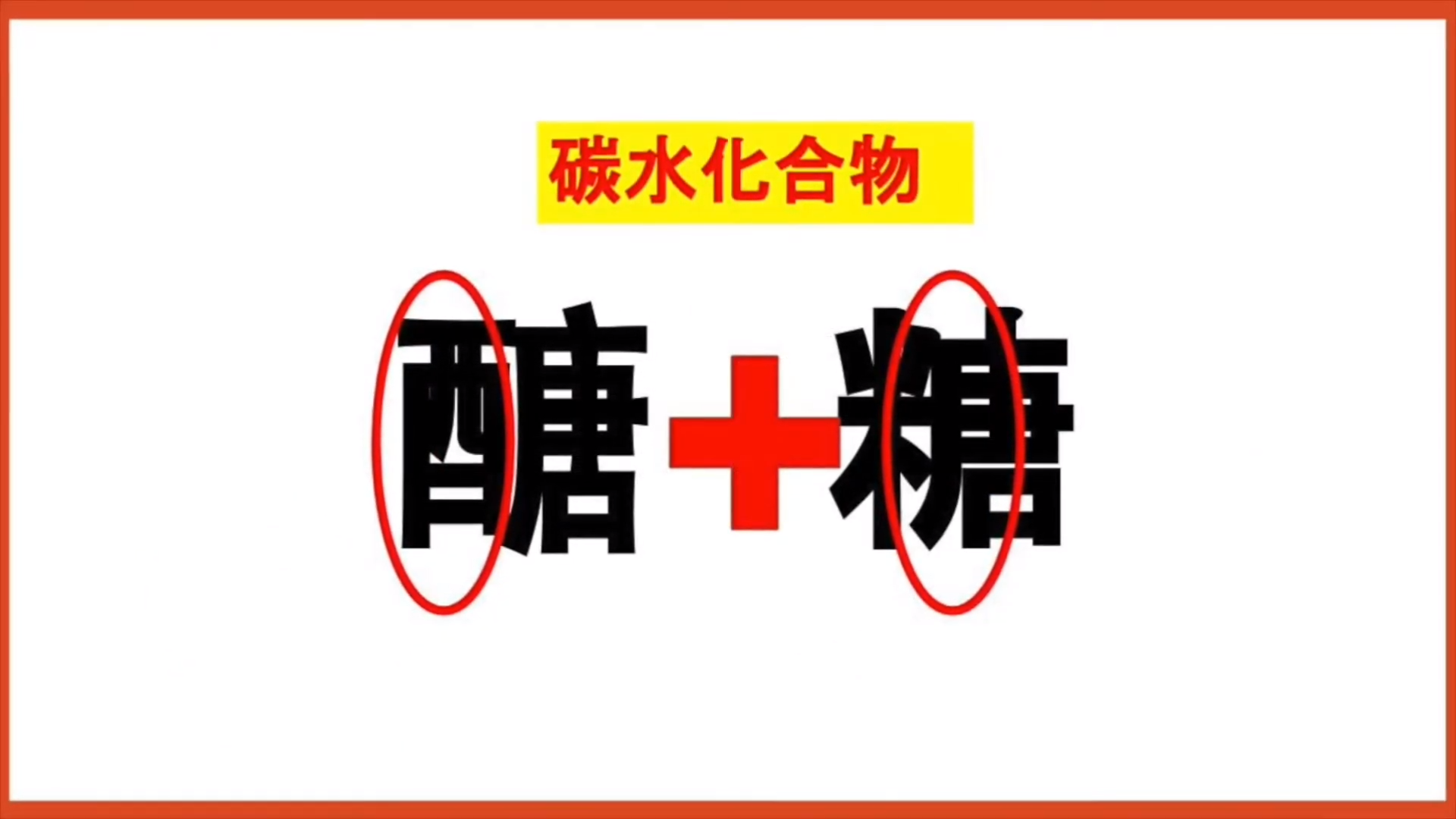 | Nutrition label  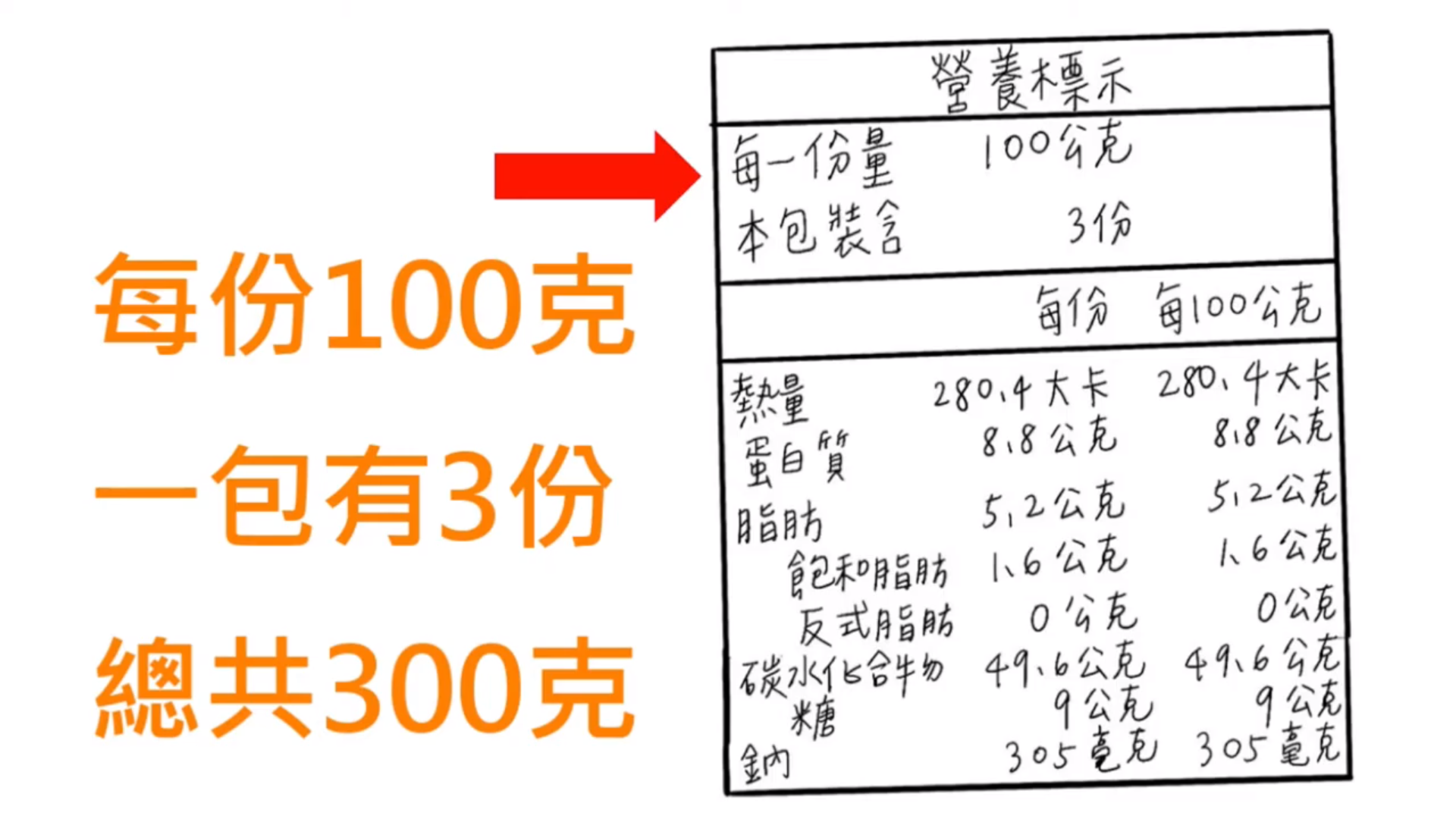 | |
| **Diabetes drugs** | | |
| Loditon/Uformin (Metformin) I  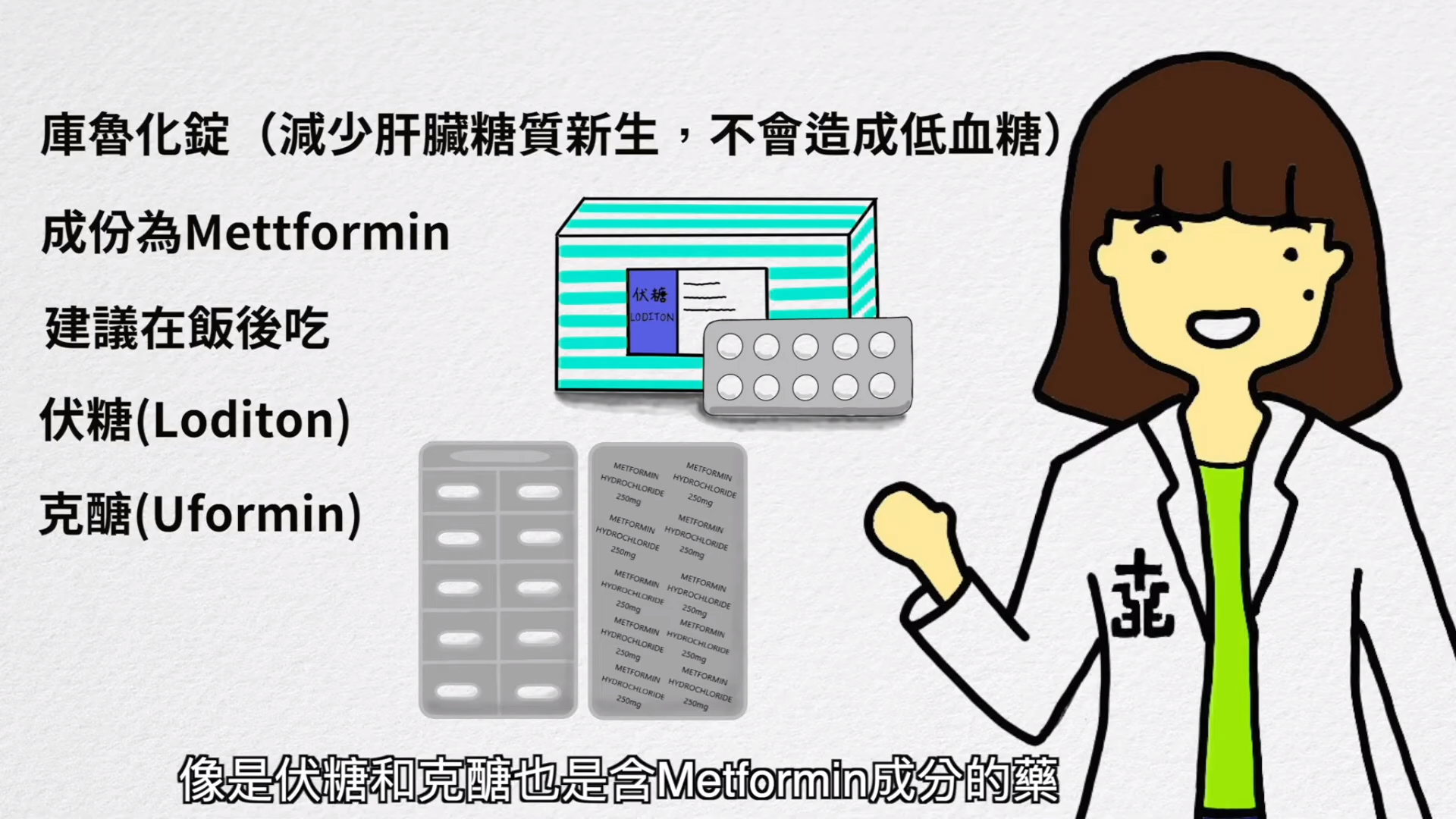 | | Loditon/Uformin (Metformin) II  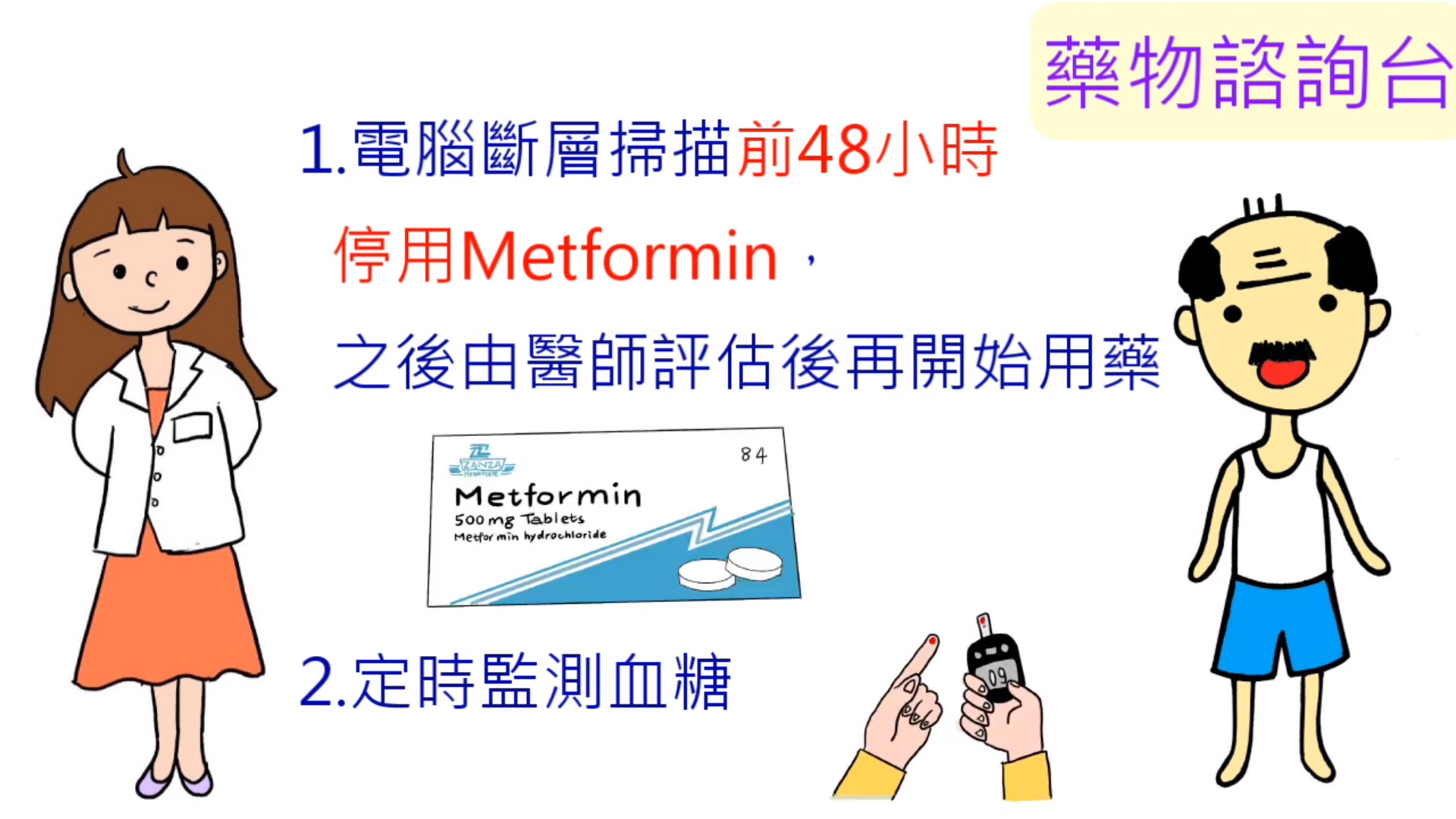 |
| Glucobay (Acarbose)  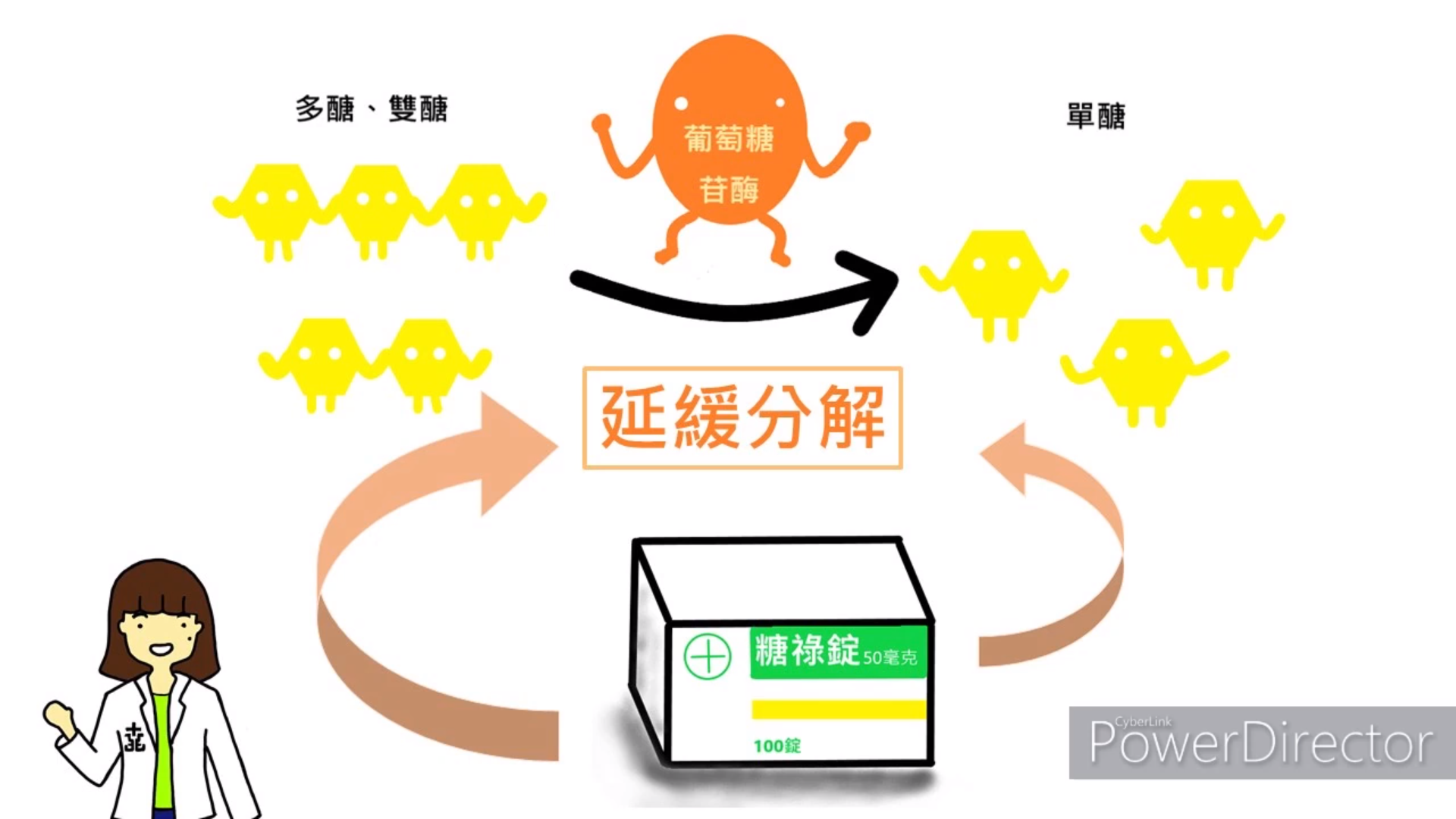 | | Galvus (Vildagliptin)  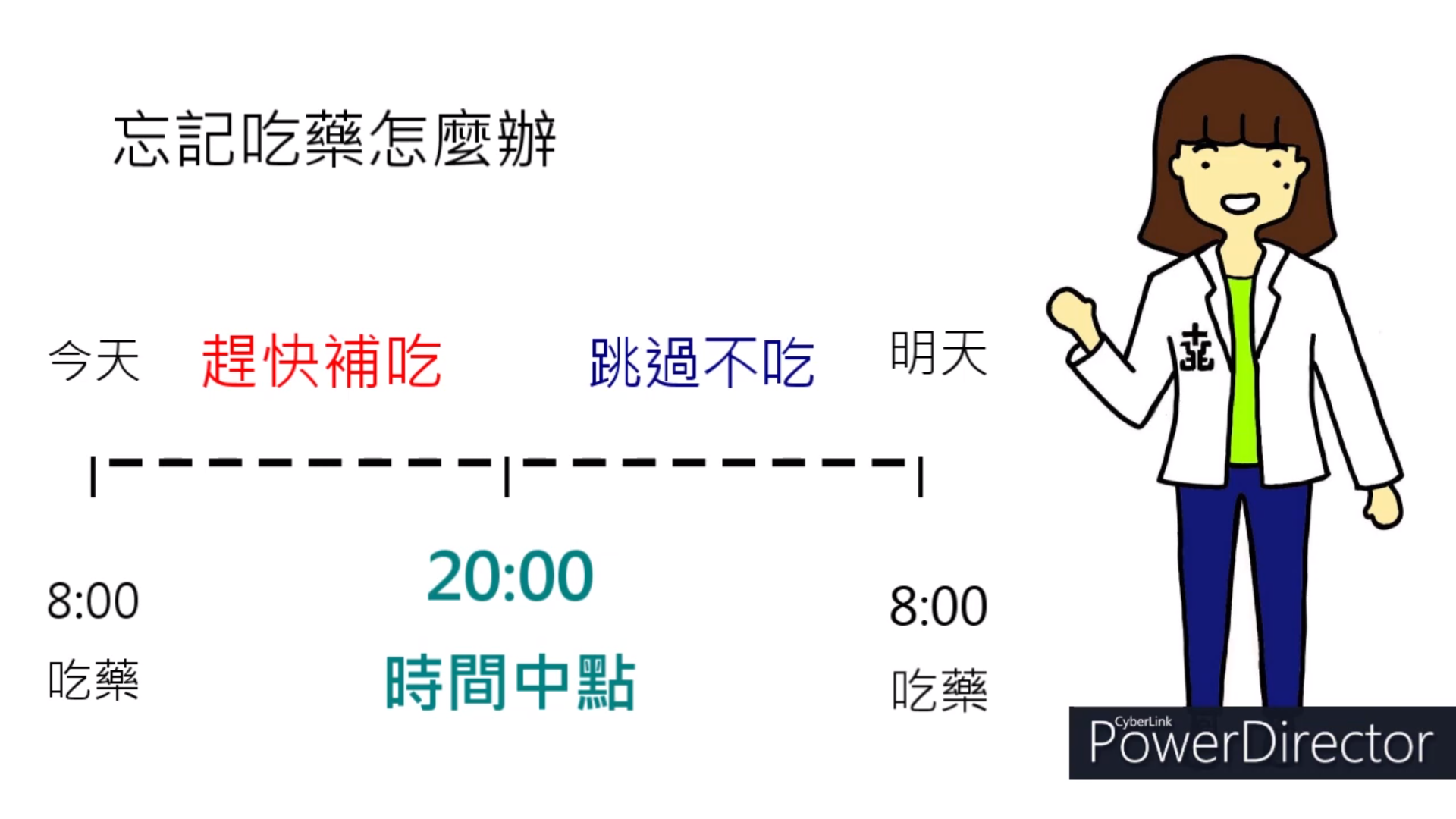 |
| Trajenta (Linagliptin)  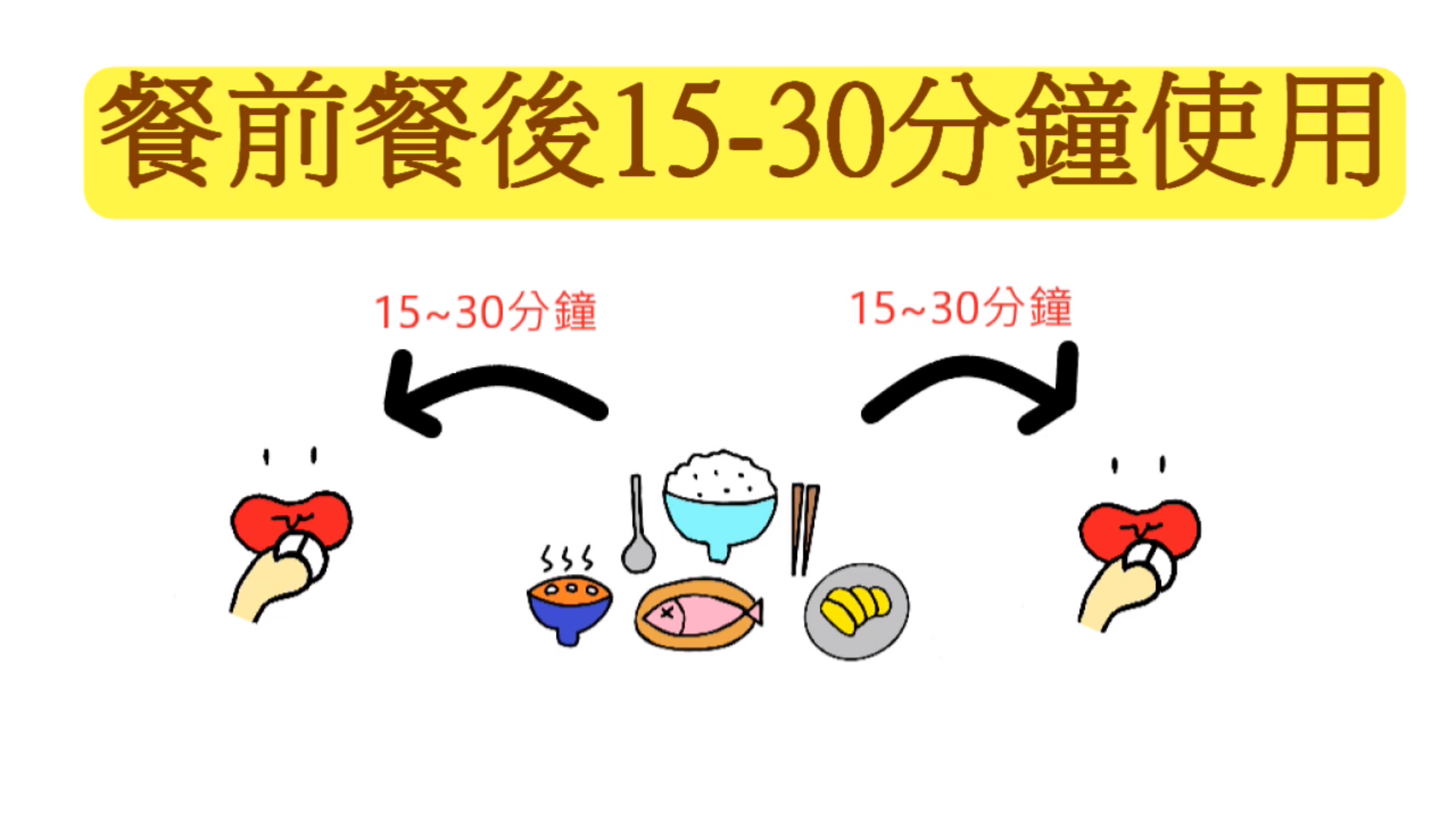 | | Januvia (Sitagliptin) 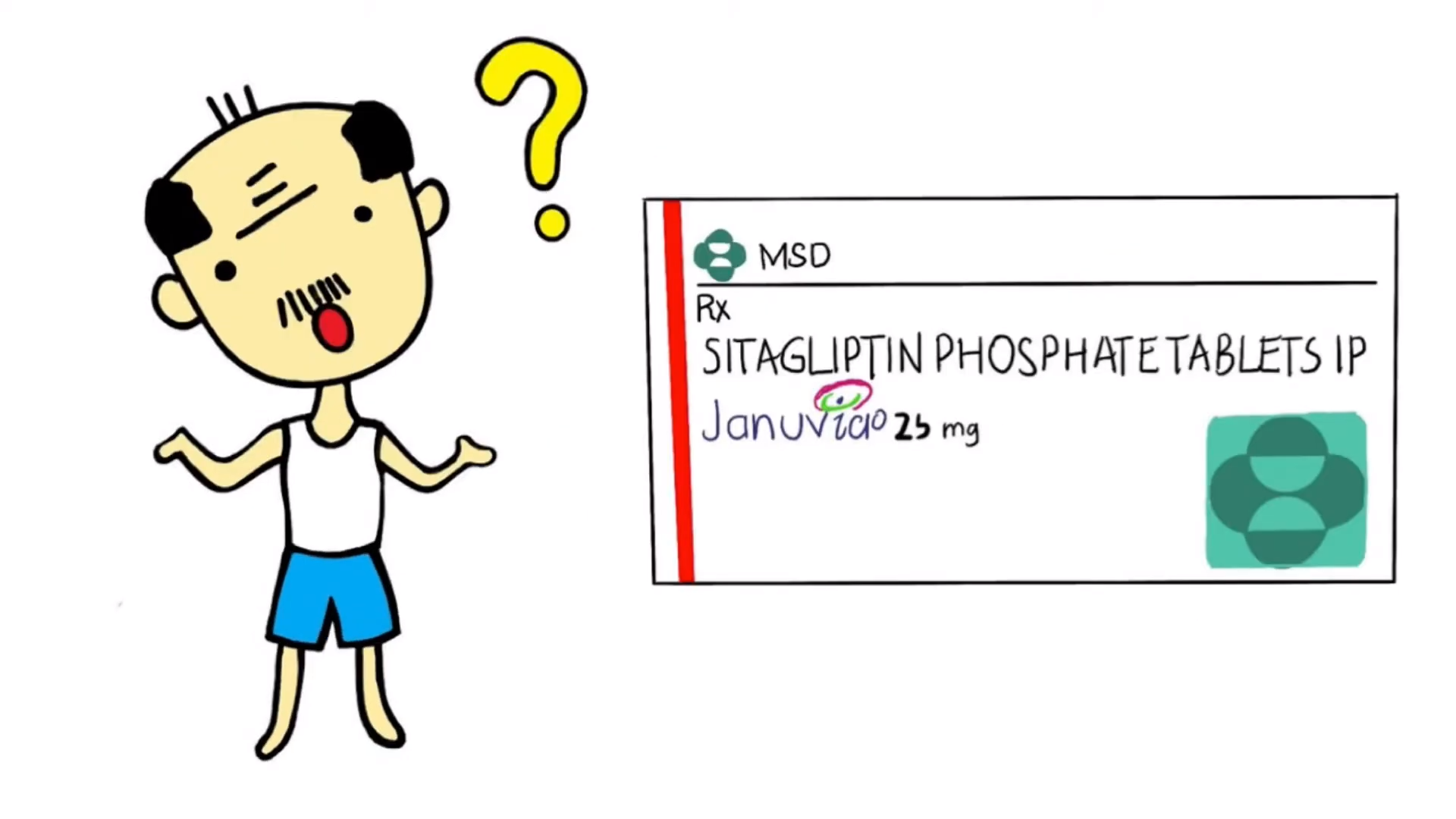 |
| Starlix (Nateglinide)  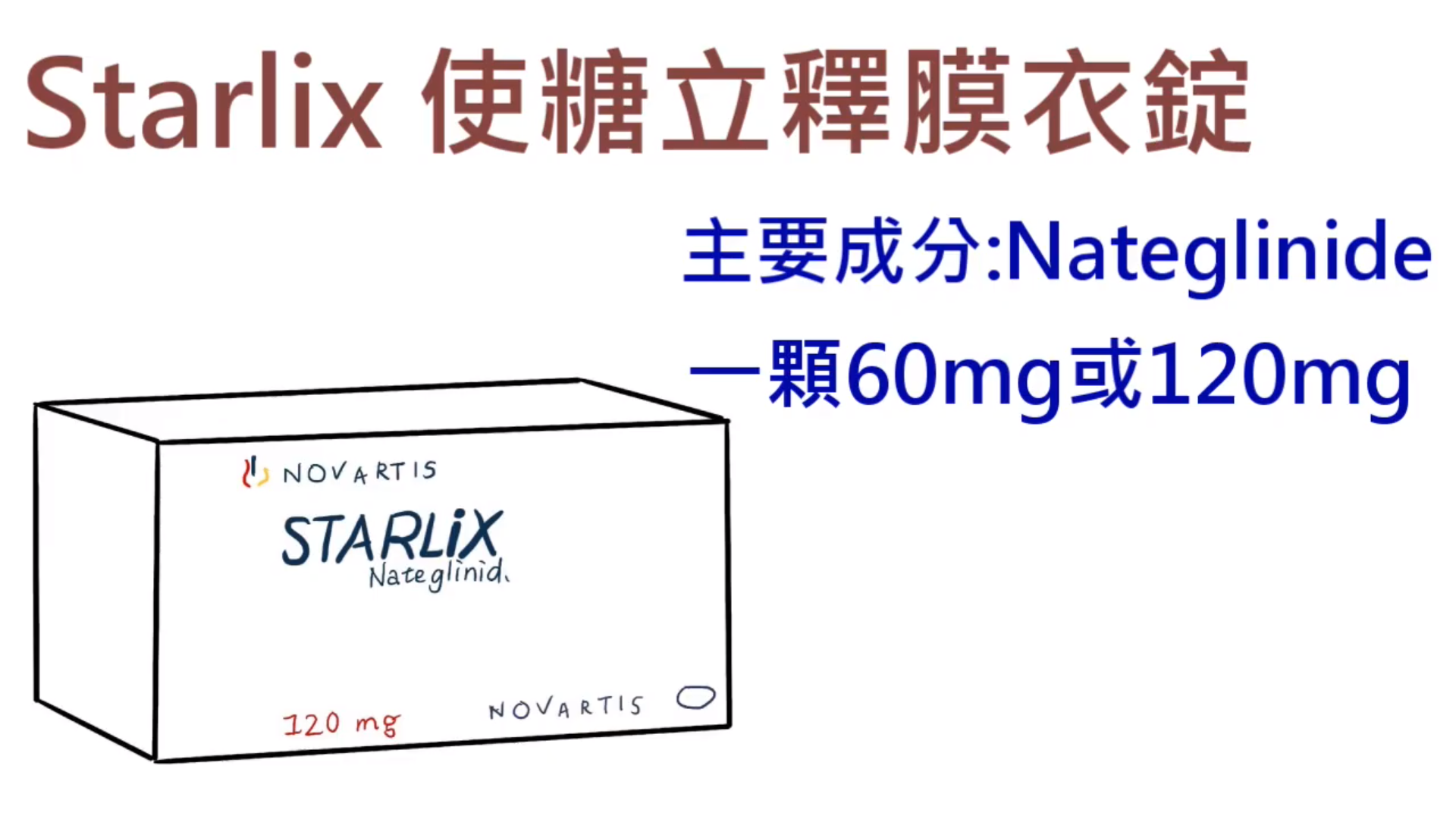 | | NovoNorm (Repaglinide)  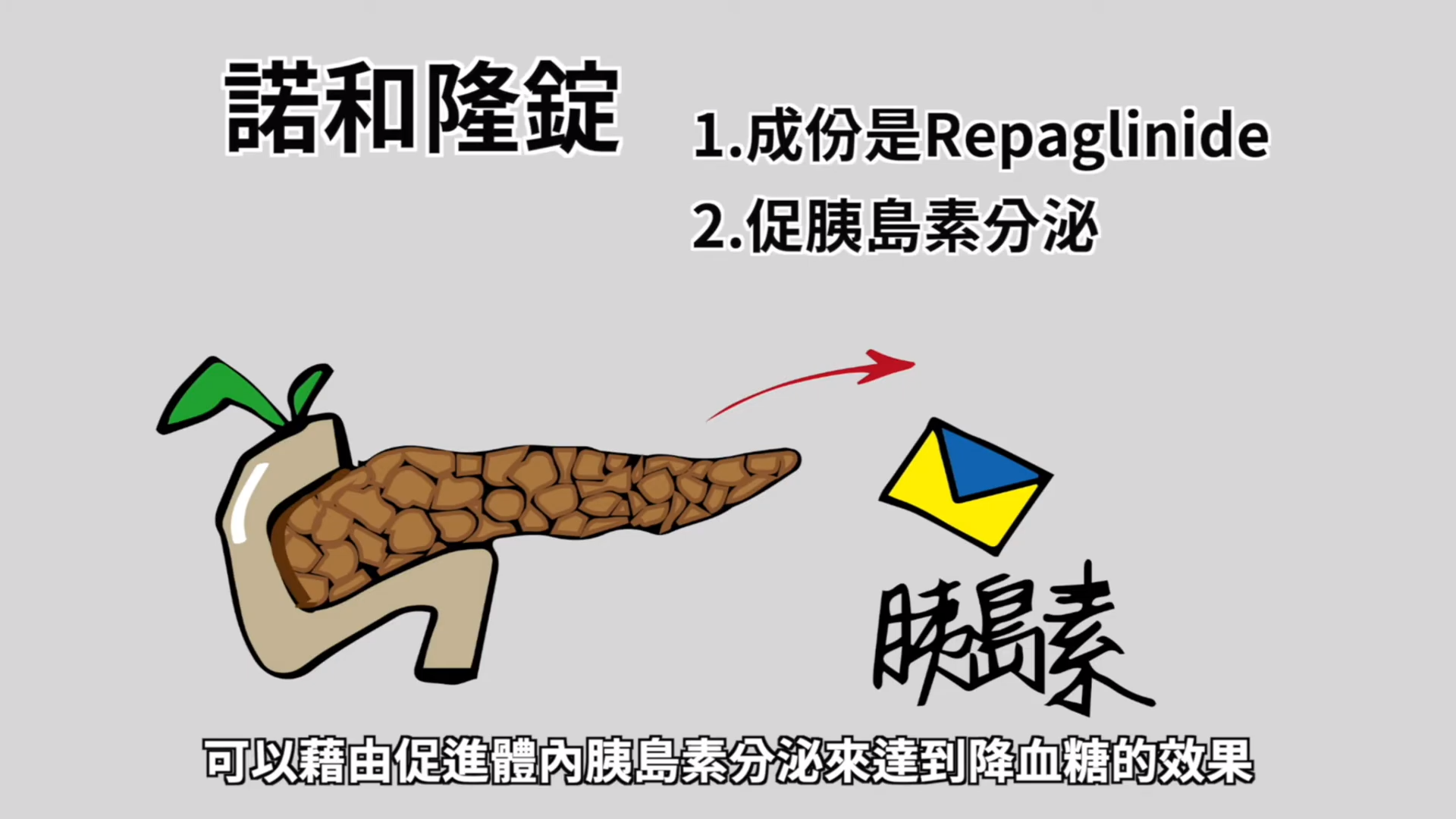 |
| Gliclax (Gliclazide)  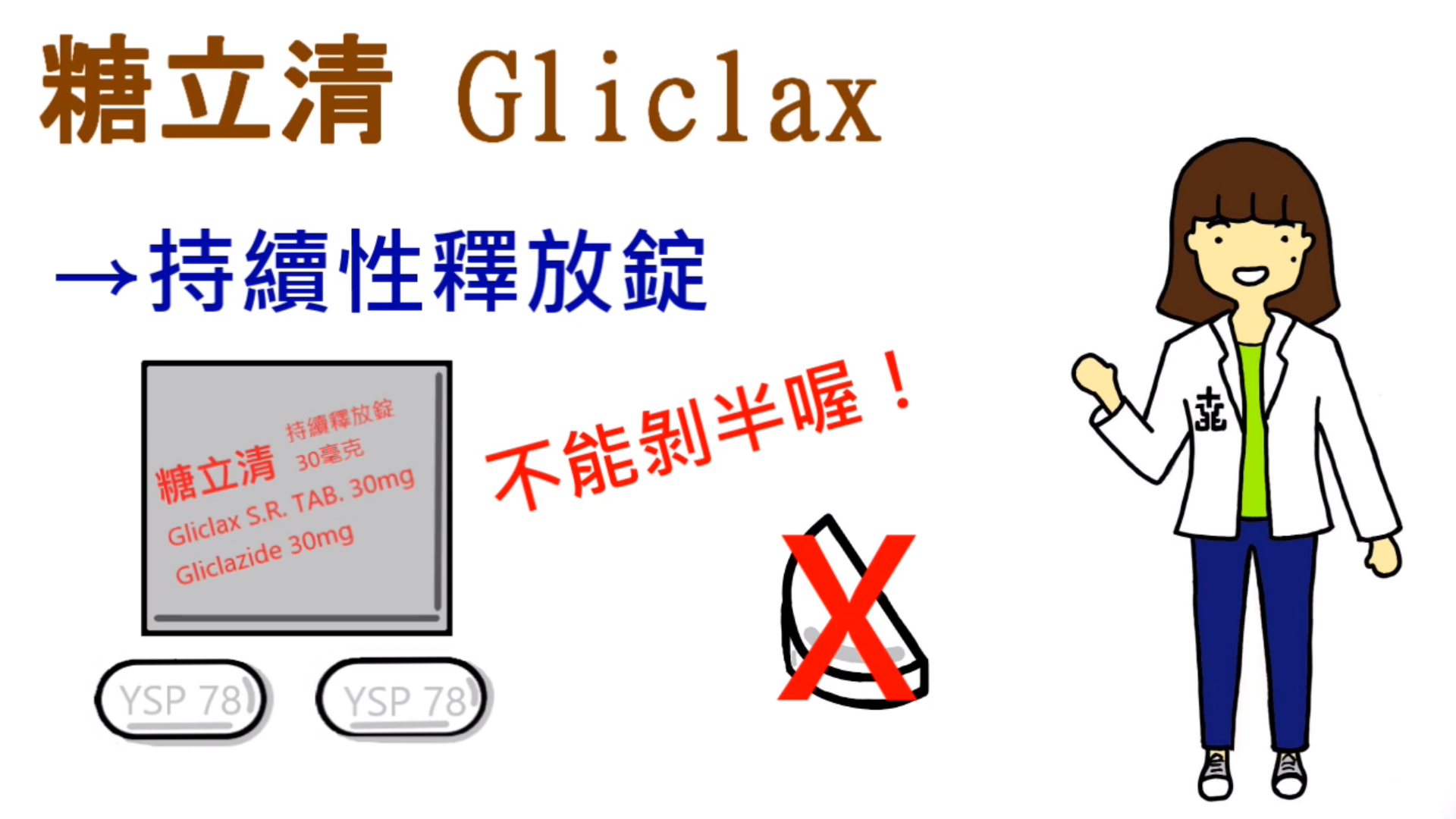 | | Amaryl (Glimepiride) 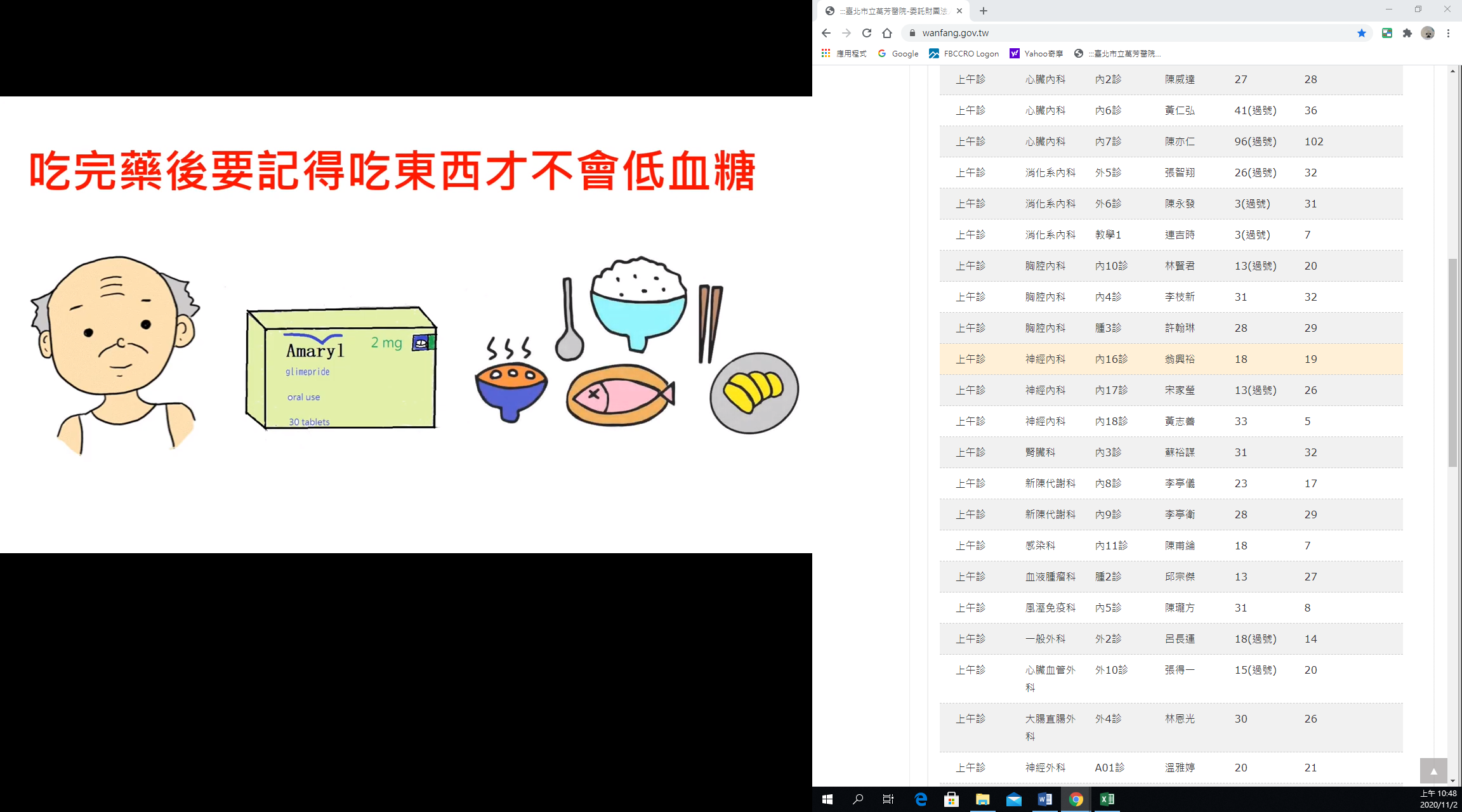 |
| Glipizide (Glipizide)  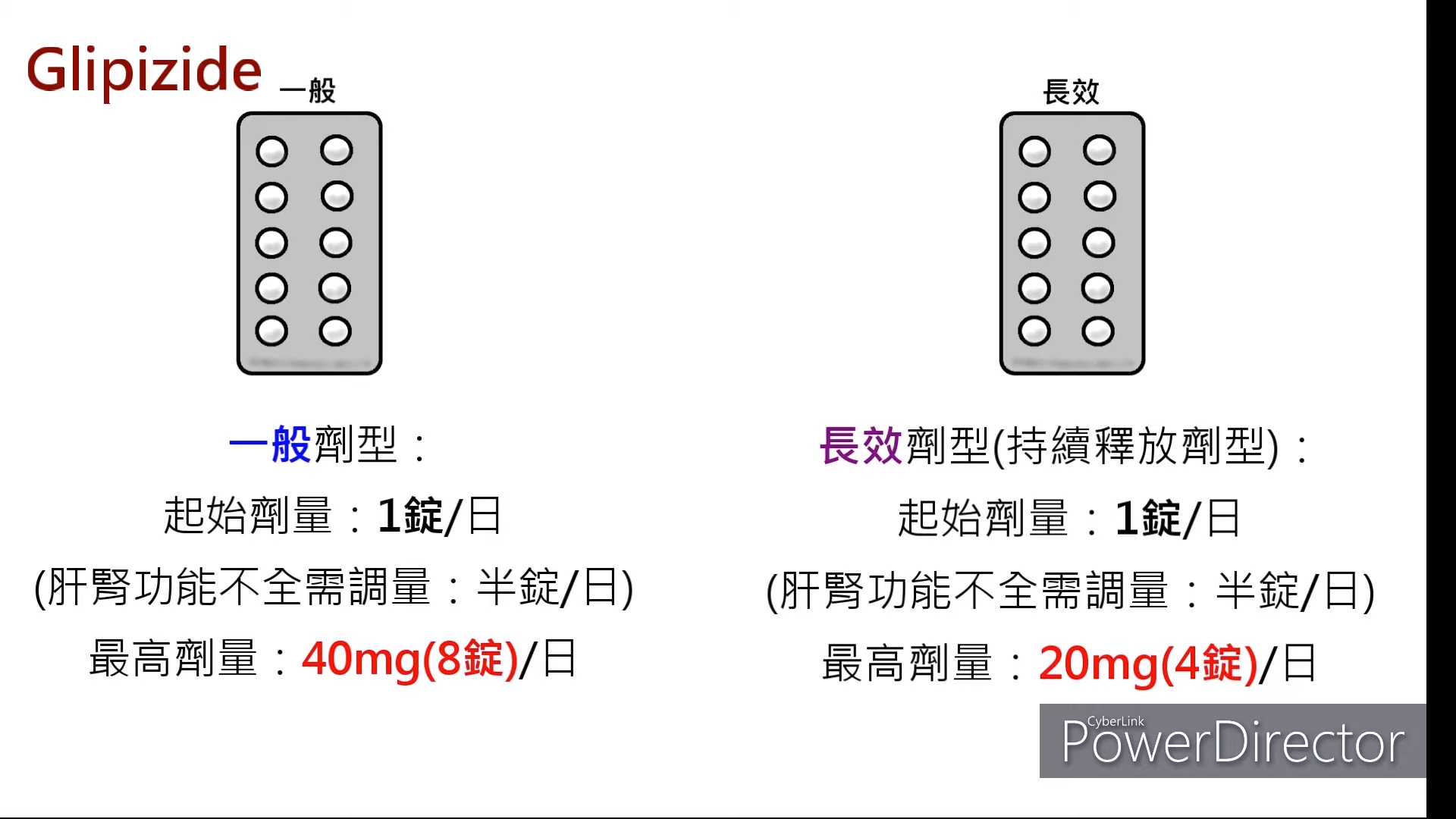 | | Actos (Pioglitazone)  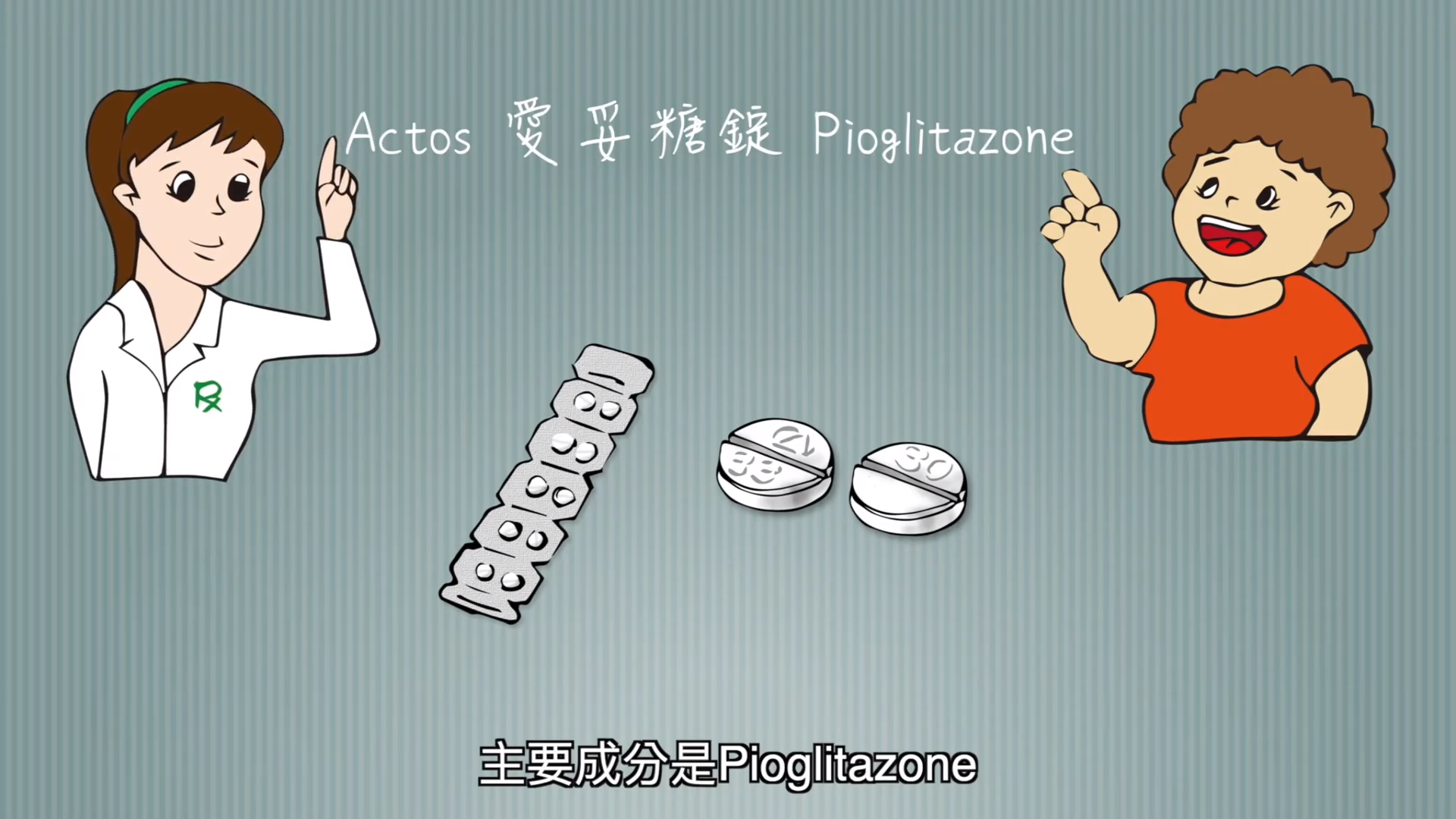 |
| Canaglu (Canagliflozin) 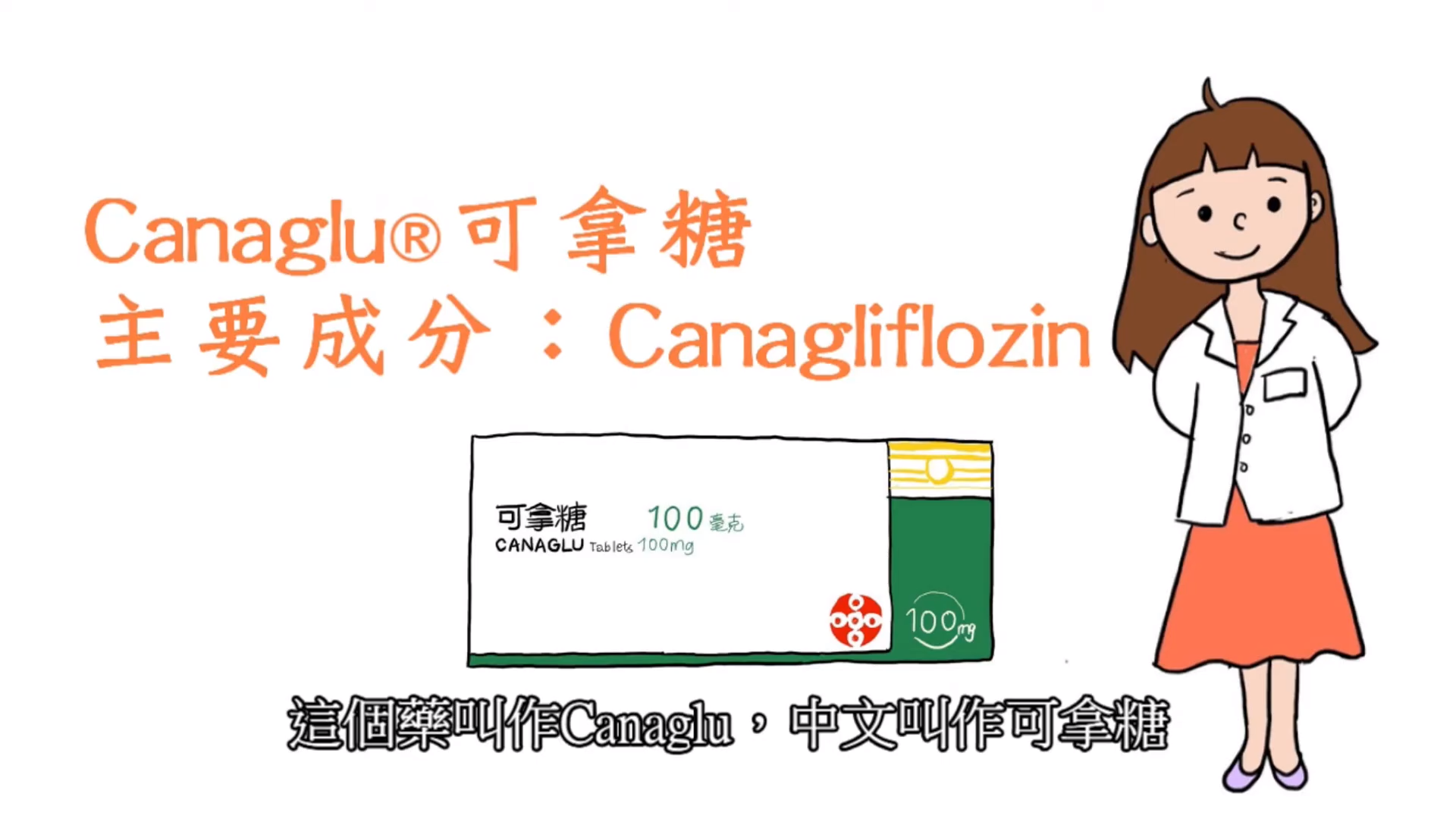 | | Forxiga (Dapagliflozin)  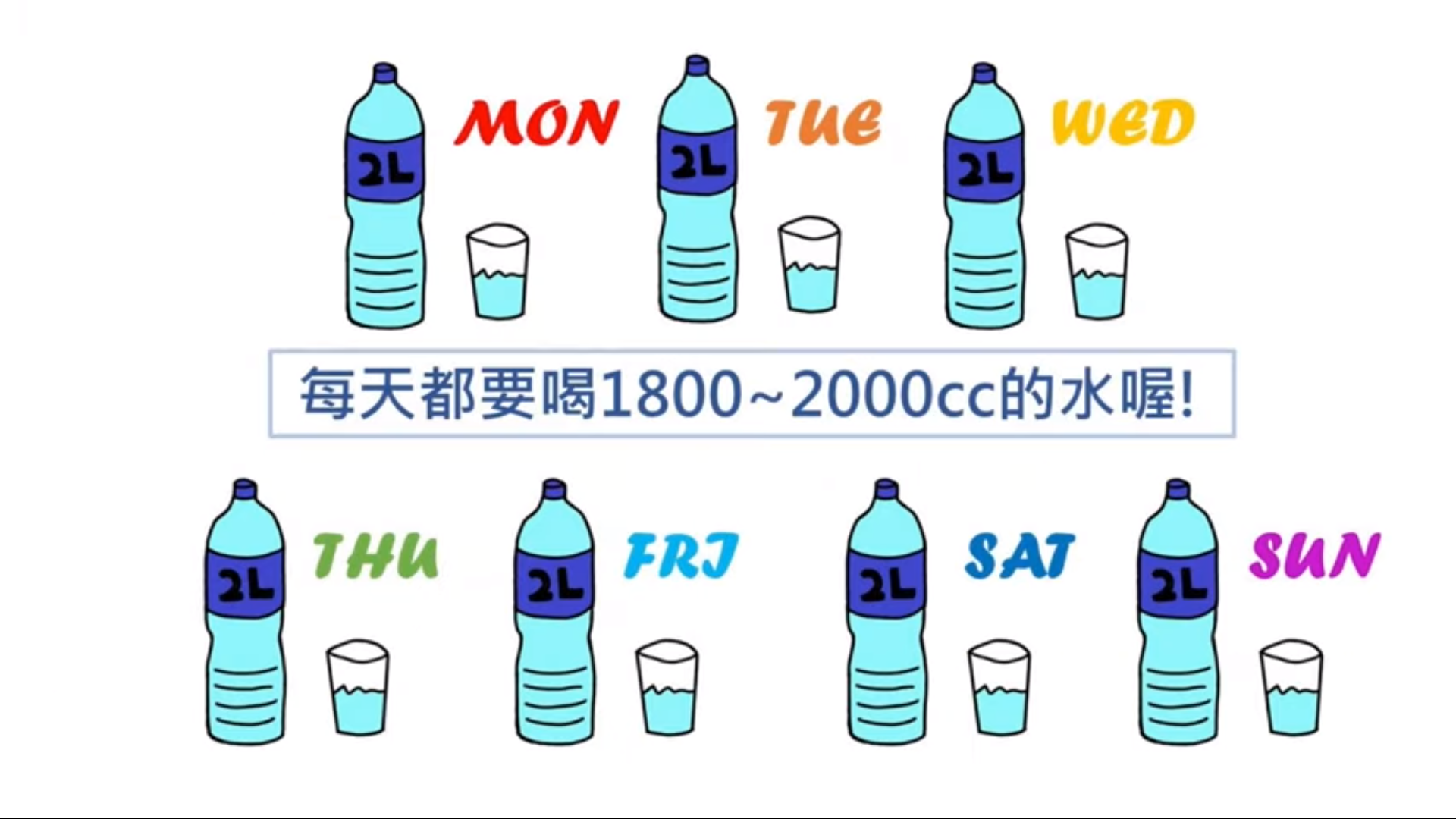 |
| Jardiance (Empagliflozin)  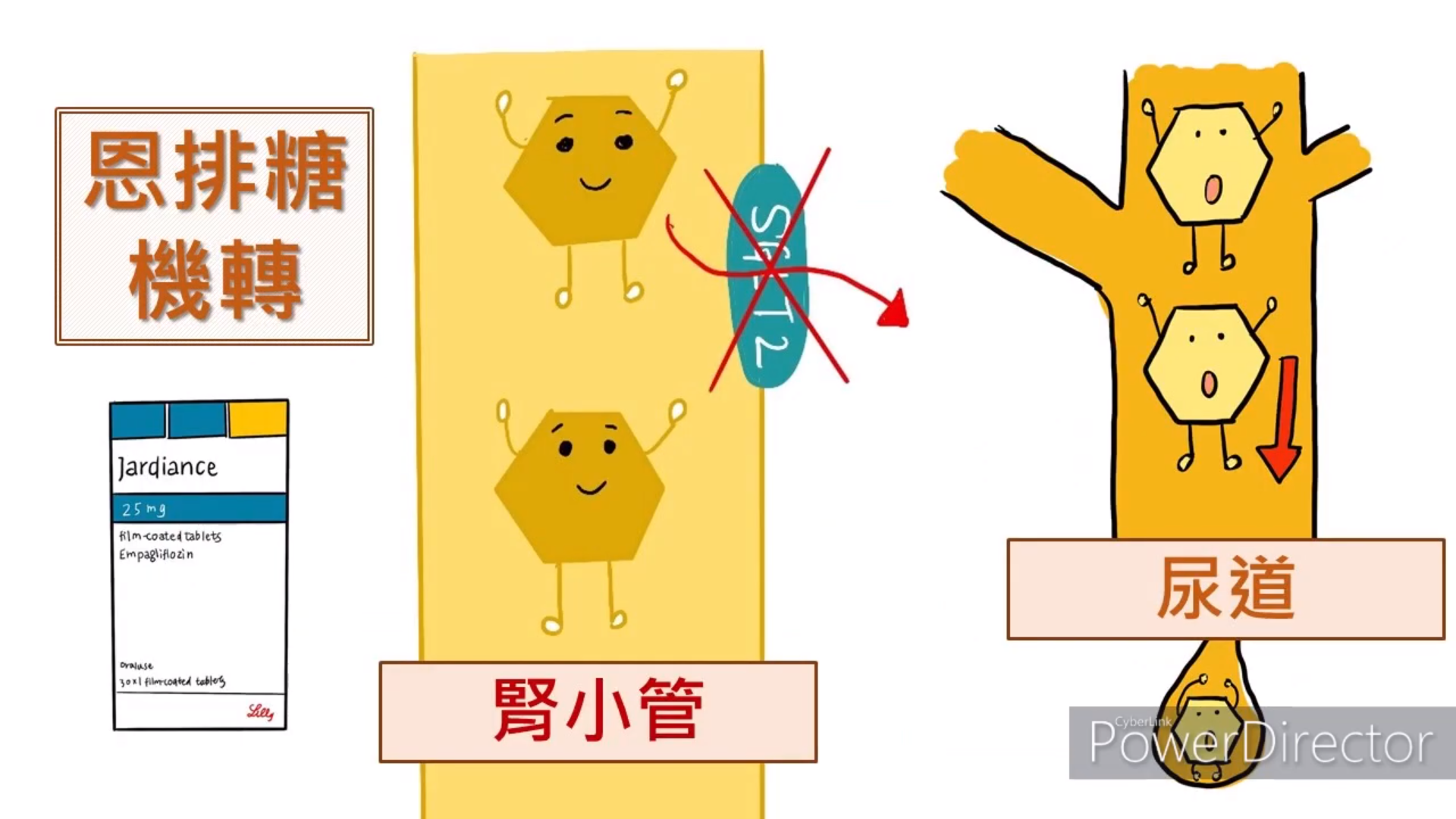 | | Insulin pen injection I – myth  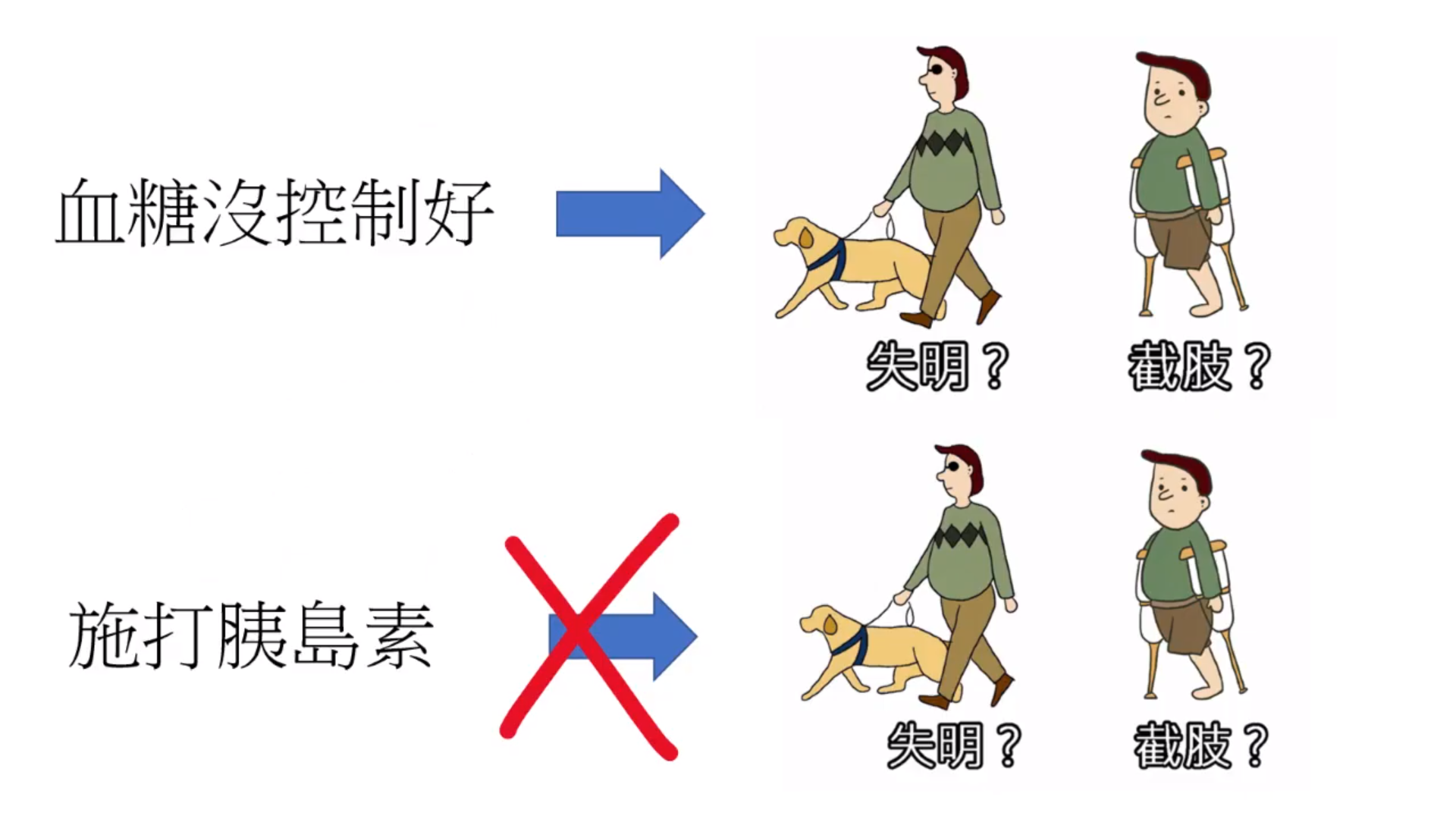 |
| Insulin pen injection II - storage and precautions  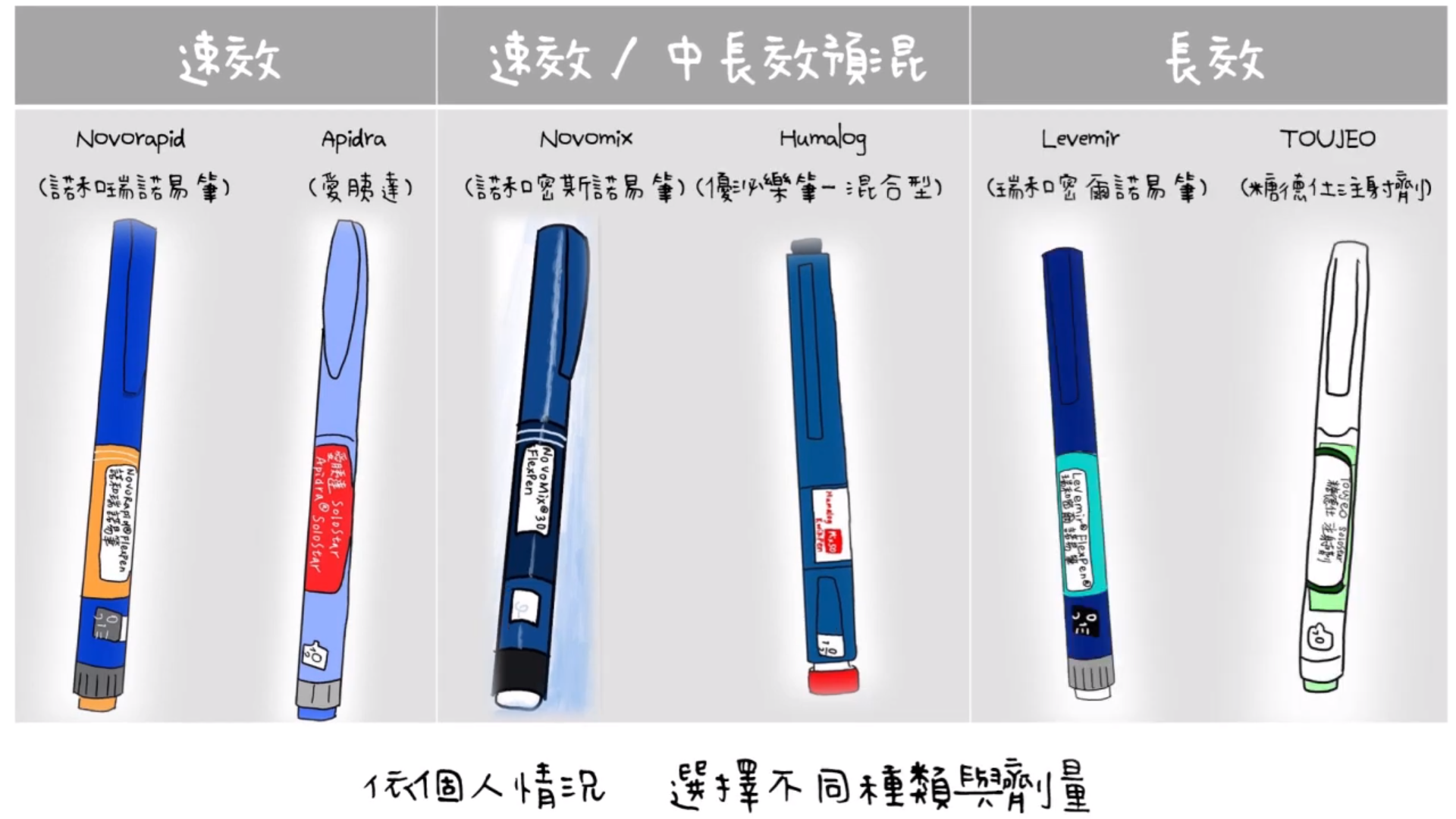 | | I Insulin pen injection III – application method  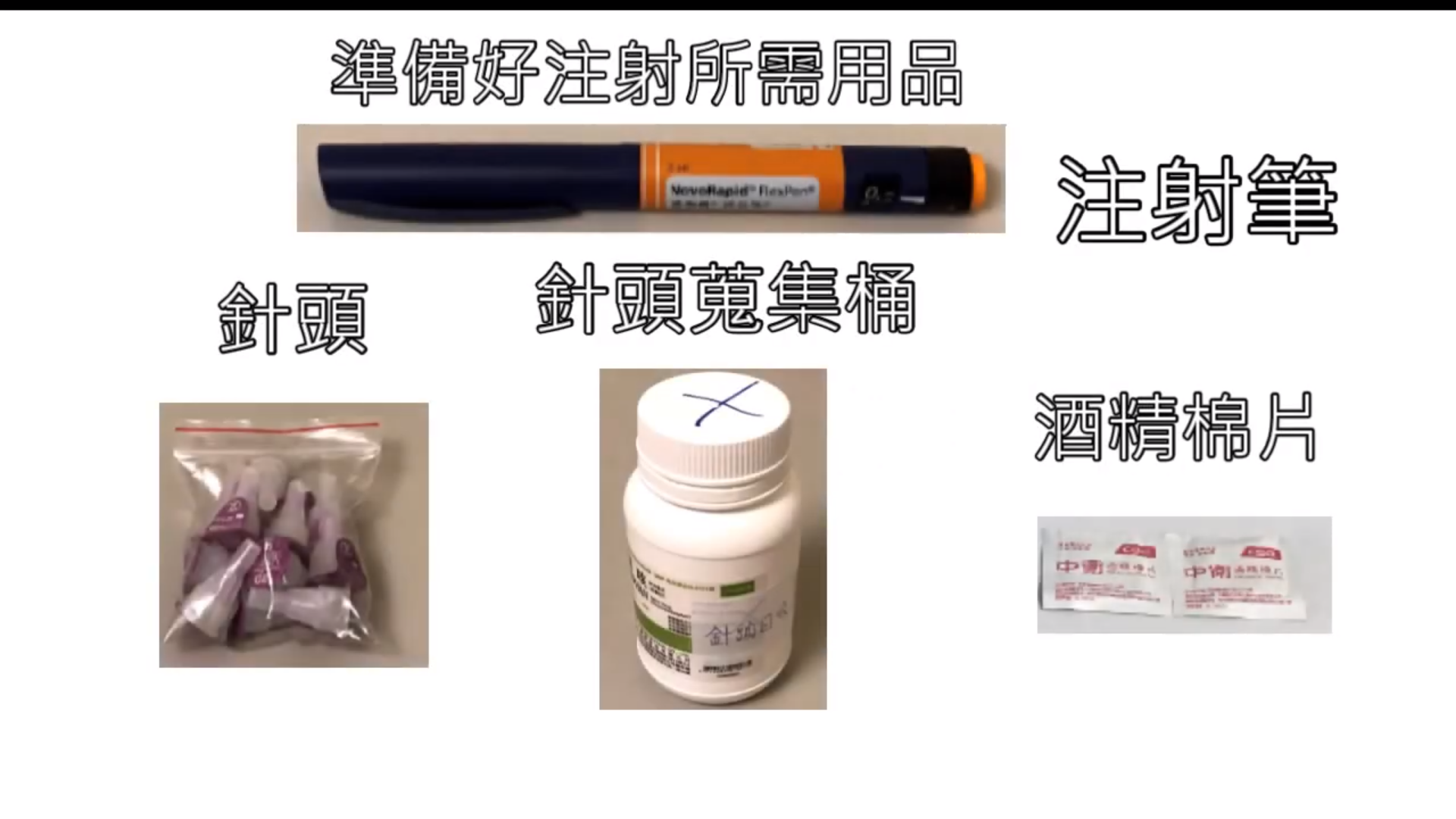 |
| Insulin syringe I – dosage form  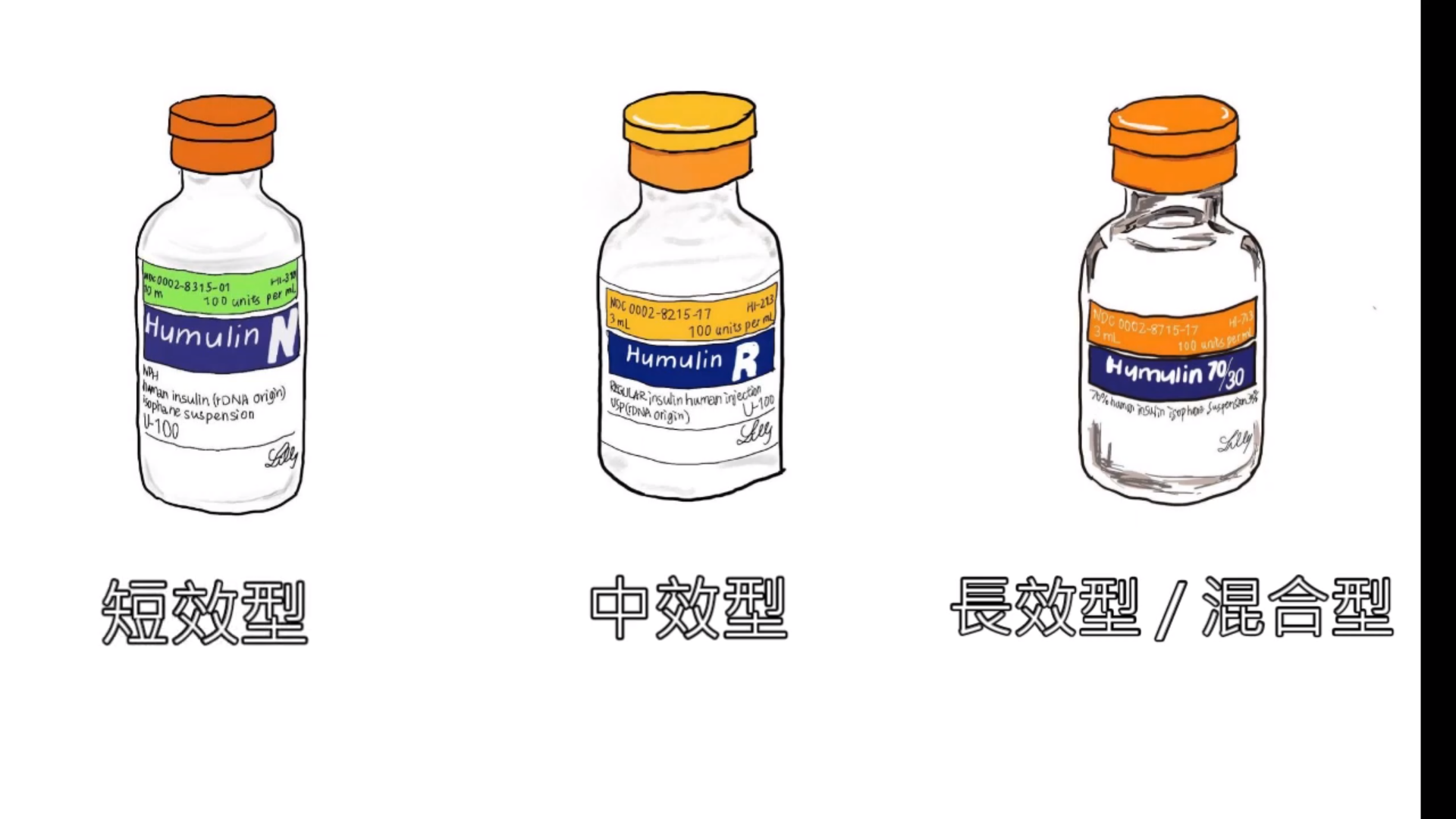 | | Insulin syringe II - Storage and extraction steps  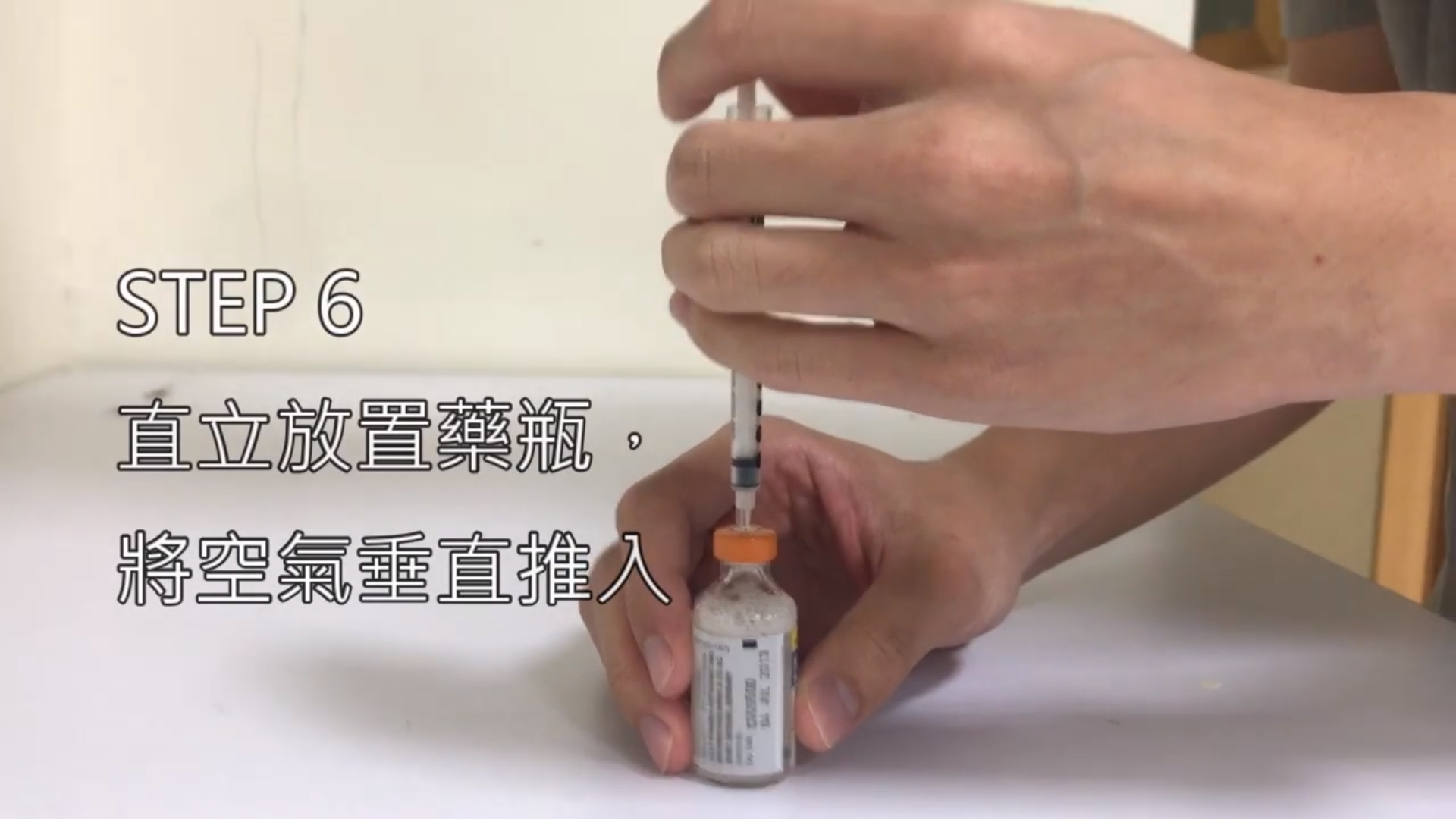 |
| Insulin syringe III - application method  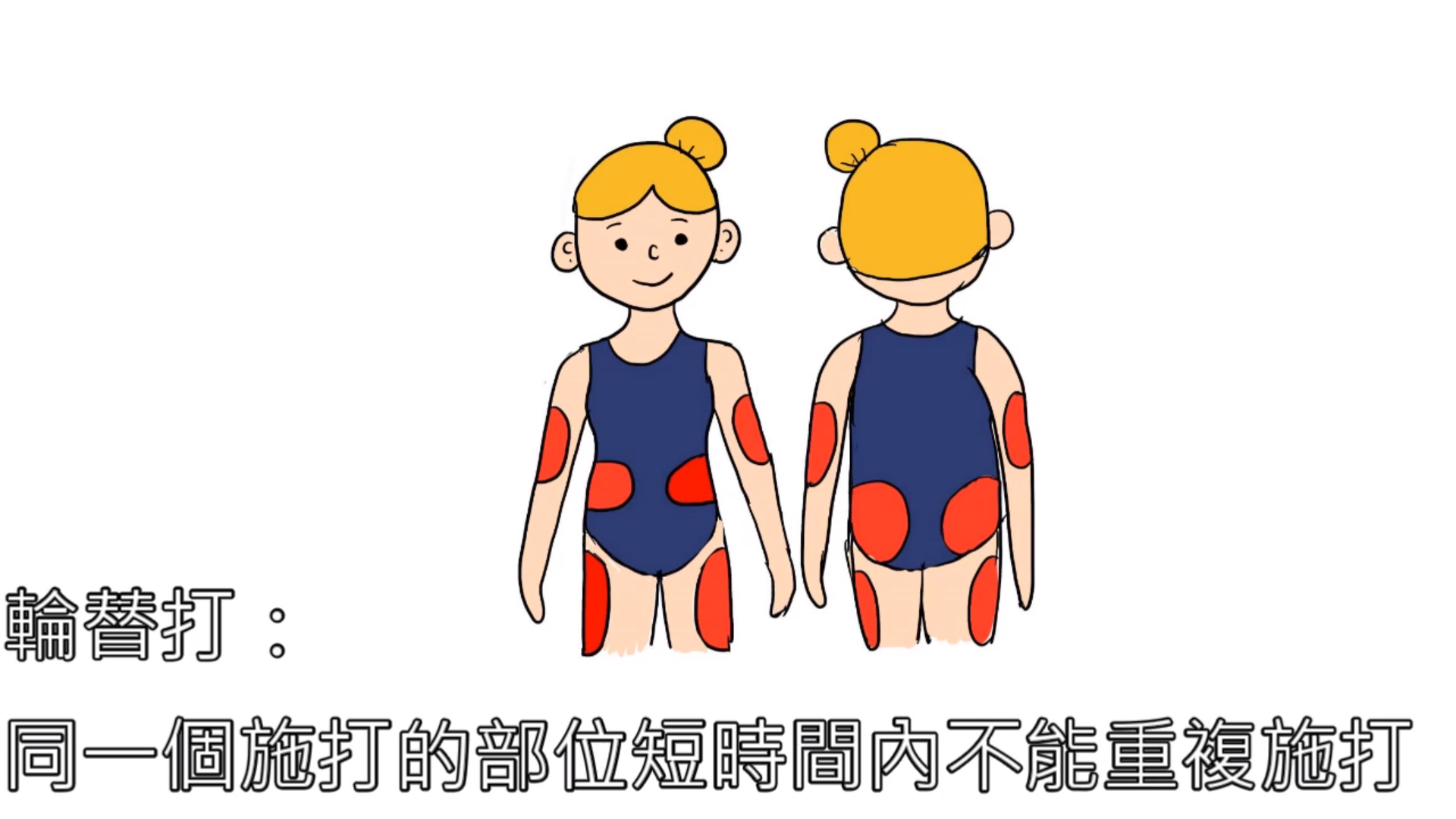 | |  |
| **Quizzes** | | |
| Quiz I  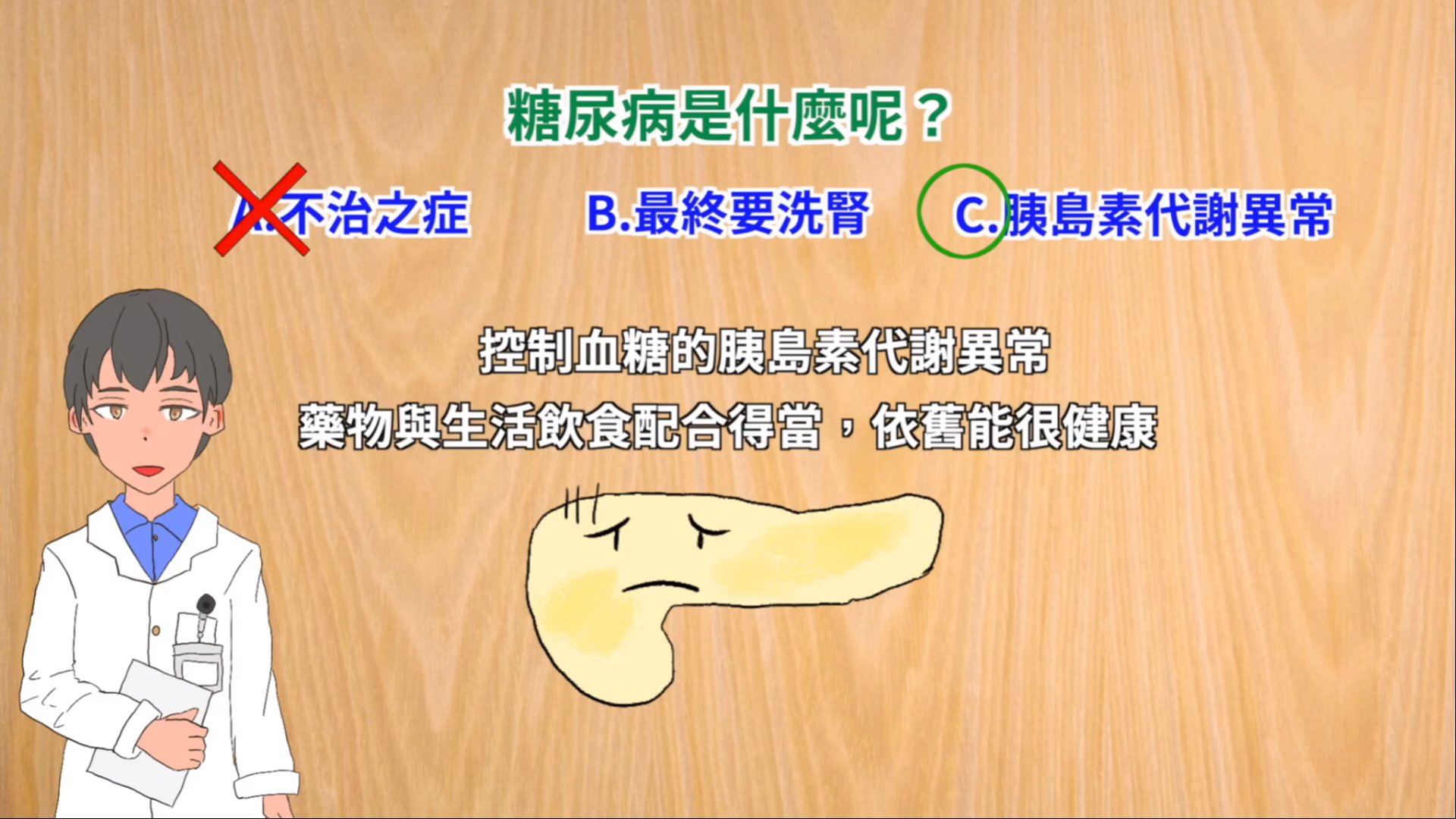   1. (True or False Questions) I can stop medication by myself if the blood glucose level is under controlled. 2. (True or False Questions) If you feel uncomfortable after taking medications, stop taking it and ask doctors or pharmacists for help. 3. (True or False Questions) Exercise is good for diabetes 4. (True or False Questions) Monitoring blood glucose regularly is good for diabetes control. 5. (Multiple choice questions) What is diabetes? A. Incurable disease B. Kidney dialysis eventually C. Abnormal insulin metabolism 6. How to deal with hypoglycemia? | | |
| Quiz II 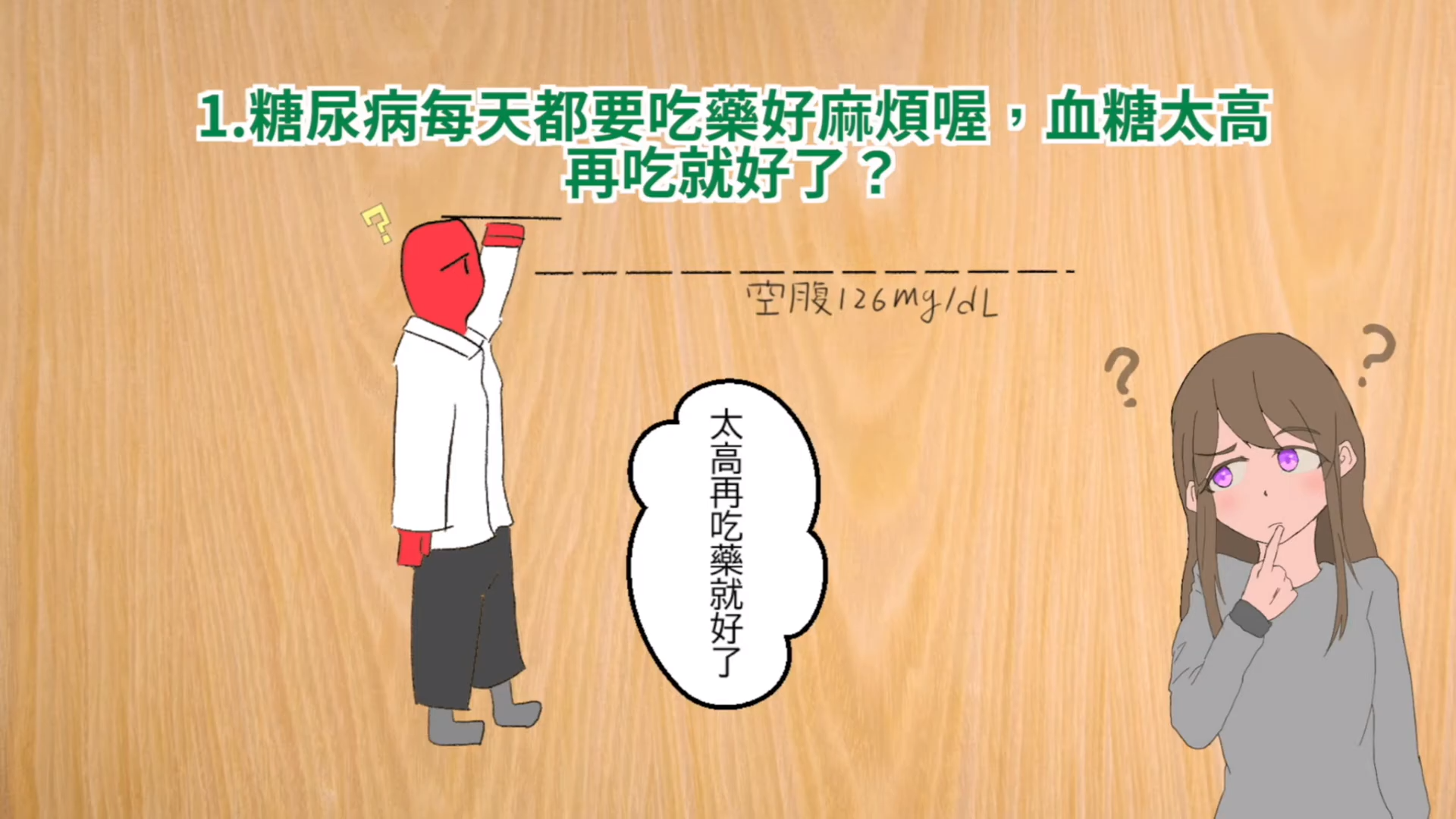   1. (True or False Questions) Medicines needed to be taken only when blood glucose is too high. 2. (True or False Questions) Patient with diabetes is nothing to do with smoking. 3. (True or False Questions) There are many side effects and precautions to the medicine, so the medicine must be bad. 4. What is HbA1C? 5. Which factors may affect blood glucose level? 6. Which one contains more carbohydrate? Beef or sweet potato? 7. Which one contains more lipid? Apple juice or cheese? | | |
| Quiz III 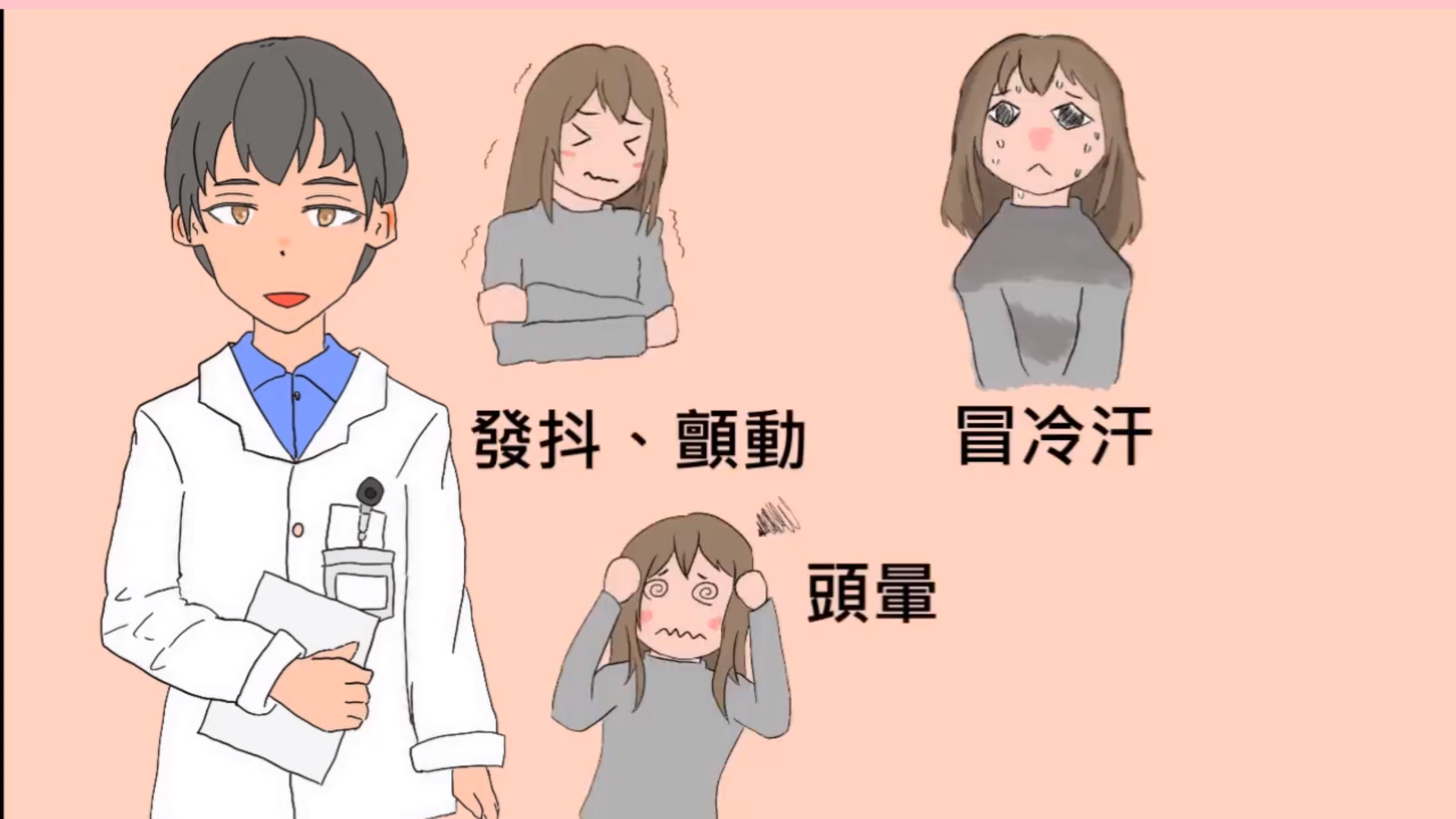   1. (Multiple choice questions) What information is provided on the nutrition label? A. Calories  B. Sodium  C. Carbohydrates  D. Recommended Dietary Allowance E. All of the above 2. If the calorie intake of snacks is limited to 200 calories per day. According to the nutrition label shown. How many biscuit(s) can you eat? 3. (True or False Questions) Those prescribed with insulin treatment are patients with diabetes at the terminal stage. 4. (True or False Questions) To prevent hypoglycemia, patients with diabetes can prepare some candies with them in case of emergency. 5. (Multiple choice questions) What are the possible complications of diabetes? A. Retinopathy  B. Neuropathy C. Nephropathy D. Unhealing wounds E. All of the above 6. (True or False Questions) Dizziness is a sign of hypoglycemia. | | |
| Quiz Ⅳ 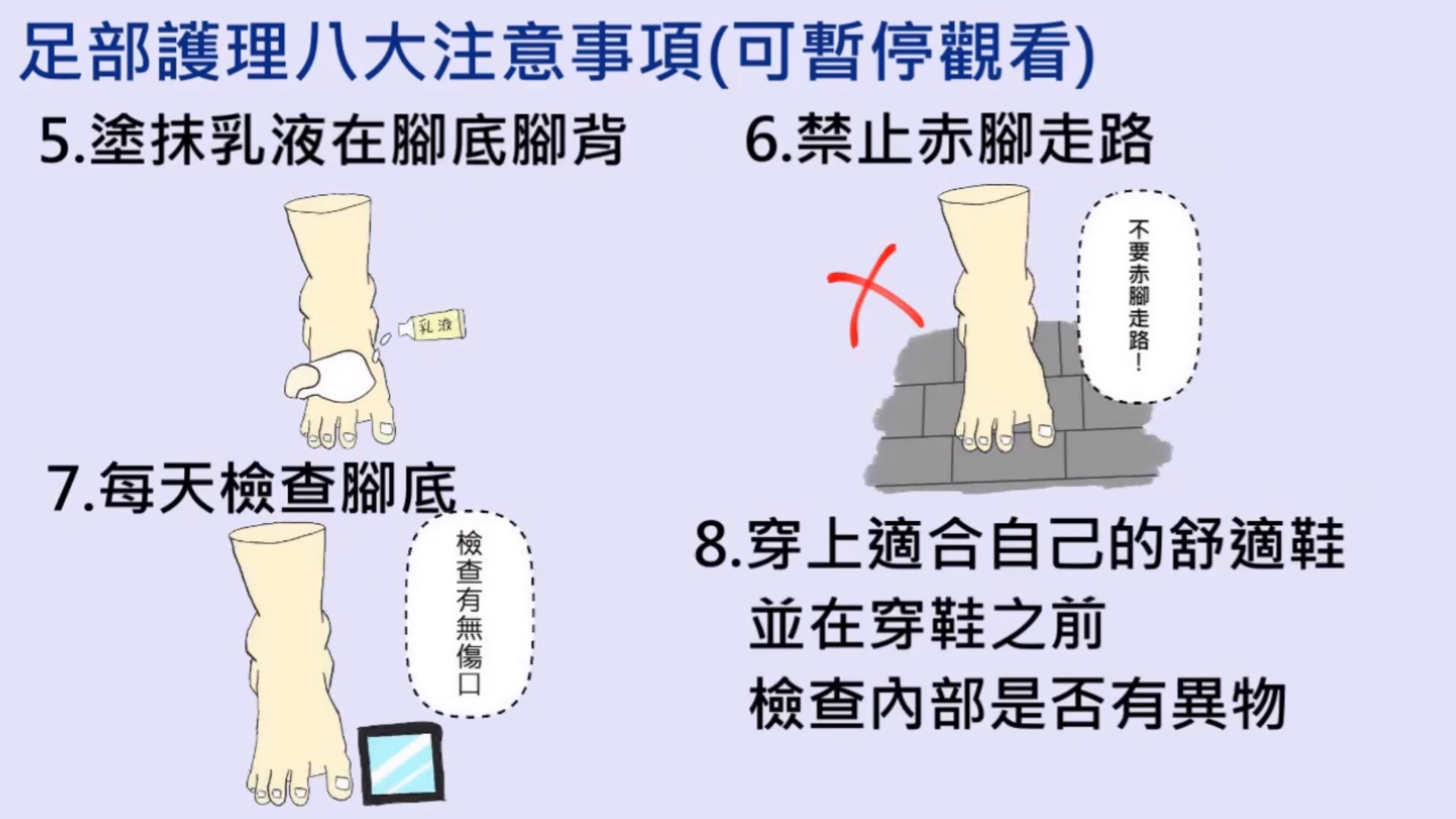   1. What is insulin? 2. (True or False Questions) There are many restrictions on food intake for patients with diabetes, and therefore, they cannot have balanced diet like ordinary people. 3. (True or False Questions) If patient with diabetes is accidentally injured, he/she must take good care of the wound to reduce the risk of infection. 4. (Multiple choice questions) What kind of self-care should patients with diabetes do?  A. Foot care B. Blood glucose monitoring C. Appropriate exercise  D. All of the above 5. (Multiple choice questions) There are many benefits of regular health checkups, including: A. Early detection of diabetes-related complications B. Inspection and adjustments in living habits  C. Prevention of diabetes-related complications  D. All of the above 6. (Multiple choice questions) How can we effectively control diabetes? A. Take the medicine on time  B. Follow the dietary principle  C. Regular blood glucose measurement  D. Regular exercise E. All of the above | | |
