# Supplementary material for: Social Media–Delivered Patient Education to Enhance Self-management and Attitudes of Patients with Type 2 Diabetes During the COVID-19 Pandemic: Randomized Controlled Trial
Source: J Med Internet Res. 2022 Mar 23;24(3):e31449. doi: 10.2196/31449 (PMC8987969; doi:10.2196/31449)
Supplement: Multimedia Appendix 3 [file jmir_v24i3e31449_app3.docx]

Multimedia Appendix 3. Correctness of the Simplified version of the true/false version of the Diabetes Knowledge Scale (SDKS).

| Variable ^a^ | Intervention group (n=91) | | | Control group (n=90) | | |
| --- | --- | --- | --- | --- | --- | --- |
|  | Baseline, mean (SD) | 3 months, mean (SD) | *P* value | Baseline, mean (SD) | 3 months, mean (SD) | *P* value |
| Overall | 68.29 (16.41) | 76.72 (11.65) | <.001 | 64.81 (18.20) | 72.25 (12.61) | <.001 |
| K-1 | 90.11 (30.02) | 92.31 (26.79) | .60 | 85.56 (35.35) | 88.89 (31.60) | .44 |
| K-2 | 47.25 (50.20) | 63.74 (48.30) | .005 | 42.22 (49.67) | 61.11 (49.02) | <.001 |
| K-3 | 63.74 (48.30) | 82.42 (38.28) | .002 | 62.22 (48.75) | 78.89 (41.04) | .005 |
| K-4 | 65.93 (47.66) | 81.32 (39.19) | .01 | 68.89 (46.55) | 73.33 (44.47) | .45 |
| K-5 | 38.46 (48.92) | 63.74 (48.34) | <.001 | 41.11 (49.48) | 46.67 (50.17) | .40 |
| K-6 | 57.14 (49.76) | 87.91 (32.78) | <.001 | 55.56 (49.97) | 70.00 (46.08) | .009 |
| K-7 | 39.56 (49.17) | 46.15 (50.13) | .30 | 38.89 (49.02) | 56.67 (49.83) | .005 |
| K-8 | 79.12 (40.87) | 80.22 (40.05) | .83 | 70.00 (46.08) | 81.11 (39.36) | .05 |
| K-9 | 94.51 (22.91) | 90.11 (30.01) | .16 | 90.00 (30.17) | 93.33 (25.08) | .32 |
| K-10 | 85.71 (35.19) | 93.41 (24.95) | .07 | 83.33 (37.48) | 86.67 (34.18) | .49 |
| K-11 | 64.84 (48.01) | 69.23 (46.41) | .40 | 51.11 (50.27) | 60.00 (49.26) | .16 |
| K-12 | 39.56 (49.17) | 34.07 (47.66) | .32 | 34.44 (47.78) | 40.00 (49.26) | .35 |
| K-13 | 89.01 (31.45) | 90.11 (30.02) | .76 | 80.00 (40.22) | 92.22 (26.93) | .005 |
| K-14 | 85.71 (35.19) | 85.71 (35.19) | 1.0 | 72.22 (45.04) | 75.56 (43.22) | .55 |
| K-15 | 30.77 (46.41) | 49.45 (50.27) | .004 | 26.67 (44.47) | 43.33 (49.83) | .003 |
| K-16 | 98.90 (10.48) | 96.70 (17.95) | .32 | 98.89 (10.54) | 97.78 (14.82) | .56 |
| K-17 | 64.84 (48.01) | 82.42 (38.28) | .002 | 65.56 (47.78) | 80.00 (40.22) | .02 |
| K-18 | 52.75 (50.2) | 65.93 (47.66) | .03 | 57.78 (49.67) | 66.67 (47.40) | .13 |
| K-19 | 65.93 (47.66) | 71.43 (45.43) | .28 | 62.22 (48.75) | 77.78 (41.81) | .01 |
| K-20 | 96.70 (17.95) | 97.80 (14.74) | .66 | 95.56 (20.72) | 95.56 (20.72) | 1.0 |
| K-21 | 80.22 (40.05) | 84.62 (36.28) | .37 | 73.33 (44.47) | 77.78 (41.81) | .41 |
| K-22 | 67.03 (47.27) | 73.63 (44.31) | .20 | 65.56 (47.78) | 63.33 (48.46) | .72 |
| K-23 | 73.91 (44.90) | 91.30 (28.81) | .10 | 83.33 (38.35) | 88.89 (32.34) | .66 |
| K-24 | 78.26 (42.17) | 91.30 (28.81) | .18 | 88.89 (32.34) | 88.89 (32.34) | 1.0 |

^a^ Wilcoxon signed-rank test was performed for percentage of correct SDKS questions.
