# Supplementary material for: Social Media–Delivered Patient Education to Enhance Self-management and Attitudes of Patients with Type 2 Diabetes During the COVID-19 Pandemic: Randomized Controlled Trial
Source: J Med Internet Res. 2022 Mar 23;24(3):e31449. doi: 10.2196/31449 (PMC8987969; doi:10.2196/31449)
Supplement: Multimedia Appendix 4 [file jmir_v24i3e31449_app4.docx]

Multimedia Appendix 4. Scores of the Diabetes Care Profile-Attitudes Toward Diabetes Scales (DCP-ATDS)

| Variable ^a^ | Intervention group (n=91) | | | Control group (n=90) | | |
| --- | --- | --- | --- | --- | --- | --- |
|  | Baseline, mean (SD) | 3 months, mean (SD) | *P* value | Baseline, mean (SD) | 3 months, mean (SD) | *P* value |
| Overall | 3.60 (0.38) | 3.77 (0.49) | .001 | 3.67 (0.40) | 3.65 (0.46) | .58 |
| A-1 | 2.95 (1.02) | 3.21 (1.08) | .02 | 3.07 (1.09) | 3.12 (1.05) | .67 |
| A-2 | 3.20 (1.08) | 3.52 (1.06) | .01 | 3.28 (1.02) | 3.36 (1.03) | .49 |
| A-3 | 3.36 (1.06) | 3.58 (1.00) | .07 | 3.51 (0.90) | 3.56 (0.85) | .63 |
| A-4 | 3.66 (0.85) | 3.86 (0.82) | .04 | 3.58 (0.69) | 3.73 (0.80) | .05 |
| A-5 | 3.60 (1.05) | 3.85 (0.97) | .02 | 3.83 (0.82) | 3.73 (0.85) | .33 |
| A-6 | 3.74 (1.01) | 3.78 (1.01) | .74 | 3.61 (0.91) | 3.80 (0.82) | .10 |
| A-7 | 3.08 (1.08) | 3.16 (1.06) | .51 | 3.40 (0.93) | 3.28 (1.04) | .30 |
| A-8 | 2.93 (1.06) | 3.23 (1.06) | .02 | 3.00 (0.90) | 3.13 (1.0) | .27 |
| A-9 | 3.58 (0.83) | 3.84 (0.81) | .008 | 3.76 (0.61) | 3.64 (0.84) | .21 |
| A-10 | 3.63 (0.90) | 3.80 (0.85) | .11 | 3.74 (0.71) | 3.70 (0.74) | .60 |
| A-11(1) | 3.64 (0.71) | 3.69 (0.73) | .51 | 3.69 (0.65) | 3.60 (0.78) | .34 |
| A-11(2) | 3.69 (0.63) | 3.86 (0.59) | .03 | 3.50 (0.72) | 3.54 (0.75) | .60 |
| A-11(3) | 3.92 (0.65) | 3.80 (0.78) | .21 | 3.79 (0.73) | 3.51 (0.92) | .009 |
| A-11(4) | 3.22 (0.98) | 3.63 (0.96) | .007 | 3.47 (0.75) | 3.44 (0.97) | .87 |
| A-12(1) | 4.31 (0.61) | 4.37 (0.71) | .48 | 4.29 (0.60) | 4.27 (0.68) | .74 |
| A-12(2) | 4.31 (0.61) | 4.46 (0.58) | .04 | 4.21 (0.61) | 4.29 (0.67) | .20 |
| A-12(3) | 4.27 (0.60) | 4.25 (0.71) | .83 | 4.21 (0.61) | 4.02 (0.83) | .03 |
| A-12(4) | 3.46 (1.01) | 3.91 (0.89) | .001 | 3.72 (0.84) | 3.71 (0.85) | .91 |
| A-13 | 3.64 (0.69) | 3.64 (0.68) | 1.0 | 3.81 (0.63) | 3.62 (0.66) | .007 |
| A-14 | 3.66 (0.75) | 3.67 (0.80) | .89 | 3.59 (0.87) | 3.51 (0.81) | .34 |
| A-15 | 3.69 (0.83) | 3.74 (0.71) | .61 | 3.71 (0.77) | 3.68 (0.73) | .68 |
| A-16 | 3.80 (1.18) | 4.14 (1.09) | .008 | 4.08 (0.92) | 3.99 (1.08) | .44 |
| A-17 | 3.41 (1.06) | 3.71 (1.04) | .04 | 3.62 (0.97) | 3.70 (1.0) | .54 |

^a^Paired *t* tests (2-tailed) were performed for DCP-ATDS scores.
