# Supplementary material for: Social Media–Delivered Patient Education to Enhance Self-management and Attitudes of Patients with Type 2 Diabetes During the COVID-19 Pandemic: Randomized Controlled Trial
Source: J Med Internet Res. 2022 Mar 23;24(3):e31449. doi: 10.2196/31449 (PMC8987969; doi:10.2196/31449)
Supplement: Multimedia Appendix 5 [file jmir_v24i3e31449_app5.docx]

Multimedia Appendix 5. Self-care activities

Table S1. Scores of the Summary of Diabetes Self-Care Activities (SDSCA).

| Variable ^a^ | Intervention group (n=91) | | | Control group (n=90) | | |
| --- | --- | --- | --- | --- | --- | --- |
|  | Baseline, mean (SD) | 3 months, mean (SD) | *P* value | Baseline, mean (SD) | 3 months, mean (SD) | *P* value |
| Overall | 3.71 (1.26) | 3.97 (1.18) | .03 | 3.93 (1.40) | 3.86 (1.46) | .52 |
| SCA-1 | 6.81 (0.78) | 6.80 (0.49) | .91 | 6.83 (0.86) | 6.83 (0.45) | 1.0 |
| SCA-2 | 6.35 (1.51) | 6.38 (1.46) | .91 | 6.24 (1.64) | 6.24 (1.58) | 1.0 |
| SCA-3 | 4.15 (2.70) | 4.76 (2.55) | .07 | 4.62 (2.56) | 4.61 (2.94) | .97 |
| SCA-4 | 5.76 (1.74) | 5.37 (1.94) | .11 | 5.62 (2.06) | 5.22 (2.45) | .13 |
| SCA-5 | 3.73 (2.55) | 3.80 (2.66) | .76 | 3.58 (2.64) | 3.48 (2.80) | .68 |
| SCA-6 | 2.99 (2.56) | 3.38 (2.70) | .08 | 3.11 (2.69) | 2.88 (2.79) | .28 |
| SCA-7 | 1.66 (2.43) | 1.73 (2.50) | .72 | 2.09 (2.73) | 2.10 (2.70) | .95 |
| SCA-8 | 1.54 (2.39) | 1.63 (2.42) | .69 | 2.08 (2.75) | 2.07 (2.75) | .96 |
| SCA-9 | 2.87 (3.16) | 4.22 (3.14) | .001 | 3.28 (3.38) | 3.36 (3.25) | .83 |
| SCA-10 | 3.53 (3.34) | 3.76 (3.26) | .52 | 3.80 (3.38) | 3.87 (3.37) | .85 |

^a^Paired *t* tests (2-tailed) were performed for SDSCA scores.

| Table S2. Top five reasons that patients found it difficult to engage in self-care activities |
| --- |
| 1. Patients were too busy at work and might forget to take medicine on time, have meals irregularly, have no time for exercise, or do not cook for themselves. |
| 1. A kitchen and utensils may not be available to patients who live in a rented house. |
| 1. Prices for a general lunch box containing fried chicken or meat were much cheaper than a low-oil, healthy lunch box. Patients on tight financial budget may prefer the cheaper option. |
| 1. Patients who were housewives had to take care of the family and children’s preferences when preparing meals, so it may not be possible for them to follow healthy nutrition principles. |
| 1. It was difficult for patients to control their diet when attending social dinners or gatherings. |
