# Supplementary material for: Social Media–Delivered Patient Education to Enhance Self-management and Attitudes of Patients with Type 2 Diabetes During the COVID-19 Pandemic: Randomized Controlled Trial
Source: J Med Internet Res. 2022 Mar 23;24(3):e31449. doi: 10.2196/31449 (PMC8987969; doi:10.2196/31449)
Supplement: Multimedia Appendix 6 [file jmir_v24i3e31449_app6.docx]

|  | Pre-test, mean (SD) | | | Post-test, mean (SD) | | |
| --- | --- | --- | --- | --- | --- | --- |
| Subscales of  DCP-ATDS^a^ | Without SDSCA improvement (n=87) | With SDSCA improvement (n=94) | *P*-value | Without SDSCA improvement (n=87) | With SDSCA improvement (n=94) | *P*-value |
| Positive attitude | 3.56 (0.68) | 3.49 (0.61) | .47 | 3.63 (0.70) | 3.67 (0.67) | .65 |
| Negative attitude | 3.42 (0.78) | 3.43 (0.68) | .92 | 3.47 (0.72) | 3.60 (0.73) | .22 |
| Self-care ability | 3.68 (0.57) | 3.61 (0.58) | .40 | 3.61 (0.57) | 3.70 (0.54) | .31 |
| Self-care adherence | 3.64 (0.46) | 3.59 (0.47) | .47 | 3.59 (0.54) | 3.68 (0.67) | .36 |
| Importance of care | 4.08 (0.50) | 4.12 (0.55) | .62 | 4.13 (0.61) | 4.19 (0.60) | .53 |

Multimedia Appendix 6. Comparison in Diabetes Care Profile-Attitudes Toward Diabetes Scales (DCP-ATDS) subscales.

^a^*t* tests (2-tailed) were performed for the comparison.
