# Supplementary material for: Social Media–Delivered Patient Education to Enhance Self-management and Attitudes of Patients with Type 2 Diabetes During the COVID-19 Pandemic: Randomized Controlled Trial
Source: J Med Internet Res. 2022 Mar 23;24(3):e31449. doi: 10.2196/31449 (PMC8987969; doi:10.2196/31449)
Supplement: Multimedia Appendix 7 [file jmir_v24i3e31449_app7.pdf]

CONSORT-EHEALTH (V 1.6.1) - Submission/Publication Form

The CONSORT-EHEALTH checklist is intended for authors of randomized trials evaluating web-based and Internet-based applications/interventions, including mobile interventions, electronic games (incl multiplayer games), social media, certain telehealth applications, and other interactive and/or networked electronic applications. Some of the items (e.g. all subitems under item 5 - description of the intervention) may also be applicable for other study designs.

The goal of the CONSORT EHEALTH checklist and guideline is to be
a) a guide for reporting for authors of RCTs,
b) to form a basis for appraisal of an ehealth trial (in terms of validity)

CONSORT-EHEALTH items/subitems are MANDATORY reporting items for studies published in the Journal of Medical Internet Research and other journals / scientific societies endorsing the checklist.

Items numbered 1., 2., 3., 4a., 4b etc are original CONSORT or CONSORT-NPT (non-pharmacologic treatment) items.
Items with Roman numerals (i., ii, iii, iv etc.) are CONSORT-EHEALTH extensions/clarifications.

As the CONSORT-EHEALTH checklist is still considered in a formative stage, we would ask that you also RATE ON A SCALE OF 1-5 how important/useful you feel each item is FOR THE PURPOSE OF THE CHECKLIST and reporting guideline (optional).

Mandatory reporting items are marked with a red *.
In the textboxes, either copy & paste the relevant sections from your manuscript into this form - please include any quotes from your manuscript in QUOTATION MARKS,
or answer directly by providing additional information not in the manuscript, or elaborating on why the item was not relevant for this study.

YOUR ANSWERS WILL BE PUBLISHED AS A SUPPLEMENTARY FILE TO YOUR PUBLICATION IN JMIR AND ARE CONSIDERED PART OF YOUR PUBLICATION (IF ACCEPTED).
Please fill in these questions diligently. Information will not be copyedited, so please use proper spelling and grammar, use correct capitalization, and avoid abbreviations.

DO NOT FORGET TO SAVE AS PDF _AND_ CLICK THE SUBMIT BUTTON SO YOUR ANSWERS ARE IN OUR DATABASE !!!

Citation Suggestion (if you append the pdf as Appendix we suggest to cite this paper in the caption):
Eysenbach G, CONSORT-EHEALTH Group
CONSORT-EHEALTH: Improving and Standardizing Evaluation Reports of Web-based and Mobile Health Interventions
J Med Internet Res 2011;13(4):e126
URL: http://www.jmir.org/2011/4/e126/

doi: 10.2196/jmir.1923
PMID: 22209829

*必填

Your name *

First Last

Cheng Man Leong

Primary Affiliation (short), City, Country *

University of Toronto, Toronto, Canada

Taipei Medical University, Taipei, Taiwan

Your e-mail address *

abc@gmail.com

m301108029@tmu.edu.tw

Title of your manuscript *

Provide the (draft) title of your manuscript.

"Social Media-Delivered Patient Education in Enhancing Type 2 Diabetics Self-Management and Attitudes During the COVID-19 Pandemic: a Randomized Controlled Trial in Taiwan"

Name of your App/Software/Intervention *

If there is a short and a long/alternate name, write the short name first and add the long name in brackets.

TMU-LOVE (Taipei Medical University-Line Orie

Evaluated Version (if any)

e.g. "V1", "Release 2017-03-01", "Version 2.0.27913"

V1, Release 2020-07-14

Language(s) *

What language is the intervention/app in? If multiple languages are available, separate by comma (e.g. "English, French")

Chinese, Mandarin

URL of your Intervention Website or App

e.g. a direct link to the mobile app on app in appstore (itunes, Google Play), or URL of the website. If the intervention is a DVD or hardware, you can also link to an Amazon page.

http://pharmschool.tmu.edu.tw/activity/index.php?type=20

URL of an image/screenshot (optional)

http://pharmschool.tmu.edu.tw/activity/index.php?type=20

Accessibility *

Can an enduser access the intervention presently?

- (●) access is free and open
- ( ) access only for special usergroups, not open
- ( ) access is open to everyone, but requires payment/subscription/in-app purchases
- ( ) app/intervention no longer accessible
- ( ) 其他:

Primary Medical Indication/Disease/Condition *

e.g. "Stress", "Diabetes", or define the target group in brackets after the condition, e.g. "Autism (Parents of children with)", "Alzheimers (Informal Caregivers of)"

Type 2 diabetes

Primary Outcomes measured in trial *

comma-separated list of primary outcomes reported in the trial

glycated hemoglobin (HbA1C)

Secondary/other outcomes

Are there any other outcomes the intervention is expected to affect?

the Simplified True/False version of the Diabetes Knowledge Scale, the Summary of Diabetes Self Care Activities, the Diabetes Care Profile-Attitudes Toward Diabetes Scales, the Newest Vital Sign.

Recommended "Dose" *

What do the instructions for users say on how often the app should be used?

- ○ Approximately Daily
- ◉ Approximately Weekly
- ○ Approximately Monthly
- ○ Approximately Yearly
- ○ "as needed"
- ○ 其他:

Approx. Percentage of Users (starters) still using the app as recommended after 3 months *

- (selected) unknown / not evaluated
- 0-10%
- 11-20%
- 21-30%
- 31-40%
- 41-50%
- 51-60%
- 61-70%
- 71%-80%
- 81-90%
- 91-100%
- 其他:

Overall, was the app/intervention effective? *

- yes: all primary outcomes were significantly better in intervention group vs control
- partly: SOME primary outcomes were significantly better in intervention group vs control
- no statistically significant difference between control and intervention
- potentially harmful: control was significantly better than intervention in one or more outcomes
- inconclusive: more research is needed
- (selected) 其他: Some secondary outcomes were significantly better in intervention gr

Article Preparation Status/Stage *

At which stage in your article preparation are you currently (at the time you fill in this form)

- ○ not submitted yet - in early draft status
- ○ not submitted yet - in late draft status, just before submission
- ○ submitted to a journal but not reviewed yet
- ◉ submitted to a journal and after receiving initial reviewer comments
- ○ submitted to a journal and accepted, but not published yet
- ○ published
- ○ 其他:

Journal *

If you already know where you will submit this paper (or if it is already submitted), please provide the journal name (if it is not JMIR, provide the journal name under "other")

- ○ not submitted yet / unclear where I will submit this
- ◉ Journal of Medical Internet Research (JMIR)
- ○ JMIR mHealth and UHealth
- ○ JMIR Serious Games
- ○ JMIR Mental Health
- ○ JMIR Public Health
- ○ JMIR Formative Research
- ○ Other JMIR sister journal
- ○ 其他:

Is this a full powered effectiveness trial or a pilot/feasibility trial? *

- ( ) Pilot/feasibility
- (●) Fully powered

Manuscript tracking number *

If this is a JMIR submission, please provide the manuscript tracking number under "other" (The ms tracking number can be found in the submission acknowledgement email, or when you login as author in JMIR. If the paper is already published in JMIR, then the ms tracking number is the four-digit number at the end of the DOI, to be found at the bottom of each published article in JMIR)

- ( ) no ms number (yet) / not (yet) submitted to / published in JMIR
- (●) 其他: 31449

TITLE AND ABSTRACT

1a) TITLE: Identification as a randomized trial in the title

1a) Does your paper address CONSORT item 1a? *

I.e does the title contain the phrase "Randomized Controlled Trial"? (if not, explain the reason under "other")

- (●) yes
- ( ) 其他:

1a-i) Identify the mode of delivery in the title

Identify the mode of delivery. Preferably use “web-based” and/or “mobile” and/or “electronic game” in the title. Avoid ambiguous terms like “online”, “virtual”, “interactive”. Use “Internet-based” only if Intervention includes non-web-based Internet components (e.g. email), use “computer-based” or “electronic” only if offline products are used. Use “virtual” only in the context of “virtual reality” (3-D worlds). Use “online” only in the context of “online support groups”. Complement or substitute product names with broader terms for the class of products (such as “mobile” or “smart phone” instead of “iphone”), especially if the application runs on different platforms.

	1	2	3	4	5	
subitem not at all important	○	○	○	○	◉	essential

清除選取的項目

Does your paper address subitem 1a-i? *

Copy and paste relevant sections from manuscript title (include quotes in quotation marks "like this" to indicate direct quotes from your manuscript), or elaborate on this item by providing additional information not in the ms, or briefly explain why the item is not applicable/relevant for your study

"Social Media-Delivered Patient Education in Enhancing Type 2 Diabetics Self-Management and Attitudes During the COVID-19 Pandemic: a Randomized Controlled Trial in Taiwan"

1a-ii) Non-web-based components or important co-interventions in title

Mention non-web-based components or important co-interventions in title, if any (e.g., “with telephone support”).

	1	2	3	4	5	
subitem not at all important	○	○	○	○	◉	essential

清除選取的項目

Does your paper address subitem 1a-ii?

Copy and paste relevant sections from manuscript title (include quotes in quotation marks "like this" to indicate direct quotes from your manuscript), or elaborate on this item by providing additional information not in the ms, or briefly explain why the item is not applicable/relevant for your study

"Social Media-Delivered Patient Education in Enhancing Type 2 Diabetics Self-Management and Attitudes During the COVID-19 Pandemic: a Randomized Controlled Trial in Taiwan"

1a-iii) Primary condition or target group in the title

Mention primary condition or target group in the title, if any (e.g., “for children with Type I Diabetes”) Example: A Web-based and Mobile Intervention with Telephone Support for Children with Type I Diabetes: Randomized Controlled Trial

	1	2	3	4	5	
subitem not at all important	○	○	○	●	○	essential

清除選取的項目

Does your paper address subitem 1a-iii? *

Copy and paste relevant sections from manuscript title (include quotes in quotation marks "like this" to indicate direct quotes from your manuscript), or elaborate on this item by providing additional information not in the ms, or briefly explain why the item is not applicable/relevant for your study

"Social Media-Delivered Patient Education in Enhancing Type 2 Diabetics Self-Management and Attitudes During the COVID-19 Pandemic: a Randomized Controlled Trial in Taiwan"

1b) ABSTRACT: Structured summary of trial design, methods, results, and conclusions

NPT extension: Description of experimental treatment, comparator, care providers, centers, and blinding status.

1b-i) Key features/functionalities/components of the intervention and comparator in the METHODS section of the ABSTRACT

Mention key features/functionalities/components of the intervention and comparator in the abstract. If possible, also mention theories and principles used for designing the site. Keep in mind the needs of systematic reviewers and indexers by including important synonyms. (Note: Only report in the abstract what the main paper is reporting. If this information is missing from the main body of text, consider adding it)

	1	2	3	4	5	
subitem not at all important	○	○	○	○	◉	essential

清除選取的項目

Does your paper address subitem 1b-i? *

Copy and paste relevant sections from the manuscript abstract (include quotes in quotation marks "like this" to indicate direct quotes from your manuscript), or elaborate on this item by providing additional information not in the ms, or briefly explain why the item is not applicable/relevant for your study

"The intervention group received two or three videos every week and care messages every 2 weeks through TMU-LOVE for 3 months, in addition to usual care. The control group only received usual care."

1b-ii) Level of human involvement in the METHODS section of the ABSTRACT

Clarify the level of human involvement in the abstract, e.g., use phrases like “fully automated” vs. “therapist/nurse/care provider/physician-assisted” (mention number and expertise of providers involved, if any). (Note: Only report in the abstract what the main paper is reporting. If this information is missing from the main body of text, consider adding it)

	1	2	3	4	5	
subitem not at all important	○	○	○	○	◉	essential

清除選取的項目

Does your paper address subitem 1b-ii?

Copy and paste relevant sections from the manuscript abstract (include quotes in quotation marks "like this" to indicate direct quotes from your manuscript), or elaborate on this item by providing additional information not in the ms, or briefly explain why the item is not applicable/relevant for your study

"The pharmacists operated TMU-LOVE program had 51 videos, including 10 on understanding diabetes and daily care, six on nutrition care, 21 on diabetes drugs and four on quizzes. The intervention group received two or three videos every week and care messages every 2 weeks through TMU-LOVE for 3 months, in addition to usual care. The control group only received usual care."

1b-iii) Open vs. closed, web-based (self-assessment) vs. face-to-face assessments in the METHODS section of the ABSTRACT

Mention how participants were recruited (online vs. offline), e.g., from an open access website or from a clinic or a closed online user group (closed usergroup trial), and clarify if this was a purely web-based trial, or there were face-to-face components (as part of the intervention or for assessment). Clearly say if outcomes were self-assessed through questionnaires (as common in web-based trials). Note: In traditional offline trials, an open trial (open-label trial) is a type of clinical trial in which both the researchers and participants know which treatment is being administered. To avoid confusion, use “blinded” or “unblinded” to indicated the level of blinding instead of “open”, as “open” in web-based trials usually refers to “open access” (i.e. participants can self-enrol). (Note: Only report in the abstract what the main paper is reporting. If this information is missing from the main body of text, consider adding it)

	1	2	3	4	5	
subitem not at all important	○	○	◉	○	○	essential

清除選取的項目

Does your paper address subitem 1b-iii?

Copy and paste relevant sections from the manuscript abstract (include quotes in quotation marks "like this" to indicate direct quotes from your manuscript), or elaborate on this item by providing additional information not in the ms, or briefly explain why the item is not applicable/relevant for your study

"This open-label randomized clinical trial evaluated the effect of the Taipei Medical University-Line Oriented Video Education (TMU-LOVE) diabetes educational platform delivered through the social media app Line on type 2 diabetes patients’ glycemic control, attitudes, knowledge and self-care activities."
"Patients with type 2 diabetes were recruited from a clinic with physicians’ referral"
"Outcomes were measured at clinical visits through self-reported face-to-face questionnaires at baseline and 3 months after the intervention"

1b-iv) RESULTS section in abstract must contain use data

Report number of participants enrolled/assessed in each group, the use/uptake of the intervention (e.g., attrition/adherence metrics, use over time, number of logins etc.), in addition to primary/secondary outcomes. (Note: Only report in the abstract what the main paper is reporting. If this information is missing from the main body of text, consider adding it)

	1	2	3	4	5	
subitem not at all important	○	○	○	○	◉	essential

清除選取的項目

Does your paper address subitem 1b-iv?

Copy and paste relevant sections from the manuscript abstract (include quotes in quotation marks "like this" to indicate direct quotes from your manuscript), or elaborate on this item by providing additional information not in the ms, or briefly explain why the item is not applicable/relevant for your study

"Overall, 164 patients with type 2 diabetes completed the 3 month study with 82 patients in either of intervention or control group. Change in HbA1C did not significantly differ between groups (6.95 ± 0.87 to 7.03 ± 0.91 in intervention group, P=.34; 6.75 ± 0.63 to 6.76 ± 0.69 in control group, P=.91). The increase in attitudes was significantly higher in the intervention group than control group (0.26, P<.001 vs 0.05, P=0.29). A positive effect on self-care activities was observed in the intervention group, (3.69 ± 1.26 to 3.98 ± 1.23, P=.02). Both groups experienced an increase in knowledge scores at 12 weeks, with the percentage correct changing increase from 67.74% ± 16.99% to 77.32% ± 11.87%, P<.001 and 65.27% ± 18.07% to 72.83% ± 12.93%, P<.001 in the intervention and control group, respectively. Of the 51 videos, medication-related videos had the highest utility rates."

1b-v) CONCLUSIONS/DISCUSSION in abstract for negative trials

Conclusions/Discussions in abstract for negative trials: Discuss the primary outcome - if the trial is negative (primary outcome not changed), and the intervention was not used, discuss whether negative results are attributable to lack of uptake and discuss reasons. (Note: Only report in the abstract what the main paper is reporting. If this information is missing from the main body of text, consider adding it)

	1	2	3	4	5	
subitem not at all important	○	○	◉	○	○	essential

清除選取的項目

Does your paper address subitem 1b-v?

Copy and paste relevant sections from the manuscript abstract (include quotes in quotation marks "like this" to indicate direct quotes from your manuscript), or elaborate on this item by providing additional information not in the ms, or briefly explain why the item is not applicable/relevant for your study

"Overall, 164 patients with type 2 diabetes completed the 3 month study with 82 patients in either of intervention or control group. Change in HbA1C did not significantly differ between groups (6.95 ± 0.87 to 7.03 ± 0.91 in intervention group, P=.34; 6.75 ± 0.63 to 6.76 ± 0.69 in control group, P=.91). The increase in attitudes was significantly higher in the intervention group than control group (0.26, P<.001 vs 0.05, P=0.29). A positive effect on self-care activities was observed in the intervention group, (3.69 ± 1.26 to 3.98 ± 1.23, P=.02). Both groups experienced an increase in knowledge scores at 12 weeks, with the percentage correct changing increase from 67.74% ± 16.99% to 77.32% ± 11.87%, P<.001 and 65.27% ± 18.07% to 72.83% ± 12.93%, P<.001 in the intervention and control group, respectively. Of the 51 videos, medication-related videos had the highest utility rates."

INTRODUCTION

2a) In INTRODUCTION: Scientific background and explanation of rationale

2a-i) Problem and the type of system/solution

Describe the problem and the type of system/solution that is object of the study: intended as stand-alone intervention vs. incorporated in broader health care program? Intended for a particular patient population? Goals of the intervention, e.g., being more cost-effective to other interventions, replace or complement other solutions? (Note: Details about the intervention are provided in “Methods” under 5)

	1	2	3	4	5	
subitem not at all important	○	○	○	○	◉	essential

清除選取的項目

Does your paper address subitem 2a-i? *

Copy and paste relevant sections from the manuscript (include quotes in quotation marks "like this" to indicate direct quotes from your manuscript), or elaborate on this item by providing additional information not in the ms, or briefly explain why the item is not applicable/relevant for your study

"The development of effective diabetes education programs for patients' self-management has been a challenge for healthcare professionals. The sudden outbreak of the COVID-19 pandemic created further difficulty by limiting face-to-face diabetes education. The complicated pathology of diabetes not only requires pharmacotherapy, but also effective patient engagement with daily self-care. Previous studies demonstrated that patients with better disease-related knowledge, attitudes, practice, and self-efficacy were associated with better glycemic control [1]. However, it is extremely difficult for patients with diabetes to maintain a healthy life-style and effective self-management. The excessive workload in healthcare settings during the COVID-19 pandemics as well as the burden in patient to healthcare professional ratio restricts the time for sufficient patient education. The worldwide increasing prevalence rates of diabetes reflect the unmet need for health education which call for innovative and effective educational programs."
"The contents of diabetic education are very complicated as demonstrated by Diabetes Self-Management Education and Support (DSMES) and the American Association of Diabetes Educators seven self-care behaviors (AADE7™). To cover all aspects required for day-to-day living with diabetes, education is recommended to include healthy eating, regu¬lar physical activities, self-monitoring of blood glucose (SMBG), com¬pliance with medications, problem-solving skills, healthy coping skills, and other risk-reducing behav¬iors [2]. These aspects were theoretically proven to effectively enhance health outcomes [3-7], however, educating patients on all these topics might not be efficient in busy clinical settings. A previous study reported the time needed to teach diabetes self-care was around 4 hours per patient, which is difficult to achieve in clinical practice [8]. COVID-19 further limited healthcare providers' ability to complete the elements of diabetes patient education recommended by international practice guidelines. Remote learning utilizing advanced technologies may potentially provide a key solution to address the pressures created by this burden of face-to-face education."

2a-ii) Scientific background, rationale: What is known about the (type of) system

Scientific background, rationale: What is known about the (type of) system that is the object of the study (be sure to discuss the use of similar systems for other conditions/diagnoses, if appropiate), motivation for the study, i.e. what are the reasons for and what is the context for this specific study, from which stakeholder viewpoint is the study performed, potential impact of findings [2]. Briefly justify the choice of the comparator.

	1	2	3	4	5	
subitem not at all important	○	○	○	○	◉	essential

清除選取的項目

Does your paper address subitem 2a-ii? *

Copy and paste relevant sections from the manuscript (include quotes in quotation marks "like this" to indicate direct quotes from your manuscript), or elaborate on this item by providing additional information not in the ms, or briefly explain why the item is not applicable/relevant for your study

"Mobile health (mHealth) technology is increasingly being integrated into health care to meet the demands of diabetic care during the COVID-19 pandemic. It was proven to effectively in enhance health outcomes, such as medication adherence, glycated hemoglobin (HbA1C), and self-managements [9-13]. Research has shown that videos, a method of multimedia education, are more effective than the written information in terms of engagement and information uptake [14, 15]. Videos can deliver information through visual and audio elements and require less cognitive effort for information processing [14, 15]. However, no previous study has attempted to develop a video diabetes education program based on social media with a two-way communication component."

2b) In INTRODUCTION: Specific objectives or hypotheses

Does your paper address CONSORT subitem 2b? *

Copy and paste relevant sections from the manuscript (include quotes in quotation marks "like this" to indicate direct quotes from your manuscript), or elaborate on this item by providing additional information not in the ms, or briefly explain why the item is not applicable/relevant for your study

"This study developed an educational program, Taipei Medical University-Line Oriented Video Education (TMU-LOVE), based on the AADE7™. The social media platform employed in this study, LINE (©LINE Corporation, Tokyo, Japan), is one of the most popular and user-friendly social media platforms in Taiwan, with high acceptance rate of up to 91.5% among the population over 50 years-of-age [16]. The aims of this randomized controlled trial conducted during the COVID-19 pandemic were: (1) to evaluate the effectiveness of TMU-LOVE on the changes in patients' HbA1C, knowledge, attitudes and self-care activities before and after the intervention; (2) to study the impacts of TMU-LOVE on changes in knowledge of diabetes patients with high and low levels of health literacy; and (3) to explore the associations between diabetes patients' attitudes and self-care activities."

METHODS

3a) Description of trial design (such as parallel, factorial) including allocation ratio

Does your paper address CONSORT subitem 3a? *

Copy and paste relevant sections from the manuscript (include quotes in quotation marks "like this" to indicate direct quotes from your manuscript), or elaborate on this item by providing additional information not in the ms, or briefly explain why the item is not applicable/relevant for your study

"The pharmacists allocated participants in a 1:1 ratio to either the control or intervention group according to a random sequence, which was generated by a digital program before launching the study."

3b) Important changes to methods after trial commencement (such as eligibility criteria), with reasons

Does your paper address CONSORT subitem 3b? *

Copy and paste relevant sections from the manuscript (include quotes in quotation marks "like this" to indicate direct quotes from your manuscript), or elaborate on this item by providing additional information not in the ms, or briefly explain why the item is not applicable/relevant for your study

No, there was no change to methods after trial commencement.

3b-i) Bug fixes, Downtimes, Content Changes

Bug fixes, Downtimes, Content Changes: ehealth systems are often dynamic systems. A description of changes to methods therefore also includes important changes made on the intervention or comparator during the trial (e.g., major bug fixes or changes in the functionality or content) (5-iii) and other “unexpected events” that may have influenced study design such as staff changes, system failures/downtimes, etc. [2].

	1	2	3	4	5	
subitem not at all important	○	○	○	◉	○	essential

清除選取的項目

Does your paper address subitem 3b-i?

Copy and paste relevant sections from the manuscript (include quotes in quotation marks "like this" to indicate direct quotes from your manuscript), or elaborate on this item by providing additional information not in the ms, or briefly explain why the item is not applicable/relevant for your study

There was no bug fixes, downtimes, content changes in the intervention during the trial.

4a) Eligibility criteria for participants

Does your paper address CONSORT subitem 4a? *

Copy and paste relevant sections from the manuscript (include quotes in quotation marks "like this" to indicate direct quotes from your manuscript), or elaborate on this item by providing additional information not in the ms, or briefly explain why the item is not applicable/relevant for your study

"Eligible participants were asked to complete a written consent form before joining the study. All diabetes patients aged 20 years or older, who had at least one HbA1C datum point ≥ 6% in the past 6 months and possessed a smart cellphone with the Line app were invited to join the study. Patients with gestational diabetes, a cognitive impairment, or on dietary control alone were excluded."

4a-i) Computer / Internet literacy

Computer / Internet literacy is often an implicit “de facto” eligibility criterion - this should be explicitly clarified.

	1	2	3	4	5	
subitem not at all important	○	○	●	○	○	essential

清除選取的項目

Does your paper address subitem 4a-i?

Copy and paste relevant sections from the manuscript (include quotes in quotation marks "like this" to indicate direct quotes from your manuscript), or elaborate on this item by providing additional information not in the ms, or briefly explain why the item is not applicable/relevant for your study

"All diabetes patients aged 20 years or older, who had at least one HbA1C datum point ≥ 6% in the past 6 months and possessed a smart cellphone with the Line app were invited to join the study."

4a-ii) Open vs. closed, web-based vs. face-to-face assessments:

Open vs. closed, web-based vs. face-to-face assessments: Mention how participants were recruited (online vs. offline), e.g., from an open access website or from a clinic, and clarify if this was a purely web-based trial, or there were face-to-face components (as part of the intervention or for assessment), i.e., to what degree got the study team to know the participant. In online-only trials, clarify if participants were quasi-anonymous and whether having multiple identities was possible or whether technical or logistical measures (e.g., cookies, email confirmation, phone calls) were used to detect/prevent these.

	1	2	3	4	5	
subitem not at all important	○	○	○	○	◉	essential

清除選取的項目

Does your paper address subitem 4a-ii? *

Copy and paste relevant sections from the manuscript (include quotes in quotation marks "like this" to indicate direct quotes from your manuscript), or elaborate on this item by providing additional information not in the ms, or briefly explain why the item is not applicable/relevant for your study

"This open-label randomized controlled trial (RCT) conducted between July, 2020 and January, 2021."
"Participants were recruited at the Endocrinology and Metabolism Clinic of Wan-Fang Hospital (Taipei, Taiwan) by physicians."

4a-iii) Information giving during recruitment

Information given during recruitment. Specify how participants were briefed for recruitment and in the informed consent procedures (e.g., publish the informed consent documentation as appendix, see also item X26), as this information may have an effect on user self-selection, user expectation and may also bias results.

	1	2	3	4	5	
subitem not at all important	○	○	◉	○	○	essential

清除選取的項目

Does your paper address subitem 4a-iii?

Copy and paste relevant sections from the manuscript (include quotes in quotation marks "like this" to indicate direct quotes from your manuscript), or elaborate on this item by providing additional information not in the ms, or briefly explain why the item is not applicable/relevant for your study

"Eligible participants were asked to complete a written consent form before joining the study."

4b) Settings and locations where the data were collected

Does your paper address CONSORT subitem 4b? *

Copy and paste relevant sections from the manuscript (include quotes in quotation marks "like this" to indicate direct quotes from your manuscript), or elaborate on this item by providing additional information not in the ms, or briefly explain why the item is not applicable/relevant for your study

"This open-label randomized controlled trial (RCT) conducted between July, 2020 and January, 2021."
"Participants were recruited at the Endocrinology and Metabolism Clinic of Wan-Fang Hospital (Taipei, Taiwan) by physicians."

4b-i) Report if outcomes were (self-)assessed through online questionnaires

Clearly report if outcomes were (self-)assessed through online questionnaires (as common in web-based trials) or otherwise.

	1	2	3	4	5	
subitem not at all important	○	○	○	◉	○	essential

清除選取的項目

Does your paper address subitem 4b-i? *

Copy and paste relevant sections from the manuscript (include quotes in quotation marks "like this" to indicate direct quotes from your manuscript), or elaborate on this item by providing additional information not in the ms, or briefly explain why the item is not applicable/relevant for your study

"Self-reported, face-to-face questionnaires were used to measure HbA1C, patients' knowledge about diabetes, self-care activities, attitudes towards diabetes and health literacy. All data, except for health literacy, were collected at baseline and at 12 weeks at patients' clinic visits."

4b-ii) Report how institutional affiliations are displayed

Report how institutional affiliations are displayed to potential participants [on ehealth media], as affiliations with prestigious hospitals or universities may affect volunteer rates, use, and reactions with regards to an intervention.(Not a required item – describe only if this may bias results)

	1	2	3	4	5	
subitem not at all important	○	○	◉	○	○	essential

清除選取的項目

Does your paper address subitem 4b-ii?

Copy and paste relevant sections from the manuscript (include quotes in quotation marks "like this" to indicate direct quotes from your manuscript), or elaborate on this item by providing additional information not in the ms, or briefly explain why the item is not applicable/relevant for your study

In the written IRB informed consent, patients had already known the institutional affiliation.

5) The interventions for each group with sufficient details to allow replication, including how and when they were actually administered

5-i) Mention names, credential, affiliations of the developers, sponsors, and owners

Mention names, credential, affiliations of the developers, sponsors, and owners [6] (if authors/evaluators are owners or developer of the software, this needs to be declared in a "Conflict of interest" section or mentioned elsewhere in the manuscript).

	1	2	3	4	5	
subitem not at all important	○	○	○	○	◉	essential

清除選取的項目

Does your paper address subitem 5-i?

Copy and paste relevant sections from the manuscript (include quotes in quotation marks "like this" to indicate direct quotes from your manuscript), or elaborate on this item by providing additional information not in the ms, or briefly explain why the item is not applicable/relevant for your study

"The TMU-LOVE program was developed by the TMU School of Pharmacy, and described in Figure 1."

5-ii) Describe the history/development process

Describe the history/development process of the application and previous formative evaluations (e.g., focus groups, usability testing), as these will have an impact on adoption/use rates and help with interpreting results.

	1	2	3	4	5	
subitem not at all important	○	○	○	◉	○	essential

清除選取的項目

Does your paper address subitem 5-ii?

Copy and paste relevant sections from the manuscript (include quotes in quotation marks "like this" to indicate direct quotes from your manuscript), or elaborate on this item by providing additional information not in the ms, or briefly explain why the item is not applicable/relevant for your study

"The TMU-LOVE program was developed by the TMU School of Pharmacy, and described in Figure 1. In the first phase of development, a panel consisting of an endocrinologist, two pharmacists, and a pharmacy professor developed the outline of the program according to the Taiwanese Association of Diabetes Educators (TADE) and seven key points in the AADE7™ [2]. The outline contained five sections, including understanding diabetes, daily care, nutrition care, diabetes drugs, and quizzes. The elements of each sections were also determined."
"A group of pharmacy students joined the second phase to create the videos. The students started to write scenarios and discussed each video with the pharmacy professor and pharmacist. After checking the evidence for the scenarios, the students produced the videos by animations or filming. The videos were reviewed and revised by the panel until they meet the learning goals in the outline. Fifty-one videos were produced over two years, with ten videos on understanding diabetes, ten videos on daily care, six videos on nutrition care, 21 videos on diabetes drugs, and four videos on diabetes knowledge-related quizzes (Figure 2). The medications described in the program were based on the formulary at Wan Fang Hospital, including metformin, acarbose, dipeptidyl peptidase-IV (DPP-IV) inhibitors, meglitinide, sulfonylurea, thiazolidinedione, sodium-glucose cotransporter-2 (SGLT2) inhibitors, and insulin. All of the videos are listed in Multimedia Appendix 2 and are also available at the website of the TMU School of Pharmacy [19]."

5-iii) Revisions and updating

Revisions and updating. Clearly mention the date and/or version number of the application/intervention (and comparator, if applicable) evaluated, or describe whether the intervention underwent major changes during the evaluation process, or whether the development and/or content was “frozen” during the trial. Describe dynamic components such as news feeds or changing content which may have an impact on the replicability of the intervention (for unexpected events see item 3b).

	1	2	3	4	5	
subitem not at all important	○	○	◉	○	○	essential

清除選取的項目

Does your paper address subitem 5-iii?

Copy and paste relevant sections from the manuscript (include quotes in quotation marks "like this" to indicate direct quotes from your manuscript), or elaborate on this item by providing additional information not in the ms, or briefly explain why the item is not applicable/relevant for your study

This program used commercial social media and was completed without interrupting.

5-iv) Quality assurance methods

Provide information on quality assurance methods to ensure accuracy and quality of information provided [1], if applicable.

	1	2	3	4	5	
subitem not at all important	○	○	○	◉	○	essential

清除選取的項目

Does your paper address subitem 5-iv?

Copy and paste relevant sections from the manuscript (include quotes in quotation marks "like this" to indicate direct quotes from your manuscript), or elaborate on this item by providing additional information not in the ms, or briefly explain why the item is not applicable/relevant for your study

"The TMU-LOVE program was developed by the TMU School of Pharmacy, and described in Figure 1. In the first phase of development, a panel consisting of an endocrinologist, two pharmacists, and a pharmacy professor developed the outline of the program according to the Taiwanese Association of Diabetes Educators (TADE) and seven key points in the AADE7™ [2]. The outline contained five sections, including understanding diabetes, daily care, nutrition care, diabetes drugs, and quizzes. The elements of each sections were also determined."
"A group of pharmacy students joined the second phase to create the videos. The students started to write scenarios and discussed each video with the pharmacy professor and pharmacist. After checking the evidence for the scenarios, the students produced the videos by animations or filming. The videos were reviewed and revised by the panel until they meet the learning goals in the outline."

5-v) Ensure replicability by publishing the source code, and/or providing screenshots/screen-capture video, and/or providing flowcharts of the algorithms used

Ensure replicability by publishing the source code, and/or providing screenshots/screen-capture video, and/or providing flowcharts of the algorithms used. Replicability (i.e., other researchers should in principle be able to replicate the study) is a hallmark of scientific reporting.

	1	2	3	4	5	
subitem not at all important	○	○	○	○	◉	essential

清除選取的項目

Does your paper address subitem 5-v?

Copy and paste relevant sections from the manuscript (include quotes in quotation marks "like this" to indicate direct quotes from your manuscript), or elaborate on this item by providing additional information not in the ms, or briefly explain why the item is not applicable/relevant for your study

"All of the videos are listed in Multimedia Appendix 2 and are also available at the website of the TMU School of Pharmacy [19]."
URL: http://pharmschool.tmu.edu.tw/activity/index.php?type=20

5-vi) Digital preservation

Digital preservation: Provide the URL of the application, but as the intervention is likely to change or disappear over the course of the years; also make sure the intervention is archived (Internet Archive, webcitation.org, and/or publishing the source code or screenshots/videos alongside the article). As pages behind login screens cannot be archived, consider creating demo pages which are accessible without login.

	1	2	3	4	5	
subitem not at all important	○	○	○	◉	○	essential

清除選取的項目

Does your paper address subitem 5-vi?

Copy and paste relevant sections from the manuscript (include quotes in quotation marks "like this" to indicate direct quotes from your manuscript), or elaborate on this item by providing additional information not in the ms, or briefly explain why the item is not applicable/relevant for your study

URL: http://pharmschool.tmu.edu.tw/activity/index.php?type=20

5-vii) Access

Access: Describe how participants accessed the application, in what setting/context, if they had to pay (or were paid) or not, whether they had to be a member of specific group. If known, describe how participants obtained "access to the platform and Internet" [1]. To ensure access for editors/reviewers/readers, consider to provide a "backdoor" login account or demo mode for reviewers/readers to explore the application (also important for archiving purposes, see vi).

	1	2	3	4	5	
subitem not at all important	○	○	○	◉	○	essential

清除選取的項目

Does your paper address subitem 5-vii? *

Copy and paste relevant sections from the manuscript (include quotes in quotation marks "like this" to indicate direct quotes from your manuscript), or elaborate on this item by providing additional information not in the ms, or briefly explain why the item is not applicable/relevant for your study

The patient assessed the program at their clinic visit.
"In addition to receiving the usual healthcare, the patients allocated to the intervention group were granted access to the TMU-LOVE program through a quick response code (Multimedia Appendix 1)."

5-viii) Mode of delivery, features/functionalities/components of the intervention and comparator, and the theoretical framework

Describe mode of delivery, features/functionalities/components of the intervention and comparator, and the theoretical framework [6] used to design them (instructional strategy [1], behaviour change techniques, persuasive features, etc., see e.g., [7, 8] for terminology). This includes an in-depth description of the content (including where it is coming from and who developed it) [1],” whether [and how] it is tailored to individual circumstances and allows users to track their progress and receive feedback” [6]. This also includes a description of communication delivery channels and – if computer-mediated communication is a component – whether communication was synchronous or asynchronous [6]. It also includes information on presentation strategies [1], including page design principles, average amount of text on pages, presence of hyperlinks to other resources, etc. [1].

	1	2	3	4	5	
subitem not at all important	○	○	○	○	◉	essential

清除選取的項目

Does your paper address subitem 5-viii? *

Copy and paste relevant sections from the manuscript (include quotes in quotation marks "like this" to indicate direct quotes from your manuscript), or elaborate on this item by providing additional information not in the ms, or briefly explain why the item is not applicable/relevant for your study

"The program was designed to have a duration of 12 week, with two or three videos sent every week and a care message sent every 2 weeks to the patients in the intervention group. All patients received the same videos except for the videos on medications. Videos regarding a basic understanding of diabetes were delivered in weeks 1~4. Videos on daily care started to be delivered in week 5, with six nutrition care videos scheduled over six different weeks. Each patient received the videos for their individual medications in weeks 4, 6, 7, 11, and 12. Four quizzes were scheduled in weeks 3, 6, 9, and 12, (Figure 3). Patients could also access all of the videos through TMU-LOVE whenever they wanted."

5-ix) Describe use parameters

Describe use parameters (e.g., intended “doses” and optimal timing for use). Clarify what instructions or recommendations were given to the user, e.g., regarding timing, frequency, heaviness of use, if any, or was the intervention used ad libitum.

	1	2	3	4	5	
subitem not at all important	○	○	◉	○	○	essential

清除選取的項目

Does your paper address subitem 5-ix?

Copy and paste relevant sections from the manuscript (include quotes in quotation marks "like this" to indicate direct quotes from your manuscript), or elaborate on this item by providing additional information not in the ms, or briefly explain why the item is not applicable/relevant for your study

"The program was designed to have a duration of 12 week, with two or three videos sent every week and a care message sent every 2 weeks to the patients in the intervention group."
"The utility rate was used to represent the frequency of watching videos in each of the five categories. The number of views of each video was divided by the number of target patients. Videos with a calculated utility rate above 100% were counted as 100%."

5-x) Clarify the level of human involvement

Clarify the level of human involvement (care providers or health professionals, also technical assistance) in the e-intervention or as co-intervention (detail number and expertise of professionals involved, if any, as well as “type of assistance offered, the timing and frequency of the support, how it is initiated, and the medium by which the assistance is delivered”. It may be necessary to distinguish between the level of human involvement required for the trial, and the level of human involvement required for a routine application outside of a RCT setting (discuss under item 21 – generalizability).

	1	2	3	4	5	
subitem not at all important	○	○	○	◉	○	essential

清除選取的項目

Does your paper address subitem 5-x?

Copy and paste relevant sections from the manuscript (include quotes in quotation marks "like this" to indicate direct quotes from your manuscript), or elaborate on this item by providing additional information not in the ms, or briefly explain why the item is not applicable/relevant for your study

"A total of 246 patients from the Endocrinology and Metabolism Clinic of Wan-Fang Hospital were screened for this study."
"Self-reported, face-to-face questionnaires were used to measure HbA1C, patients’ knowledge about diabetes, self-care activities, attitudes towards diabetes and health literacy. All data, except for health literacy, were collected at baseline and at 12 weeks at patients’ clinic visits."

5-xi) Report any prompts/reminders used

Report any prompts/reminders used: Clarify if there were prompts (letters, emails, phone calls, SMS) to use the application, what triggered them, frequency etc. It may be necessary to distinguish between the level of prompts/reminders required for the trial, and the level of prompts/reminders for a routine application outside of a RCT setting (discuss under item 21 – generalizability).

	1	2	3	4	5	
subitem not at all important	○	○	○	◉	○	essential

清除選取的項目

Does your paper address subitem 5-xi? *

Copy and paste relevant sections from the manuscript (include quotes in quotation marks "like this" to indicate direct quotes from your manuscript), or elaborate on this item by providing additional information not in the ms, or briefly explain why the item is not applicable/relevant for your study

"The program was designed to have a duration of 12 week, with two or three videos sent every week and a care message sent every 2 weeks to the patients in the intervention group."
"The patients could use the messaging feature of Line to ask questions, and pharmacists would answer by text message or voice call via TMU-LOVE."

5-xii) Describe any co-interventions (incl. training/support)

Describe any co-interventions (incl. training/support): Clearly state any interventions that are provided in addition to the targeted eHealth intervention, as ehealth intervention may not be designed as stand-alone intervention. This includes training sessions and support [1]. It may be necessary to distinguish between the level of training required for the trial, and the level of training for a routine application outside of a RCT setting (discuss under item 21 – generalizability.

	1	2	3	4	5	
subitem not at all important	○	○	◉	○	○	essential

清除選取的項目

Does your paper address subitem 5-xii? *

Copy and paste relevant sections from the manuscript (include quotes in quotation marks "like this" to indicate direct quotes from your manuscript), or elaborate on this item by providing additional information not in the ms, or briefly explain why the item is not applicable/relevant for your study

"Due to the nature of diabetes, the patients in both groups were scheduled to return to the clinic at the end of the 3-month study period, where their monthly prescription was refilled."
"The diabetes health education proposal for the control group was the usual diabetes health care, including patient consultations with physicians, and nurses in the outpatient services, and medication consultations with pharmacists upon receiving prescriptions. Physicians also referred patients for consultations with certified diabetes educators (CDE) when necessary. In addition to receiving the usual healthcare, the patients allocated to the intervention group were granted access to the TMU-LOVE program through a quick response code (Multimedia Appendix 1)."

6a) Completely defined pre-specified primary and secondary outcome measures, including how and when they were assessed

Does your paper address CONSORT subitem 6a? *

Copy and paste relevant sections from the manuscript (include quotes in quotation marks "like this" to indicate direct quotes from your manuscript), or elaborate on this item by providing additional information not in the ms, or briefly explain why the item is not applicable/relevant for your study

"Self-reported, face-to-face questionnaires were used to measure HbA1C, patients' knowledge about diabetes, self-care activities, attitudes towards diabetes and health literacy. All data, except for health literacy, were collected at baseline and at 12 weeks at patients' clinic visits. The questionnaires utilized in this study were composed of five parts, including personal information, the Simplified True/False version of the Diabetes Knowledge Scale (SDKS) [20], Summary of Diabetes Self Care Activities (SDSCA) [22], Diabetes Care Profile-Attitudes Toward Diabetes Scales (DCP-ATDS) [21], and Newest Vital Sign (NVS) [25]. The validated Chinese version of the scales were used in this study [21, 23, 24, 26]."
"Patients' knowledge about diabetes was measured using the True/False version of the SDKS, which contained 24 items and was validated in previous studies [20,21]. Patients' self-care activities were measured using the SDSCA, which consists of ten items and was previously validated [22, 23]. The SDSCA asks patients how many days per week they had performed the correct behaviors regarding medication adherence, diet, exercise, self-monitoring of blood glucose, and foot care. Patients' attitudes towards diabetes were assessed using the DCP-ATDS questionnaire, which was previously validated and translated [21, 24]; the 17 items are divided into positive attitudes, negative attitudes, self-care ability, self-care adherence, and importance of care. Each item is graded on a five-point Likert scale as 'strongly agree', 'agree', 'neutral', 'disagree', or 'strongly disagree'. We used the NVS, which was developed and translated in previous studies [25, 26], to assess health literacy at baseline; this scale consists of six questions based on the nutrition label of an ice-cream product to evaluate both the reading and numeracy of the patients."

6a-i) Online questionnaires: describe if they were validated for online use and apply CHERRIES items to describe how the questionnaires were designed/deployed

If outcomes were obtained through online questionnaires, describe if they were validated for online use and apply CHERRIES items to describe how the questionnaires were designed/deployed [9].

	1	2	3	4	5	
subitem not at all important	○	○	◉	○	○	essential

清除選取的項目

Does your paper address subitem 6a-i?

Copy and paste relevant sections from manuscript text

"Self-reported, face-to-face questionnaires were used to measure HbA1C, patients' knowledge about diabetes, self-care activities, attitudes towards diabetes and health literacy. All data, except for health literacy, were collected at baseline and at 12 weeks at patients' clinic visits."

6a-ii) Describe whether and how "use" (including intensity of use/dosage) was defined/measured/monitored

Describe whether and how "use" (including intensity of use/dosage) was defined/measured/monitored (logins, logfile analysis, etc.). Use/adoption metrics are important process outcomes that should be reported in any ehealth trial.

	1	2	3	4	5	
subitem not at all important	○	○	○	◉	○	essential

清除選取的項目

Does your paper address subitem 6a-ii?

Copy and paste relevant sections from manuscript text

"The utility rate was used to represent the frequency of watching videos in each of the five categories. The number of views of each video was divided by the number of target patients. Videos with a calculated utility rate above 100% were counted as 100%."

6a-iii) Describe whether, how, and when qualitative feedback from participants was obtained

Describe whether, how, and when qualitative feedback from participants was obtained (e.g., through emails, feedback forms, interviews, focus groups).

	1	2	3	4	5	
subitem not at all important	○	○	◉	○	○	essential

清除選取的項目

Does your paper address subitem 6a-iii?

Copy and paste relevant sections from manuscript text

"Patients could also click on the menu of the six icons to connect to the website of the TMU-LOVE program (Figure 4C). The patients could use the messaging feature of Line to ask questions, and pharmacists would answer by text message or voice call via TMU-LOVE."

6b) Any changes to trial outcomes after the trial commenced, with reasons

Does your paper address CONSORT subitem 6b? *

Copy and paste relevant sections from the manuscript (include quotes in quotation marks "like this" to indicate direct quotes from your manuscript), or elaborate on this item by providing additional information not in the ms, or briefly explain why the item is not applicable/relevant for your study

"The TMU-LOVE program effectively improved patients' attitudes towards diabetes. The educational videos covered a variety of essential aspects of diabetes care, including elements of the AADE7™ and drug information. All of the videos were presented with encouraging positive attitudes for patients to fight against diabetes. The program bridged the communication gap between patients and health-care professionals. A previous study that utilized mobile text messaging did not significantly benefit diabetes patients' attitudes [27], while a study using mobile texts, voice messages, and animations improved in patients' attitudes [28]. Delivering videos through social media in the present study significantly improved the attitude of the patients with diabetes, indicating the mode of delivery significantly influences the effectiveness of patient education."

7a) How sample size was determined

NPT: When applicable, details of whether and how the clustering by care provides or centers was addressed

7a-i) Describe whether and how expected attrition was taken into account when calculating the sample size

Describe whether and how expected attrition was taken into account when calculating the sample size.

	1	2	3	4	5	
subitem not at all important	○	○	○	○	◉	essential

清除選取的項目

Does your paper address subitem 7a-i?

Copy and paste relevant sections from manuscript title (include quotes in quotation marks "like this" to indicate direct quotes from your manuscript), or elaborate on this item by providing additional information not in the ms, or briefly explain why the item is not applicable/relevant for your study

"Based on a power of 80% and two-sided alpha, the required sample size was estimated to be at least 128 participants using G-power (vers. 3.1, Heinrich-Heine-Universität Düsseldorf, Germany) [17]. To adjust for an estimated non-response rate of 20%, the final sample size was specified as at least 160 participants, with 80 patients in each group."

7b) When applicable, explanation of any interim analyses and stopping guidelines

Does your paper address CONSORT subitem 7b? *

Copy and paste relevant sections from the manuscript (include quotes in quotation marks "like this" to indicate direct quotes from your manuscript), or elaborate on this item by providing additional information not in the ms, or briefly explain why the item is not applicable/relevant for your study

No, there was not any interim analyses and stopping guidelines.

8a) Method used to generate the random allocation sequence

NPT: When applicable, how care providers were allocated to each trial group

Does your paper address CONSORT subitem 8a? *

Copy and paste relevant sections from the manuscript (include quotes in quotation marks "like this" to indicate direct quotes from your manuscript), or elaborate on this item by providing additional information not in the ms, or briefly explain why the item is not applicable/relevant for your study

"The pharmacists allocated participants in a 1:1 ratio to either the control or intervention group according to a random sequence, which was generated by a digital program before launching the study."

8b) Type of randomisation; details of any restriction (such as blocking and block size)

Does your paper address CONSORT subitem 8b? *

Copy and paste relevant sections from the manuscript (include quotes in quotation marks "like this" to indicate direct quotes from your manuscript), or elaborate on this item by providing additional information not in the ms, or briefly explain why the item is not applicable/relevant for your study

The study was without blocking and stratification. The initial sample size of the study was estimated to be 160, with homogeneous characteristics in type 2 diabetes. The stratified randomization was not adapted. It could be used for our future study. Table 1 demonstrated that most of the characteristics including age, gender, education level, economic status, annual income, health literacy was not significantly different between the control and intervention groups.

9) Mechanism used to implement the random allocation sequence (such as sequentially numbered containers), describing any steps taken to conceal the sequence until interventions were assigned

Does your paper address CONSORT subitem 9? *

Copy and paste relevant sections from the manuscript (include quotes in quotation marks "like this" to indicate direct quotes from your manuscript), or elaborate on this item by providing additional information not in the ms, or briefly explain why the item is not applicable/relevant for your study

The study was an open-label randomized controlled trial.

10) Who generated the random allocation sequence, who enrolled participants, and who assigned participants to interventions

Does your paper address CONSORT subitem 10? *

Copy and paste relevant sections from the manuscript (include quotes in quotation marks "like this" to indicate direct quotes from your manuscript), or elaborate on this item by providing additional information not in the ms, or briefly explain why the item is not applicable/relevant for your study

"Participants were recruited at the Endocrinology and Metabolism Clinic of Wan-Fang Hospital (Taipei, Taiwan) by physicians. The pharmacists allocated participants in a 1:1 ratio to either the control or intervention group according to a random sequence, which was generated by a digital program before launching the study."

11a) If done, who was blinded after assignment to interventions (for example, participants, care providers, those assessing outcomes) and how

NPT: Whether or not administering co-interventions were blinded to group assignment

11a-i) Specify who was blinded, and who wasn’t

Specify who was blinded, and who wasn’t. Usually, in web-based trials it is not possible to blind the participants [1, 3] (this should be clearly acknowledged), but it may be possible to blind outcome assessors, those doing data analysis or those administering co-interventions (if any).

	1	2	3	4	5	
subitem not at all important	○	○	○	○	◉	essential

清除選取的項目

Does your paper address subitem 11a-i? *

Copy and paste relevant sections from the manuscript (include quotes in quotation marks "like this" to indicate direct quotes from your manuscript), or elaborate on this item by providing additional information not in the ms, or briefly explain why the item is not applicable/relevant for your study

This study was unblinded due to its feasibility.

11a-ii) Discuss e.g., whether participants knew which intervention was the "intervention of interest" and which one was the "comparator"

Informed consent procedures (4a-ii) can create biases and certain expectations - discuss e.g., whether participants knew which intervention was the "intervention of interest" and which one was the "comparator".

	1	2	3	4	5	
subitem not at all important	○	○	○	◉	○	essential

清除選取的項目

Does your paper address subitem 11a-ii?

Copy and paste relevant sections from the manuscript (include quotes in quotation marks "like this" to indicate direct quotes from your manuscript), or elaborate on this item by providing additional information not in the ms, or briefly explain why the item is not applicable/relevant for your study

This study was unblinded due to its feasibility.
The intervention included the social media based TMU-LOVE and usual care.
The control included only usual care.

11b) If relevant, description of the similarity of interventions

(this item is usually not relevant for ehealth trials as it refers to similarity of a placebo or sham intervention to a active medication/intervention)

Does your paper address CONSORT subitem 11b? *

Copy and paste relevant sections from the manuscript (include quotes in quotation marks "like this" to indicate direct quotes from your manuscript), or elaborate on this item by providing additional information not in the ms, or briefly explain why the item is not applicable/relevant for your study

No, there was not any description of the similarity of interventions.

12a) Statistical methods used to compare groups for primary and secondary outcomes

NPT: When applicable, details of whether and how the clustering by care providers or centers was addressed

Does your paper address CONSORT subitem 12a? *

Copy and paste relevant sections from the manuscript (include quotes in quotation marks "like this" to indicate direct quotes from your manuscript), or elaborate on this item by providing additional information not in the ms, or briefly explain why the item is not applicable/relevant for your study

"Statistical analyses were conducted using SPSS (SPSS, Released 2009. PASW Statistics for Windows, vers. 18.0. Chicago, IL, USA). The study was performed following a per protocol analysis with an intention-to-treat sensitivity analysis for the primary outcomes. All tests were two-tailed, with a significance level of 0.05. Differences in baseline characteristics were examined using descriptive analysis. The Kolmogorov-Smirnov test was used to ensure the normality of the distributions of the study outcomes. Paired t-tests were used to examine the differences in the pretest and post-test scores within a group, while unpaired t-tests were performed to compare the differences between groups."

12a-i) Imputation techniques to deal with attrition / missing values

Imputation techniques to deal with attrition / missing values: Not all participants will use the intervention/comparator as intended and attrition is typically high in ehealth trials. Specify how participants who did not use the application or dropped out from the trial were treated in the statistical analysis (a complete case analysis is strongly discouraged, and simple imputation techniques such as LOCF may also be problematic [4]).

	1	2	3	4	5	
subitem not at all important	○	○	○	◉	○	essential

清除選取的項目

Does your paper address subitem 12a-i? *

Copy and paste relevant sections from the manuscript (include quotes in quotation marks "like this" to indicate direct quotes from your manuscript), or elaborate on this item by providing additional information not in the ms, or briefly explain why the item is not applicable/relevant for your study

No imputation techniques to deal with attrition / missing values in this study.

12b) Methods for additional analyses, such as subgroup analyses and adjusted analyses

Does your paper address CONSORT subitem 12b? *

Copy and paste relevant sections from the manuscript (include quotes in quotation marks "like this" to indicate direct quotes from your manuscript), or elaborate on this item by providing additional information not in the ms, or briefly explain why the item is not applicable/relevant for your study

"Subgroup analysis related to health literacy was performed with logistic regression according to the results of Kolmogorov-Smirnov test."

X26) REB/IRB Approval and Ethical Considerations [recommended as subheading under "Methods"] (not a CONSORT item)

X26-i) Comment on ethics committee approval

	1	2	3	4	5	
subitem not at all important	○	○	○	○	◉	essential

清除選取的項目

Does your paper address subitem X26-i?

Copy and paste relevant sections from the manuscript (include quotes in quotation marks "like this" to indicate direct quotes from your manuscript), or elaborate on this item by providing additional information not in the ms, or briefly explain why the item is not applicable/relevant for your study

"This open-label randomized controlled trial (RCT) conducted between July, 2020 and January, 2021, received ethical approval from the Taipei Medical University Jointed Institutional Review Boards (TMU-JIRB No.: N201905088) and was registered with Clinical-Trial.gov (NCT04876274)."

x26-ii) Outline informed consent procedures

Outline informed consent procedures e.g., if consent was obtained offline or online (how? Checkbox, etc.?), and what information was provided (see 4a-ii). See [6] for some items to be included in informed consent documents.

	1	2	3	4	5	
subitem not at all important	○	○	○	◉	○	essential

清除選取的項目

Does your paper address subitem X26-ii?

Copy and paste relevant sections from the manuscript (include quotes in quotation marks "like this" to indicate direct quotes from your manuscript), or elaborate on this item by providing additional information not in the ms, or briefly explain why the item is not applicable/relevant for your study

Written informed consent with 6 pages according to TMU-JIRB.

X26-iii) Safety and security procedures

Safety and security procedures, incl. privacy considerations, and any steps taken to reduce the likelihood or detection of harm (e.g., education and training, availability of a hotline)

	1	2	3	4	5	
subitem not at all important	○	○	○	◉	○	essential

清除選取的項目

Does your paper address subitem X26-iii?

Copy and paste relevant sections from the manuscript (include quotes in quotation marks "like this" to indicate direct quotes from your manuscript), or elaborate on this item by providing additional information not in the ms, or briefly explain why the item is not applicable/relevant for your study

Safety and security procedures were according to the privacy and security codes of TMU-JIRB. The questionnaires will be kept for 3 years after intervention.

RESULTS

13a) For each group, the numbers of participants who were randomly assigned, received intended treatment, and were analysed for the primary outcome

NPT: The number of care providers or centers performing the intervention in each group and the number of patients treated by each care provider in each center

Does your paper address CONSORT subitem 13a? *

Copy and paste relevant sections from the manuscript (include quotes in quotation marks "like this" to indicate direct quotes from your manuscript), or elaborate on this item by providing additional information not in the ms, or briefly explain why the item is not applicable/relevant for your study

"164 patients—82 in the intervention group and 82 in the control group—were included in the analysis at the end of this study."

13b) For each group, losses and exclusions after randomisation, together with reasons

Does your paper address CONSORT subitem 13b? (NOTE: Preferably, this is shown in a CONSORT flow diagram) *

Copy and paste relevant sections from the manuscript (include quotes in quotation marks "like this" to indicate direct quotes from your manuscript), or elaborate on this item by providing additional information not in the ms, or briefly explain why the item is not applicable/relevant for your study

"A total of 246 patients from the Endocrinology and Metabolism Clinic of Wan-Fang Hospital were screened for this study. Ten patients had a HbA1C of < 6%, three patients did not complete the informed consent form, and 52 declined to participate; all of these patients were excluded from the study. In total 181 patients were randomized equally into the control or intervention groups (Figure 5). There were Nine and eight subjects from the intervention and control groups, respectively, were lost follow up; thus 164 patients—82 in the intervention group and 82 in the control group—were included in the analysis at the end of this study."

13b-i) Attrition diagram

Strongly recommended: An attrition diagram (e.g., proportion of participants still logging in or using the intervention/comparator in each group plotted over time, similar to a survival curve) or other figures or tables demonstrating usage/dose/engagement.

	1	2	3	4	5	
subitem not at all important	○	○	○	○	◉	essential

清除選取的項目

Does your paper address subitem 13b-i?

Copy and paste relevant sections from the manuscript or cite the figure number if applicable (include quotes in quotation marks "like this" to indicate direct quotes from your manuscript), or elaborate on this item by providing additional information not in the ms, or briefly explain why the item is not applicable/relevant for your study

The attrition diagram was demonstrated in figure 5.

14a) Dates defining the periods of recruitment and follow-up

Does your paper address CONSORT subitem 14a? *

Copy and paste relevant sections from the manuscript (include quotes in quotation marks "like this" to indicate direct quotes from your manuscript), or elaborate on this item by providing additional information not in the ms, or briefly explain why the item is not applicable/relevant for your study

"This open-label randomized controlled trial (RCT) conducted between July, 2020 and January, 2021."

14a-i) Indicate if critical “secular events” fell into the study period

Indicate if critical “secular events” fell into the study period, e.g., significant changes in Internet resources available or “changes in computer hardware or Internet delivery resources”

	1	2	3	4	5	
subitem not at all important	○	○	○	◉	○	essential

清除選取的項目

Does your paper address subitem 14a-i?

Copy and paste relevant sections from the manuscript (include quotes in quotation marks "like this" to indicate direct quotes from your manuscript), or elaborate on this item by providing additional information not in the ms, or briefly explain why the item is not applicable/relevant for your study

There was no secular events occurred in the study period.

14b) Why the trial ended or was stopped (early)

Does your paper address CONSORT subitem 14b? *

Copy and paste relevant sections from the manuscript (include quotes in quotation marks "like this" to indicate direct quotes from your manuscript), or elaborate on this item by providing additional information not in the ms, or briefly explain why the item is not applicable/relevant for your study

The trial ended as expected.

15) A table showing baseline demographic and clinical characteristics for each group

NPT: When applicable, a description of care providers (case volume, qualification, expertise, etc.) and centers (volume) in each group

Does your paper address CONSORT subitem 15? *

Copy and paste relevant sections from the manuscript (include quotes in quotation marks "like this" to indicate direct quotes from your manuscript), or elaborate on this item by providing additional information not in the ms, or briefly explain why the item is not applicable/relevant for your study

"Table 1 shows the demographic characteristics of the patients"

15-i) Report demographics associated with digital divide issues

In ehealth trials it is particularly important to report demographics associated with digital divide issues, such as age, education, gender, social-economic status, computer/Internet/ehealth literacy of the participants, if known.

	1	2	3	4	5	
subitem not at all important	○	○	○	◉	○	essential

清除選取的項目

Does your paper address subitem 15-i? *

Copy and paste relevant sections from the manuscript (include quotes in quotation marks "like this" to indicate direct quotes from your manuscript), or elaborate on this item by providing additional information not in the ms, or briefly explain why the item is not applicable/relevant for your study

"The mean age of the participants was 58.37 ± 11.77 years, with 67.1% and 76.8% males in the control and intervention groups, respectively. More than half of the participants were highly educated."
The social economic status were also listed in table 1. The study included the participants who were Line-user. The eHealth literacy level was not measured.

16) For each group, number of participants (denominator) included in each analysis and whether the analysis was by original assigned groups

16-i) Report multiple “denominators” and provide definitions

Report multiple “denominators” and provide definitions: Report N’s (and effect sizes) “across a range of study participation [and use] thresholds” [1], e.g., N exposed, N consented, N used more than x times, N used more than y weeks, N participants “used” the intervention/comparator at specific pre-defined time points of interest (in absolute and relative numbers per group). Always clearly define “use” of the intervention.

	1	2	3	4	5	
subitem not at all important	○	○	○	◉	○	essential

清除選取的項目

Does your paper address subitem 16-i? *

Copy and paste relevant sections from the manuscript (include quotes in quotation marks "like this" to indicate direct quotes from your manuscript), or elaborate on this item by providing additional information not in the ms, or briefly explain why the item is not applicable/relevant for your study

Number of participants of the intervention and control groups in each analysis was the same as arranged.
"In total 181 patients were randomized equally into the control or intervention groups (Figure 5). There were Nine and eight subjects from the intervention and control groups, respectively, were lost follow up; thus 164 patients—82 in the intervention group and 82 in the control group—were included in the analysis at the end of this study."

16-ii) Primary analysis should be intent-to-treat

Primary analysis should be intent-to-treat, secondary analyses could include comparing only “users”, with the appropriate caveats that this is no longer a randomized sample (see 18-i).

	1	2	3	4	5	
subitem not at all important	○	○	○	◉	○	essential

清除選取的項目

Does your paper address subitem 16-ii?

Copy and paste relevant sections from the manuscript (include quotes in quotation marks "like this" to indicate direct quotes from your manuscript), or elaborate on this item by providing additional information not in the ms, or briefly explain why the item is not applicable/relevant for your study

"This study employed a per protocol analysis (Table 2 and Figure 5)."
"Sensitivity analysis by intention-to-treat showed similar trends for the HbA1C levels, which changed from 6.93% ± 0.84% to 7.00% ± 0.88% in the intervention group (P=.34) and 6.73% ± 0.64% to 6.74% ± 0.68% in the control group (P=.91)."

17a) For each primary and secondary outcome, results for each group, and the estimated effect size and its precision (such as 95% confidence interval)

Does your paper address CONSORT subitem 17a? *

Copy and paste relevant sections from the manuscript (include quotes in quotation marks "like this" to indicate direct quotes from your manuscript), or elaborate on this item by providing additional information not in the ms, or briefly explain why the item is not applicable/relevant for your study

All the data was reported with its estimated effect size (95% confidence interval or standard deviation).

17a-i) Presentation of process outcomes such as metrics of use and intensity of use

In addition to primary/secondary (clinical) outcomes, the presentation of process outcomes such as metrics of use and intensity of use (dose, exposure) and their operational definitions is critical. This does not only refer to metrics of attrition (13-b) (often a binary variable), but also to more continuous exposure metrics such as “average session length”. These must be accompanied by a technical description how a metric like a “session” is defined (e.g., timeout after idle time) [1] (report under item 6a).

	1	2	3	4	5	
subitem not at all important	○	○	○	◉	○	essential

清除選取的項目

Does your paper address subitem 17a-i?

Copy and paste relevant sections from the manuscript (include quotes in quotation marks "like this" to indicate direct quotes from your manuscript), or elaborate on this item by providing additional information not in the ms, or briefly explain why the item is not applicable/relevant for your study

"Among the 51 videos in the TMU-LOVE intervention, the utility rates of drug-related videos were the highest, which implies a demand for medication information among patients."

17b) For binary outcomes, presentation of both absolute and relative effect sizes is recommended

Does your paper address CONSORT subitem 17b? *

Copy and paste relevant sections from the manuscript (include quotes in quotation marks "like this" to indicate direct quotes from your manuscript), or elaborate on this item by providing additional information not in the ms, or briefly explain why the item is not applicable/relevant for your study

"After 3 months of the intervention, the average HbA1C level had insignificantly changed from 6.95% ± 0.87% to 7.03% ± 0.91% (P=.34) in the intervention group and from 6.75% ± 0.63% to 6.76% ± 0.68% (P=.91) in the control group. Sensitivity analysis by intention-to-treat showed similar trends for the HbA1C levels, which changed from 6.93% ± 0.84% to 7.00% ± 0.88% in the intervention group (P=.34) and 6.73% ± 0.64% to 6.74% ± 0.68% in the control group (P=.91)."
"Both groups experienced significant improvements in overall knowledge scores, from 67.74 ± 16.99 vs. 77.33 ± 11.87 (P<.001) and 65.27 ± 18.07 vs. 72.83 ± 12.93 (P<.001) in the intervention and control groups, respectively. However, the difference between the two groups was not significant (intervention vs. control: 9.59 ± 14.47 vs. 7.56 ± 14.90, P=.38). The scores for each item of the SDKS are shown in Multimedia Appendix 3. With respect to the self-care activities measured by the SDSCA, a significant improvement was only observed in the intervention group (change from 3.69 ± 1.26 to 3.98 ± 1.23; P=.02). Table 3 shows the top five reasons mentioned by patients who found it difficult to engage in self-care activities. The intervention group exhibited a significant growth in the overall attitude score from 3.59 ± 0.39 to 3.85 ± 0.44 (P<.001), while no significant change was observed in the control group (3.62 ± 0.40 to 3.71 ± 0.44, P=.11), and the difference in the change between baseline and follow-up was not significant between groups (0.20, 95% CI -0.31 to -0.01, P=.06). Detailed data for the SDSCA and the DCP-ATDS are presented in Multimedia Appendices 4 and 5, respectively."

18) Results of any other analyses performed, including subgroup analyses and adjusted analyses, distinguishing pre-specified from exploratory

Does your paper address CONSORT subitem 18? *

Copy and paste relevant sections from the manuscript (include quotes in quotation marks "like this" to indicate direct quotes from your manuscript), or elaborate on this item by providing additional information not in the ms, or briefly explain why the item is not applicable/relevant for your study

"A regression model was used to assess the effect of low health literacy on knowledge, attitudes, and self-care activities in the two groups (Table 4)."

18-i) Subgroup analysis of comparing only users

A subgroup analysis of comparing only users is not uncommon in ehealth trials, but if done, it must be stressed that this is a self-selected sample and no longer an unbiased sample from a randomized trial (see 16-iii).

	1	2	3	4	5	
subitem not at all important	○	○	◉	○	○	essential

清除選取的項目

Does your paper address subitem 18-i?

Copy and paste relevant sections from the manuscript (include quotes in quotation marks "like this" to indicate direct quotes from your manuscript), or elaborate on this item by providing additional information not in the ms, or briefly explain why the item is not applicable/relevant for your study

The study performed subgroup analysis including the differences within each groups.

19) All important harms or unintended effects in each group

(for specific guidance see CONSORT for harms)

Does your paper address CONSORT subitem 19? *

Copy and paste relevant sections from the manuscript (include quotes in quotation marks "like this" to indicate direct quotes from your manuscript), or elaborate on this item by providing additional information not in the ms, or briefly explain why the item is not applicable/relevant for your study

There was no important harms or unintended effects noted.

19-i) Include privacy breaches, technical problems

Include privacy breaches, technical problems. This does not only include physical “harm” to participants, but also incidents such as perceived or real privacy breaches [1], technical problems, and other unexpected/unintended incidents. “Unintended effects” also includes unintended positive effects [2].

	1	2	3	4	5	
subitem not at all important	○	○	●	○	○	essential

清除選取的項目

Does your paper address subitem 19-i?

Copy and paste relevant sections from the manuscript (include quotes in quotation marks "like this" to indicate direct quotes from your manuscript), or elaborate on this item by providing additional information not in the ms, or briefly explain why the item is not applicable/relevant for your study

This eHealth program, TMU-LOVE, was only used for health education information and did not enter any patients' information. All the data was performed by face-to-face written questionnaires. Therefore, there was no privacy breaches or technical problems.

19-ii) Include qualitative feedback from participants or observations from staff/researchers

Include qualitative feedback from participants or observations from staff/researchers, if available, on strengths and shortcomings of the application, especially if they point to unintended/unexpected effects or uses. This includes (if available) reasons for why people did or did not use the application as intended by the developers.

	1	2	3	4	5	
subitem not at all important	○	○	○	●	○	essential

清除選取的項目

Does your paper address subitem 19-ii?

Copy and paste relevant sections from the manuscript (include quotes in quotation marks "like this" to indicate direct quotes from your manuscript), or elaborate on this item by providing additional information not in the ms, or briefly explain why the item is not applicable/relevant for your study

"Table 3 shows the top five reasons mentioned by patients who found it difficult to engage in self-care activities."

DISCUSSION

22) Interpretation consistent with results, balancing benefits and harms, and considering other relevant evidence

NPT: In addition, take into account the choice of the comparator, lack of or partial blinding, and unequal expertise of care providers or centers in each group

22-i) Restate study questions and summarize the answers suggested by the data, starting with primary outcomes and process outcomes (use)

Restate study questions and summarize the answers suggested by the data, starting with primary outcomes and process outcomes (use).

	1	2	3	4	5	
subitem not at all important	○	○	○	○	◉	essential

清除選取的項目

Does your paper address subitem 22-i? *

Copy and paste relevant sections from the manuscript (include quotes in quotation marks "like this" to indicate direct quotes from your manuscript), or elaborate on this item by providing additional information not in the ms, or briefly explain why the item is not applicable/relevant for your study

"The present study demonstrates that the delivery of TMU-LOVE social media-based education program during COVID-19 pandemic improved the attitudes towards diabetes and self-care activities of patients with type 2 diabetes. Among the 51 videos in the TMU-LOVE intervention, the utility rates of drug-related videos were the highest, which implies a demand for medication information among patients. The study also interestingly found that the video utility rate for quizzes dramatically decreased after the first quiz, making it difficult to confirm the patients' knowledge and perception. Moreover, health literacy did not have a significant impact on the patients' knowledge after exposure to video education through TMU-LOVE, indicating that the program overcame the negative effects low health literacy"

22-ii) Highlight unanswered new questions, suggest future research

Highlight unanswered new questions, suggest future research.

	1	2	3	4	5	
subitem not at all important	○	○	○	○	◉	essential

清除選取的項目

Does your paper address subitem 22-ii?

Copy and paste relevant sections from the manuscript (include quotes in quotation marks "like this" to indicate direct quotes from your manuscript), or elaborate on this item by providing additional information not in the ms, or briefly explain why the item is not applicable/relevant for your study

"We could only determine that patients clicked on the videos, but could not confirm how long and how attentively the patients watched the videos."
"Future studies of more-diverse populations, with a longer study duration, and careful analysis of digital learning metrics are needed to validate the effects of TMU-LOVE among patients with type 2 diabetes."

20) Trial limitations, addressing sources of potential bias, imprecision, and, if relevant, multiplicity of analyses

20-i) Typical limitations in ehealth trials

Typical limitations in ehealth trials: Participants in ehealth trials are rarely blinded. Ehealth trials often look at a multiplicity of outcomes, increasing risk for a Type I error. Discuss biases due to non-use of the intervention/usability issues, biases through informed consent procedures, unexpected events.

	1	2	3	4	5	
subitem not at all important	○	○	○	○	◉	essential

清除選取的項目

Does your paper address subitem 20-i? *

Copy and paste relevant sections from the manuscript (include quotes in quotation marks "like this" to indicate direct quotes from your manuscript), or elaborate on this item by providing additional information not in the ms, or briefly explain why the item is not applicable/relevant for your study

"The intervention only lasted 12 weeks, thus we were unable to assess long-term outcomes. The patients’ attitude, knowledge, and self-care activity scores were collected by self-reporting, which may have created recall bias."

21) Generalisability (external validity, applicability) of the trial findings

NPT: External validity of the trial findings according to the intervention, comparators, patients, and care providers or centers involved in the trial

21-i) Generalizability to other populations

Generalizability to other populations: In particular, discuss generalizability to a general Internet population, outside of a RCT setting, and general patient population, including applicability of the study results for other organizations

	1	2	3	4	5	
subitem not at all important	○	○	○	○	◉	essential

清除選取的項目

Does your paper address subitem 21-i?

Copy and paste relevant sections from the manuscript (include quotes in quotation marks "like this" to indicate direct quotes from your manuscript), or elaborate on this item by providing additional information not in the ms, or briefly explain why the item is not applicable/relevant for your study

"Our study has several limitations. This was a single-center study which may lead to population bias and thus restrict its generalizability."
Future study by multi-center study was required.

21-ii) Discuss if there were elements in the RCT that would be different in a routine application setting

Discuss if there were elements in the RCT that would be different in a routine application setting (e.g., prompts/reminders, more human involvement, training sessions or other co-interventions) and what impact the omission of these elements could have on use, adoption, or outcomes if the intervention is applied outside of a RCT setting.

	1	2	3	4	5	
subitem not at all important	○	◉	○	○	○	essential

清除選取的項目

Does your paper address subitem 21-ii?

Copy and paste relevant sections from the manuscript (include quotes in quotation marks "like this" to indicate direct quotes from your manuscript), or elaborate on this item by providing additional information not in the ms, or briefly explain why the item is not applicable/relevant for your study

The study included patients who were routine Line-user the application of TMU-LOVE program.Those who has no experience to use Line are unknown.

OTHER INFORMATION

23) Registration number and name of trial registry

Does your paper address CONSORT subitem 23? *

Copy and paste relevant sections from the manuscript (include quotes in quotation marks "like this" to indicate direct quotes from your manuscript), or elaborate on this item by providing additional information not in the ms, or briefly explain why the item is not applicable/relevant for your study

"ClinicalTrials.gov NCT04876274."

24) Where the full trial protocol can be accessed, if available

Does your paper address CONSORT subitem 24? *

Cite a Multimedia Appendix, other reference, or copy and paste relevant sections from the manuscript (include quotes in quotation marks "like this" to indicate direct quotes from your manuscript), or elaborate on this item by providing additional information not in the ms, or briefly explain why the item is not applicable/relevant for your study

The full trial protocol was written in Chinese and are available upon research authors.

25) Sources of funding and other support (such as supply of drugs), role of funders

Does your paper address CONSORT subitem 25? *

Copy and paste relevant sections from the manuscript (include quotes in quotation marks "like this" to indicate direct quotes from your manuscript), or elaborate on this item by providing additional information not in the ms, or briefly explain why the item is not applicable/relevant for your study

"This work was supported by funding from the Taiwan Ministry of Health and Welfare (MOHW109-TDU-B-212-114007, MOHW110-TDU-B-212-124007)."

X27) Conflicts of Interest (not a CONSORT item)

X27-i) State the relation of the study team towards the system being evaluated

In addition to the usual declaration of interests (financial or otherwise), also state the relation of the study team towards the system being evaluated, i.e., state if the authors/evaluators are distinct from or identical with the developers/sponsors of the intervention.

	1	2	3	4	5	
subitem not at all important	○	○	○	◉	○	essential

清除選取的項目

Does your paper address subitem X27-i?

Copy and paste relevant sections from the manuscript (include quotes in quotation marks "like this" to indicate direct quotes from your manuscript), or elaborate on this item by providing additional information not in the ms, or briefly explain why the item is not applicable/relevant for your study

This is a principle investigation initiated trial. The research is granted by governmental agency. The sponsors of the study were not involved the study design, system development, or data evaluation.

About the CONSORT EHEALTH checklist

As a result of using this checklist, did you make changes in your manuscript? *

- ○ yes, major changes
- ◉ yes, minor changes
- ○ no

What were the most important changes you made as a result of using this checklist?

The evaluation of development of the TMU-LOVE program in manuscript.

How much time did you spend on going through the checklist INCLUDING making changes in your manuscript *

It took bout 8 hours to complete.

As a result of using this checklist, do you think your manuscript has improved? *

- (●) yes
- ( ) no
- ( ) 其他:

Would you like to become involved in the CONSORT EHEALTH group?

This would involve for example becoming involved in participating in a workshop and writing an "Explanation and Elaboration" document

- ( ) yes
- (●) no
- ( ) 其他:

清除選取的項目

Any other comments or questions on CONSORT EHEALTH

The authors agree to use this CONSORT EHEALTH form for randomized control.

STOP - Save this form as PDF before you click submit

To generate a record that you filled in this form, we recommend to generate a PDF of this page (on a Mac, simply select "print" and then select "print as PDF") before you submit it.

When you submit your (revised) paper to JMIR, please upload the PDF as supplementary file.

Don't worry if some text in the textboxes is cut off, as we still have the complete information in our database. Thank you!

Final step: Click submit !

Click submit so we have your answers in our database!

提交

請勿利用 Google 表單送出密碼。

Google 並未認可或建立這項內容。 檢舉濫用情形 - 服務條款 - 隱私權政策

Google 表單
